# A Bayesian framework for virtual comparative trials and bioequivalence assessments

**DOI:** 10.3389/fphar.2024.1404619

**Published:** 2024-07-30

**Authors:** Frederic Y. Bois, Céline Brochot

**Affiliations:** Certara UK Limited, Certara Predictive Technologies Division, Sheffield, United Kingdom

**Keywords:** Bayesian inference, comparative clinical trials, virtual bioequivalence assessment, paliperidone, sensitivity analysis

## Abstract

**Introduction:**

In virtual bioequivalence (VBE) assessments, pharmacokinetic models informed with *in vitro* data and verified with small clinical trials’ data are used to simulate otherwise unfeasibly large trials. Simulated VBE trials are assessed in a frequentist framework as if they were real despite the unlimited number of virtual subjects they can use. This may adequately control consumer risk but imposes unnecessary risks on producers. We propose a fully Bayesian model-integrated VBE assessment framework that circumvents these limitations.

**Methods:**

We illustrate our approach with a case study on a hypothetical paliperidone palmitate (PP) generic long-acting injectable suspension formulation using a validated population pharmacokinetic model published for the reference formulation. BE testing, study power, type I and type II error analyses or their Bayesian equivalents, and safe-space analyses are demonstrated.

**Results:**

The fully Bayesian workflow is more precise than the frequentist workflow. Decisions about bioequivalence and safe space analyses in the two workflows can differ markedly because the Bayesian analyses are more accurate.

**Discussion:**

A Bayesian framework can adequately control consumer risk and minimize producer risk . It rewards data gathering and model integration to make the best use of prior information. The frequentist approach is less precise but faster to compute, and it can still be used as a first step to narrow down the parameter space to explore in safe-space analyses.

## 1 Introduction

Bioequivalence (BE) clinical trial analyses check that two drug formulations do not lead to different average rates and extents of drug absorption in patient populations or surrogate healthy volunteers. The development of complex bioequivalent products can be challenging. For example, long-acting injectable (LAI) formulations may require clinical trials lasting years; high inter- or intra-subject variability may force the use of very large numbers of trial participants; and costs can be prohibitive for such products. Virtual bioequivalence (VBE) testing uses a simulation model and *in vitro* and abbreviated trial data (obtained from small-sized studies) to generate realistic BE trial simulations, which are then used to assess BE for a particular formulation (Hsieh et al., 2021; Tsakalozou et al., 2021). This can be much less expensive and time-consuming than full BE trials (Sharan et al., 2021; Gong et al., 2023). The motivations and conditions for the US-FDA approval of a generic product on the basis of a VBE assessment instead of a comparative clinical trial were recently explained (Tsakalozou et al., 2021). An extensive validation process was developed on the occasion of that assessment. A publication by Hsieh et al. (2021) described a partly Bayesian VBE workflow integrating evidence from *in vitro* experiments, scientific literature, and clinical trials.

Regulatory acceptance of VBE is quite new, and VBE methodology is still evolving. Fortunately, most elements of a sound VBE framework are available. Modeling and simulations to design or replace clinical trials are common (Tozer et al., 1996; Upton and Mould, 2014; Jamei, 2016; Cristofoletti et al., 2017; Lin and Wong, 2017; Loisios-Konstantinidis et al., 2020; Zhang et al., 2021; Goutelle et al., 2022). Model-integrated BE assessment is progressing rapidly (Dubois et al., 2010; Loingeville et al., 2020; Möllenhoff et al., 2022), and VBE can easily use model-integrated approaches (Gong et al., 2023). Since VBE uses minimal clinical data, it makes sense to integrate historical data and *in vitro* evidence [e.g., on dissolution (Cristofoletti et al., 2018)] to quantify the differences between the reference and generic products. Bayesian inference is currently the best way to do that, even with mechanistic models (Gelman et al., 1996; Bois et al., 2020; Hsieh et al., 2021; Wedagedera et al., 2022). Bayesian analysis of clinical BE trials has been discussed (Fluehler et al., 1982; Selwyn and Hall, 1984; Racine-Poon et al., 1987; Breslow, 1990; Ghosh and Rosner, 2007; Ghosh and Gönen, 2008; Peck and Campbell, 2019).

Yet, so far, simulated VBE trials have been submitted to non-compartmental analyses and standard hypothesis testing as if they were real trials. However, an unlimited sample size is available for a virtual trial. At the limit, standard statistical tests would need to operate with zero-length confidence intervals, rendering error analyses moot. Arbitrarily limiting the size of virtual trials is also sub-optimal for decision making. It lowers power and affects both producers and consumers because a safe product, potentially less expensive, might not be approved even when it could be. Nobody benefits from curtailing the power of VBE assessments. We show that the above difficulties disappear if we adopt a more fully coherent Bayesian approach (Fluehler et al., 1982; Racine-Poon et al., 1987; Breslow, 1990), which shifts from a statistical assessment based on asymptotics to a more coherent probabilistic assessment.

In the following, we describe a fully Bayesian workflow for VBE assessment and compare it with a partly Bayesian workflow. We apply these workflows in a case study using simulated abbreviated trial data. The reference and test formulations will be assumed to differ in terms of a single drug-release parameter. We discuss issues related to model-integrated VBE, power and type I error assessments, and safe space analysis [a form of sensitivity analysis to estimate the range by which a generic formulation’s characteristics can vary while maintaining bioequivalence (Hsieh et al., 2021)].

## 2 Materials and methods

### 2.1 VBE workflows

**FIGURE 1:**
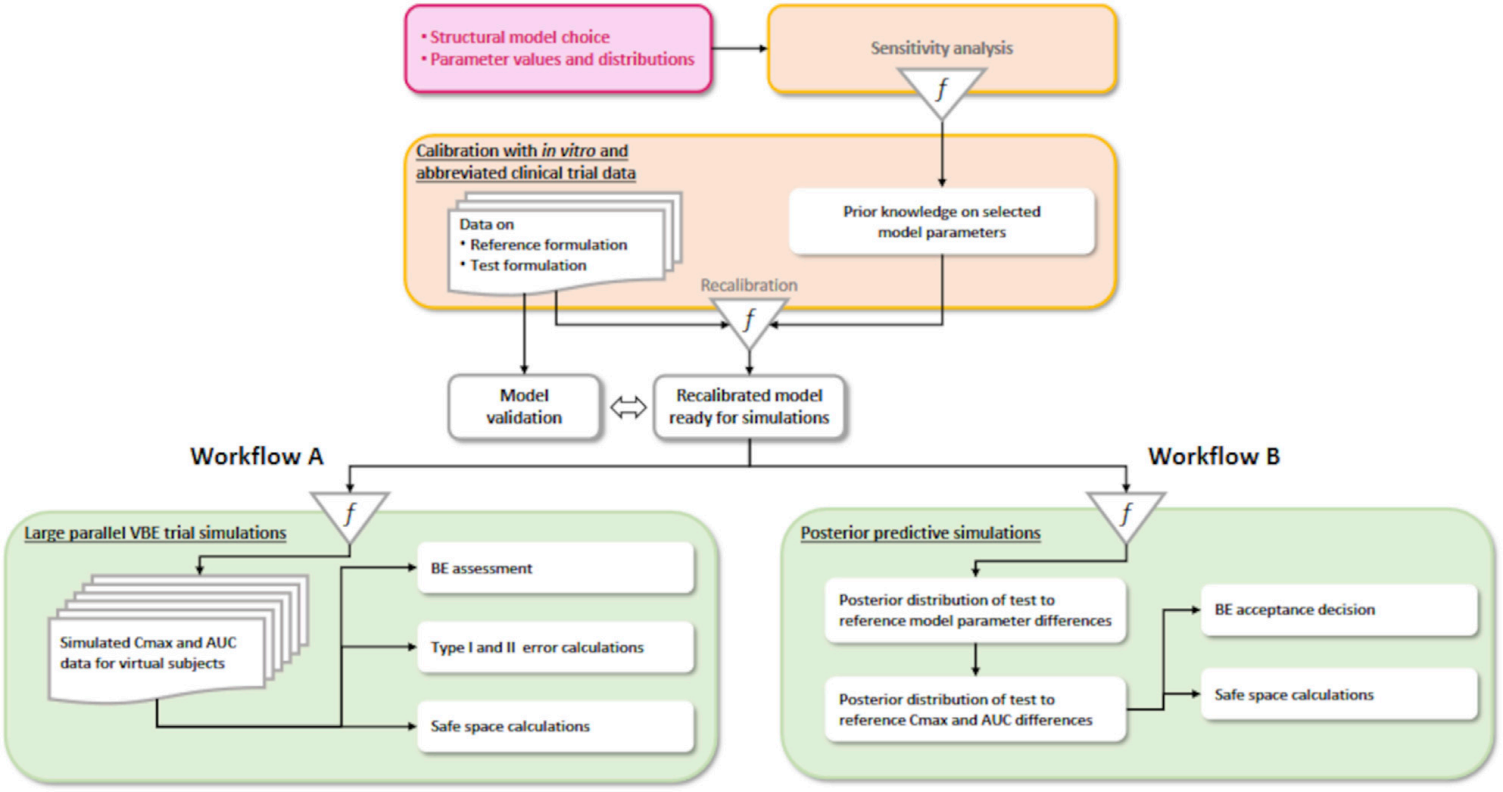
Two VBE workflows. On the left **(A)**, a partly Bayesian data-based assessment workflow, and on the right **(B)**, the fully Bayesian workflow we propose.

We investigate two workflows (Figure 1). The steps of workflow A mimic data-based BE assessment, except for the Bayesian calibration of a predictive model:

1. Definition of a simulation model structure and prior parameter distributions, usually for the reference formulation. Mechanistic or empirical structural models can be used, but mechanistic models should be preferred if *in vitro* data are available. The model should be sufficiently predictive of the key characteristics used to compare products: bioequivalence checks similar rates and extents of active drug absorption between the test and reference formulations. The rate and extent are usually measured by peak plasma concentration (*C*_*max*_) and area under the plasma concentration vs. time curve (*AUC*). It is, therefore, mandatory for these to be correctly simulated by the model for both the test and reference.
2. Model recalibration with *in vitro* data and clinical data from an abbreviated BE trial provides estimates of the difference between the test and reference formulations’ critical quality attributes (CQAs), which are part of the model parameters. An alternative is to first use the abbreviated trial data for model verification. If the model needs improvement, updating it one way or another is necessary, and Bayesian recalibration using the abbreviated trial data can be tried. If the model does not need recalibration, it can be used directly to perform further simulations. Recalibration is mandatory for an empirical PK model because there is no other way to inform the difference between the test and reference.
3. Simulation of virtual trials of different sizes for BE assessment, type I and type II error, and CQA safe-space analyses using data-based methods. Those methods are usually related to the two one-sided *t*-test (TOST test) (Schuirmann, 1987) with trial design-specific adaptations. Model-integrated methods have been proposed, whereby *C*_*max*_ and *AUC* are estimated by model-fitting (Dubois et al., 2010). Statistical tests for BE control the type I error rate and the probability of declaring a formulation as bioequivalent when it is not bioequivalent, which is clearly a consumer risk (Möllenhoff et al., 2022). Type II error rate, the probability of wrongly declaring a formulation as non-bioequivalent, is clearly a producer risk but also indirectly a consumer risk. Type II error depends on the trial size and intra-group variances. The power of a trial (one minus type II error) is usually required to be at least 80% to avoid running wasteful trials for sponsors and participants. Since type II error and power strongly depend on the variability structure of *C*_*max*_ and *AUC* measures and drug concentration measurements’ uncertainty, the model should also be predictive of *C*_*max*_ and *AUC* variances.

Workflow B differs from workflow A in the last step. There is uncertainty about the difference between the test and reference because information is imperfect and all the model parameters calibrated at step 2 of the workflow, even without recalibration with *in vivo* data, have a joint posterior probability distribution, to which all components of variability and uncertainty contribute. Therefore, the average *C*_*max*_ and *AUC* differences between the test and reference have a joint posterior predictive distribution that can be estimated. With such a posterior distribution, the Bayesian strict equivalent of the current standard regulatory rule [focusing on population bioequivalence (Ghosh and Khattree, 2003)] is to declare bioequivalence if the probability that both *C*_*max*_ and *AUC* differences fall within the 0.8–1.25 interval is equal or superior to 0.95.

In workflow B, questions about type I and type II errors of statistical testing for a simulated trial disappear from our concerns. However, concerns regarding making a correct decision are still legitimate and directly related to model validation. Safe-space analyses are still possible and valid. For nonlinear PK models, the posterior predictive distribution of *C*_*max*_ and *AUC* differences can be estimated by Monte Carlo simulations.

### 2.2 Case study data: VBE of generics for long-acting injectable products

We will illustrate our workflows using a specific case study on paliperidone palmitate (PP). One of the most important problems in the management of schizophrenic patients is poor medication adherence (Valsecchi et al., 2019). LAI formulations, which can release the drug over months, improve treatment adherence. The first marketed LAI suspension of paliperidone palmitate, an antipsychotic agent (Samtani et al., 2009; Magnusson et al., 2017), called INVEGA SUSTENA^®^ or PP1M in the following, is usually injected once per month. A more recent formulation (INVEGA TRINZA^®^, PP3Mr in the following) can be injected every 3 months. Reliable population PK models have been developed and published for PP1M and PP3Mr (Samtani et al., 2009; Magnusson et al., 2017). These models were developed using clinical data collected in phase I and phase III trials. The subjects received an injection of PP1M (dose range 50–150 mg eq.) every month for 4 months; and then they switched to PP3Mr (dose range 175–525 mg eq.) with an injection every 3 months for 1 year (i.e., four injections in total). We will assess our VBE workflows with these models.

### 2.3 Models for PP long-acting injectables

**FIGURE 2:**
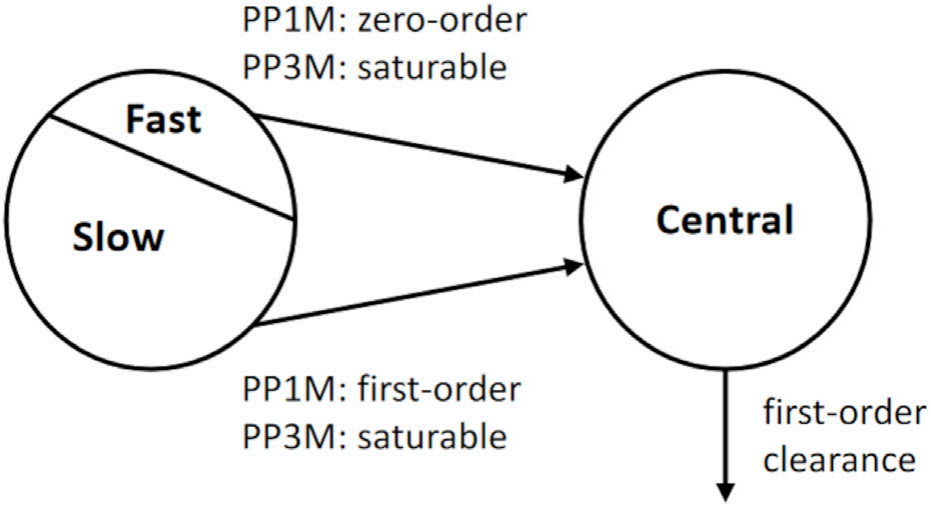
Structural part of the population PK models used for the innovator’s PP 1-month (PP1M) and 3-month (PP3Mr) long-acting injectable products (Samtani et al., 2009; Magnusson et al., 2017).

The PP1M and PP3Mr models we use are illustrated in Figure 2. The PP1M model published by Samtani et al. (2009), which was also used by Magnusson et al. (2017), is a two-compartment model with a depot and a central compartment. The depot is split into a slow and a fast depot. The PP1M model describes drug release from the depots by linear processes. The structure of the PP3Mr model (Magnusson et al., 2017) is similar but with two saturable drug-release processes (described by Hills equations) from the depots.

The two models can be jointly used to model trials, in which the starting dose is PP1M (for equilibration of the patients), followed by PP3Mr injections (Magnusson et al., 2017). The equations are solved concurrently because the PP1M depots may still release the drug after the first PP3Mr injection. This is the approach followed by Magnusson et al. (2017). The model considers the fact that some subjects had already been treated with PP before entering the trial and had an unknown quantity, *Q*_*central*_ (0), of PP in the central compartment. This quantity is, therefore, an additional model parameter. Note that this model assumes that all injections go to the same injection site, replenishing the previous depot.

For the PP1M model, after an intra-muscular injection of a *Dose*_1_ of paliperidone palmitate in the depot compartment at the *j*th injection time, *t*_*ij*_, a fraction *f*_1_ of *Dose*_1_, is available for release from the depot through a zero-order process up to time *t*_*l*1_, at which *f*_1_ × *Dose*_1_ has been released. After *t*_*l*1_, the remaining amount of *Dose*_1_ is released through a first-order process with the rate constant *k*_*a,*1_. The corresponding ordinary differential equations (Eqs. 1-3) are

$$\frac{\partial Q_{depot,1}}{\partial t}=-\frac{f_{1}\times {Dose}_{1}}{t_{l1}},\text{with }Q_{depot,1}\left(t_{ij}\right)+=f_{1}\times {Dose}_{1},\text{if }t<t_{ij}+t_{l1},$$ (1)

$$\frac{\partial Q_{depot,1}}{\partial t}=-\left(k_{a,1}{\times Q}_{depot,1}\right),\text{with }Q_{depot,1}\left(t_{ij}+t_{l1}\right)+=\left({1-f}_{1}\right)\times {Dose}_{1},\text{if }t\geq t_{ij}+t_{l1},$$ (2)

$$\frac{\partial Q_{central}}{\partial t}=-\frac{\partial Q_{depot,1}}{\partial t}-CL\times \frac{Q_{central}}{V},$$ (3)

where Q_depot,1_ and *Q*_*central*_ are the amounts of drug in the depot and central compartments, respectively; *CL* is the drug clearance from the central compartment; and *V* is the volume of that compartment.

In the PP3Mr model (Magnusson et al., 2017), two saturable release processes (rapid and slow, using Hills equations) describe drug release (Eqs. 4-6):

$$\frac{\partial Q_{depot,r,3}}{\partial t}=-\;\frac{k_{ar3,\max}\times Q_{depot,r,3}}{{k_{ar3,50}+Q}_{depot,r,3}},\text{with }Q_{depot,r,3}\left(t_{ij}\right)+=f_{3}\times {Dose}_{3},$$ (4)

$$\frac{\partial Q_{depot,s,3}}{\partial t}=-\frac{k_{as3,\max}\times {Q_{depot,s,3}}^{\gamma }}{{k_{as3,50}}^{\gamma }+{Q_{depot,s,3}}^{\gamma }},\text{with }Q_{depot,s,3}\left(t_{ij}\right)+=\left({1-f}_{3}\right)\times {Dose}_{3},$$ (5)

$$\frac{\partial Q_{central}}{\partial t}=-\frac{\partial Q_{depot,r,3}}{\partial t}-\frac{\partial Q_{depot,s,3}}{\partial t}-CL\times \frac{Q_{central}}{V},$$ (6)

where *Q*_*depot,r,3*_, *Q*_*depot,s,3*_, and *Q*_*central*_ are the respective amounts of drug in the rapid-release depot, slow-release depot, and central compartments, respectively; $k_{ar3,\max}$ , $k_{ar3,50}$ , $k_{as3,\max}$ , $k_{as3,50}$ , and *γ* are Hills drug-release and absorption parameters. *Dose*_3_ is the dose of paliperidone palmitate at the *j*th injection time; and *f*_3_ is the fraction of *Dose*_3_ going to the fast-release depot.

### 2.4 Hierarchical population model

The above structural model was developed, calibrated, and checked in a population framework with large clinical datasets of the innovator’s drug (Magnusson et al., 2017). We use the same framework.

At the subject level, plasma concentration measurements were assumed to follow a lognormal distribution with a geometric mean given by the model-predicted subject-specific central compartment concentration profile and a variance $\sigma^{2}$ in the log-space (Eq. 7). The predicted plasma concentration values at times $t_{i,j}$ were obtained using the structural model, *f*, described above:

$$C_{i,j}\;∼\;\mathrm{LN}\left(f\left(\theta_{i},t_{i,j}\right),\sigma^{2}\right).$$ (7)

For parameters $k_{a,1}$ , $k_{as3,\max}$ , $k_{as3,50}$ , $k_{ar3,50}$ , CL , and V , subject-specific parameter values $\theta_{i}$ were assumed to be lognormally distributed around population geometric means μ with variances $\Sigma^{2}$ in the log-space (Eq. 8):

$$\theta_{i}\;∼\;\mathrm{LN}\left(\mu ,\Sigma^{2}\right).$$ (8)

Parameters $k_{ar3,\max}$ and γ were assumed to be the same for all subjects. In the analyses of Samtani et al. (2009) and Magnusson et al. (2017), a multivariate normal distribution was used, although they did not report the covariance values. We assumed that they were negligible and used only the variances they provided. This adjustment appears not to affect the ability of the model to reproduce the results of Magnusson et al. (2017).

For parameters $f_{1}$ and $f_{3}$ , a logit transformation was used, and the corresponding logit, κ , was assumed to be lognormally distributed (Eqs. 9-10):

$$\kappa_{i}\;∼\;\mathrm{LN}\left(\frac{\mu }{1-\mu },\Sigma^{2}\right).$$ (9)

$$\theta_{i}=\frac{1}{1+\exp\;\left(-\kappa \right)}.$$ (10)

**FIGURE 3:**
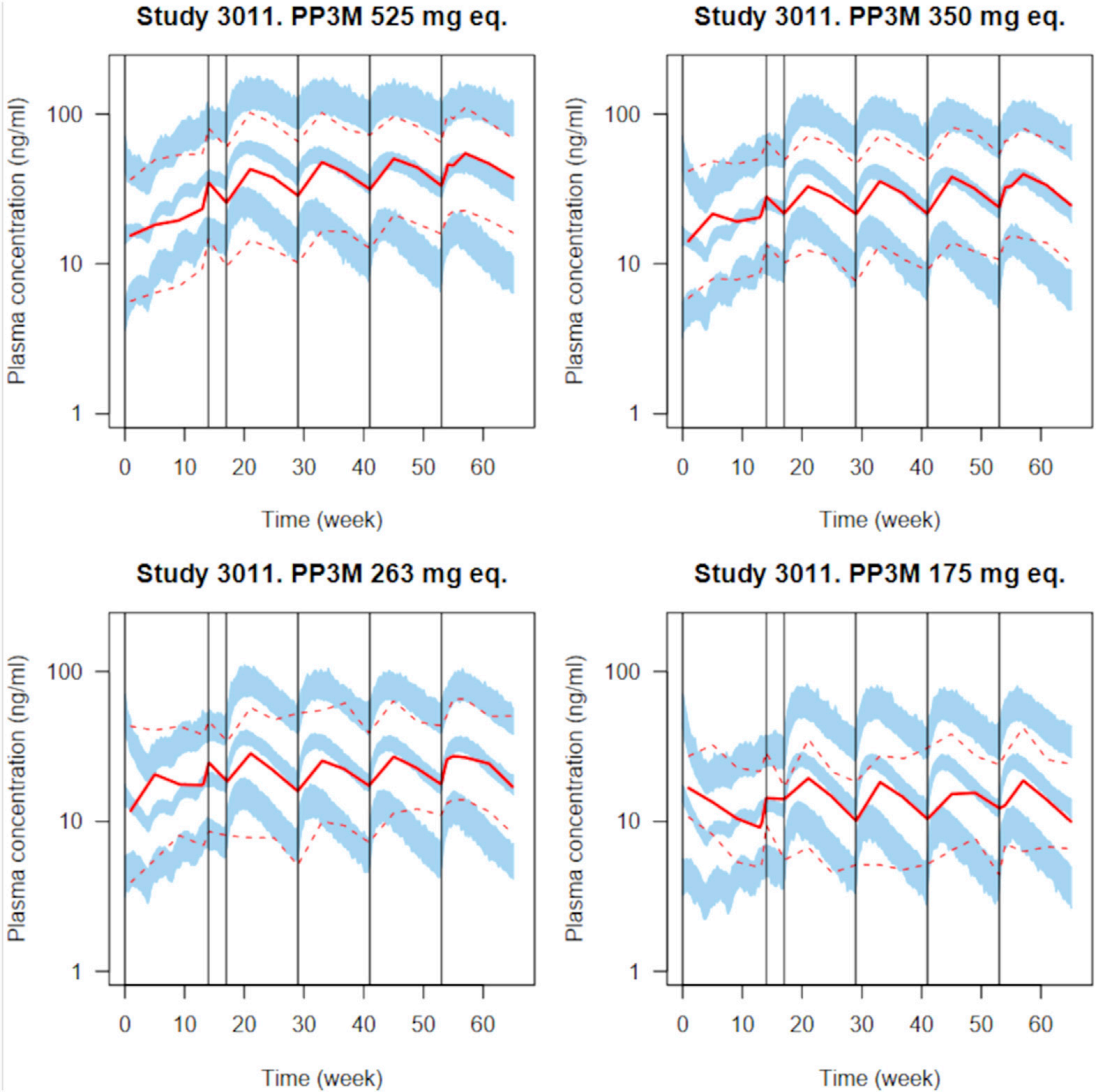
Simulated plasma PP concentrations with the PP1M and PP3Mr models for all validation plots in the original paper (Magnusson et al., 2017) for various dosages. Four injections of PP1M (dose range 50–150 mg eq.) are followed by four injections of PP3Mr (doses indicated in each panel). A total of 100 clinical trials with 130 subjects were simulated. The solid and dashed red lines represent the median and 5th and 95th percentiles of the observations, as reported in the original publication; the shaded blue areas represent the 90% confidence interval of the median and 5th and 95th percentiles predicted by our implementation of the model.

The initial quantity of PP in the central compartment, *Q*_*central*_ (0), was not reported for the subjects of the Magnusson et al. trials. We assumed that the subject-level values *Q*_*central,i*_ (0) were lognormally distributed around a population geometric mean equal to 30 mg eq. of PP, with a geometric SD of 1.5. Those values were adjusted manually by us to match the starting PP plasma concentration levels shown in Figure 3. They have a minimal impact on the concentrations during the last dosing period, which is approximately 1 year later. We also have uncertainty on the exact dose of PP1M for each subject (some unreported dose adjustment was applied to the last three doses of PP1M to reach the therapeutic window for each subject), but we left that to be a part of the residual error (and it is unclear whether this reduced subject variability or not).

To model differences between the reference (PP3Mr) and test (PP3Mt) formulations, we introduced a vector of relative changes, δ , affecting the geometric means of the six drug-release and absorption parameters of the model, which were $f_{3}$ (the fraction of PP rapidly released), $k_{as3,\max}$ (maximum release rate from the slow-release depot), $k_{ar3,\max}$ (maximum release rate from the rapid-release depot), $k_{as3,50}$ (Hills coefficient for the slow-release depot), $k_{ar3,50}$ (Hills coefficient for the rapid-release depot), and γ (Hills power), in that order. These parameters should be related to product formulation CQAs, such as drug dissolution and injection medium composition. Each mean (termed $\mu_{i,test}$ in the following equation) for the test formulation, given the reference formulation value $\mu_{i,ref}$ and the relative change $\delta_{i}$ , was computed as Eq. 11

$$\mu_{i,test}=\delta_{i}\times \mu_{i,ref},\text{with }i\in \left\{1,\ldots ,6\right\}.$$ (11)

Magnusson et al. (2017) gave estimates for all the parameters’ population geometric means and geometric variances (the latter transformed to coefficients of variation, CV, in natural space), together with precisions (as CVs) of those estimates. We used those numbers, appropriately transformed, to define prior distributions for the model’s parameters [for details, see Supplementary Material, structural model C code (v4)]. Magnusson et al. also introduced covariate measurements for their subjects, but individual covariate values were not reported in the original model (Magnusson et al., 2017). Therefore, their covariate model was not implemented in this study.

### 2.5 Recalibration of the model with simulated abbreviated parallel clinical trial data

Without an actual abbreviated trial of PP3Mr and PP3Mt formulations, we simulated an abbreviated parallel BE clinical trial with 25 subjects per arm. All the model parameters with prior uncertainty or population variability were randomly sampled to generate virtual subjects. The same dosing regimen and sampling scheme as described in Magnusson et al. (2017) were applied. In both arms, a final PP dose of 525 mg eq. was tested; the initial PP1M dose was 150 mg eq. Plasma concentrations simulated at 54, 55, 57, 59, and 63 weeks for each individual were used for model recalibration. The calibration dataset, therefore, consisted of 250 measurements from 50 individuals. Differences between PP3Mr and PP3Mt drug-release parameters were simulated at the population average level by setting the value of the second component of vector δ , i.e., $\delta_{2}$ , to 1.05 (5% difference). This increases the $k_{as3,\max}$ population mean by 5% for the above test reference. The other components of δ were set to 1.0 (no difference). Parameter $k_{as3,\max}$ was determined to be the most influential on *C*_*max*_ and (partial) *AUC* at steady state during the last dosing period; it conditions the rate of release from the slow depot compartment (see Supplementary Material section sensitivity analysis of the impact of drug-release parameters on C_max_ and AUC).

The above simulated abbreviated trial is the only source of information we considered to estimate the differences between the parameters of the test and reference formulations. Estimating those differences is important to simulate realistic final BE trials. Because our population PK model has strong prior information on the reference formulation parameters, a Bayesian approach is well suited to estimate the value of the difference $\delta_{2}$ . Furthermore, the other components of δ were set to 1.0. Metropolis–Hastings Markov-chain Monte Carlo (MCMC) sampling was used to obtain a sample of the parameter values from their joint posterior distribution given the abbreviated trial data (Gelman et al., 1996). We set the other population mean and variance parameters to their central values [maximum likelihood estimate, MLE, values as reported by Magnusson et al. (2017)] of their prior distributions. We set the residual error $\sigma^{2}$ to the MLE reported by Magnusson et al. (2017). It would not be meaningful to update these parameters on the basis of a small trial as the subjects from the reference trial arm are drawn from those priors by construction. Subject-level parameters *f*_1_, *f*_3_, k_a,1_, $k_{ar3,50}$ , $k_{as3,\max}$ , $k_{as3,50}$ , *CL*, *V*, and *Q*_*central*_ (0) were estimated jointly with the values of the difference test vs. reference parameter $\delta_{2}$ . A vague lognormal prior was assigned to $\delta_{2}$ , with a geometric mean of 1 and a geometric SD of 2 . The prior distribution of $\delta_{2}$ can be seen in Figure 5, and it approximately spans a factor of 8.

Four MCMC chains of 10,000 iterations were run, and the first 2,500 iterations were discarded. The convergence of the remaining 4 × 7,500 was checked using Gelman and Rubin diagnostics (Gelman and Rubin, 1992).

### 2.6 Large parallel virtual trial simulation, BE assessment, and type I and II error analyses

In this step, workflows A and B diverge. Workflow A is data-based. We simulated a “realistically large” virtual parallel BE trial and analyzed it as a standard BE trial. Since the trial design is parallel, a simple TOST test was used on the data-based estimates of *C*_*max*_ and partial *AUC* over the last dosing period (the *AUC* was estimated by the trapezoidal rule). A simulation-based power analysis [see Supplementary Material, section power calculations (workflow A)] indicated that 130 subjects would be adequate given all the prior information we had on PP kinetics with the reference formulation. To simulate a parallel BE trial with 130 subjects per arm, we fixed the population means and variances to the central values of their prior distributions [MLE value reported by Magnusson et al. (2017)]. Virtual subjects were sampled from their population distribution. We set the residual error $\sigma^{2}$ to its MLE (Magnusson et al., 2017). The value of $\delta_{2}$ was set to its mean estimate after calibration with the abbreviated trial data. The other δ values were set to 1. The dosing schedule and sampling times were the same as in the above trials.

Workflow B uses Monte Carlo simulations to obtain an estimate of the joint posterior predictive distribution of the ratios $\delta_{Cmax}$ and $\delta_{AUC}$ between the population geometric means of *C*_*max*_ (and *AUC*, respectively) for the test and the reference formulations. To be fully model-integrated, we estimated *C*_*max*_ by computing PP plasma concentration at 100 time points during the last dosing period (the system is at steady-state, and we checked that using 100 points was largely enough to obtain a stable estimate of *C*_*max*_); *AUC* was computed by numerical integration (adding one ODE to the system of ODEs to solve) over the same period. We formed the $\delta_{Cmax}$ and $\delta_{AUC}$ ratios for 1,000 simulated trials with 1,000 subjects per arm each. Each trial was simulated exactly as the large trial described above, except for the number of subjects.

### 2.7 Safe-space calculations

For workflow A, 1,000 virtual trials with 130 subjects per arm were run as above, except for sampling each element of *δ* from a uniform [0.5, 2] distribution. This generated a large number of bioequivalent and non-bioequivalent test formulations. We computed the geometric mean ratios test over the reference for *C*_*max*_ and *AUC* in those simulated trials. BE for *C*_*max*_ and *AUC* of each trial was assessed with a TOST test. BE was declared if it passed for both *C*_*max*_ and *AUC*. Color-coded TOST BE passes and fails were plotted against the values sampled for the different components of the vector δ .

For workflow B, safe-space calculations required computing the posterior predictive distribution of $\delta_{Cmax}$ and $\delta_{AUC}$ over the whole drug-release parameters’ space. However, we know that the safe-space limits should be crisp (because they are not blurred by uncertainty induced by a limited trial size), and the workflow A safe-space calculations gave us a rough estimate of the safe-space shape. We, therefore, focused on determining the boundaries of the safe-space for the two most critical absorption parameters ( $f_{3}$ and $k_{as3,\max}$ ) of the PK model. For each trial point (near the boundary) of the $f_{3}$ and $k_{as3,\max}$ space, we based our decision for BE on the $\delta_{Cmax}$ and $\delta_{AUC}$ ratios from 1,000 simulated trials with 1,000 subjects per arm each. Each trial was simulated exactly as the large trial described above, except for the number of subjects.

### 2.8 Software

We coded the structural PK model as a C-language routine callable from workflow *R* (R Development Core Team, 2013) scripts using the Nimble *R* package (de Valpine et al., 2017; NIMBLE Development Team, 2022) to perform Monte Carlo simulations, Bayesian inference, and posterior analyses. The corresponding codes are given in Supplementary Material (section computer codes.

## 3 Results

### 3.1 Prior model checking

To check our PM model implementation, we compared the simulations obtained with it to the measured paliperidone concentrations reported by Magnusson et al. (2017). Figure 3 presents the simulated paliperidone plasma concentration measurement percentiles overlaid with the reported data summaries for several PP1M and PP3Mr dosages. A total of 100 clinical trials with 130 subjects were simulated to integrate uncertainty in population parameter values, inter-individual variability, and residual error. The median and 5th and 95th percentiles of the plasma concentrations for the 130 subjects were computed in each trial. The blue bands in Figure 3 are bounded by the 5th and 95th percentiles of the distributions of those three quantiles over the 100 trials. Despite missing information on the subjects’ covariates, our implementation of the model captures the reported PP1M and PP3Mr kinetics well, including inter-individual variability and residual error. Summary ratios of the predicted over observed PP3Mr median concentration values do not exceed 1.25. The code used for generating that plot (population PK model implementation in Nimble R v8_pop) and further details are given in Supplementary Material, in the section prior to model checking.

### 3.2 Abbreviated clinical trial simulation

**FIGURE 4:**
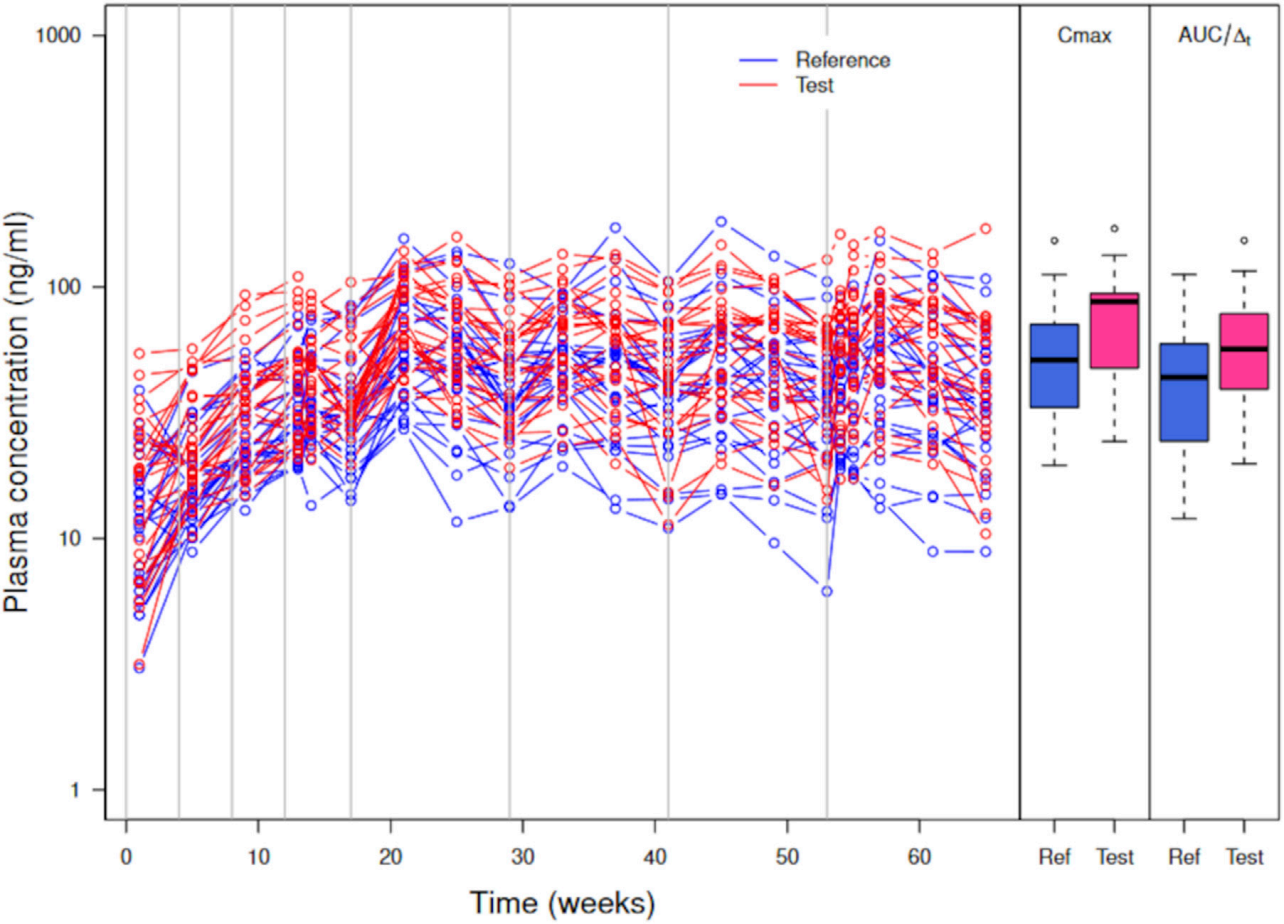
Simulated plasma PP concentrations, *C*_*max*_, and *AUC*/ $\Delta_{t}$ for 25 subjects per arm in a parallel abbreviated virtual trial when parameter $k_{as3,\max}$ was increased by 5% from the value of the reference formulation. The subjects received four injections of PP1M (150 mg eq.) prior to four injections (525 mg eq.) of PP3Mr (blue) or PP3Mt (red). The gray lines indicate the injection times.

Figure 4 shows the simulated concentration data, *C*_*max*_, and *AUC*/ $\Delta_{t}$ for the simulated abbreviated clinical trial. *C*_*max*_ and *AUC* were computed for the last PP3Mr or PP3Mt dosing period, in which plasma concentrations were sampled at weeks 54, 55, 57, 61, and 65, and $\Delta_{t}$ is the corresponding time span (11 weeks). *AUC*/ $\Delta_{t}$ is an average concentration and can be plotted on the same scale (see also in Supplementary Material, section abbreviated clinical trial simulation summary plot).

*C*_*max*_ and *AUC* are increased on average in the test formulation (geometric mean ratio test/reference at 1.38 for both). Such an increase is large compared to a couple of percentages expected with a 5% increase in $k_{as3,\max}$ (see in Supplementary Material, section sensitivity analysis of the impact of drug-release parameters on C_max_ and AUC). A standard TOST test on either *C*_*max*_ or *AUC* did not allow concluding bioequivalence due to the size of the difference between formulations, a low number of subjects, and large inter-individual variability in *C*_*max*_ and *AUC* (51% and 54%, respectively).

### 3.3 Recalibration of the model with the simulated abbreviated clinical trial data

**FIGURE 5:**
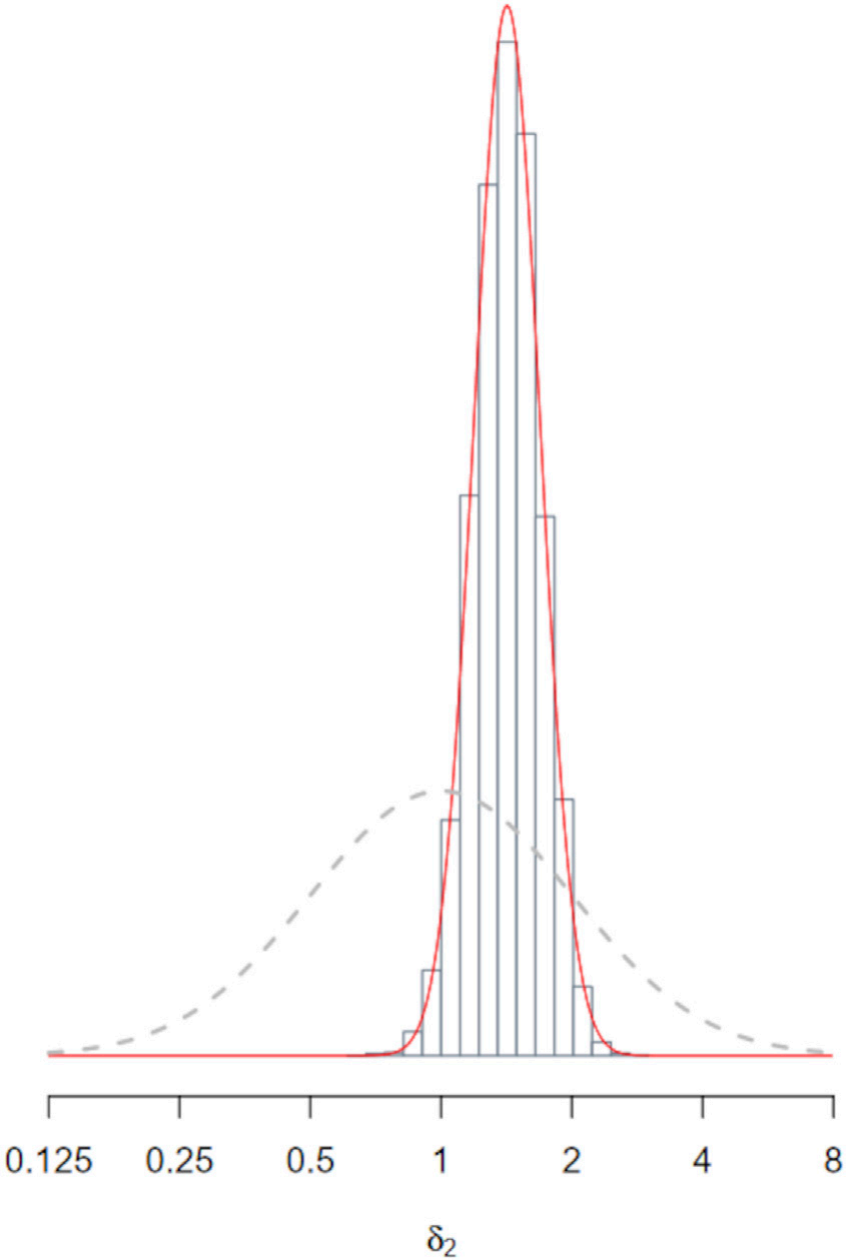
Posterior distribution of $\delta_{2}$ (histogram and smooth density curve). The dotted line shows its prior distribution. The posterior is much more precise.

MCMC sampling was used to estimate the joint posterior distribution of $\delta_{2}$ and of the subject-specific parameters *f*_1,*i*_, *f*_3,*i*_, *k*_*a,*1,*i*_, $k_{ar3,50,i}$ , $k_{as3,\max,i}$ , $k_{as3,50,i}$ , *CL*_*i*_, *V*_*i*_, and *Q*_*central*,*i*_ (0) for the two groups of the abbreviated clinical trial (451 parameters altogether). Subject-specific parameters can be considered nuisance parameters that were integrated. Sufficient convergence was achieved for all parameters (see Supplementary Material for a convergence plot and a histogram of all $\hat{R}$ values in the section convergence of the model recalibration by MCMC sampling). The posterior distribution of $\delta_{2}$ , the relative difference between the test and reference maximum release rates from the drug slow depot, which should alone explain the difference between the test and reference PK profiles, had a geometric mean of 1.42, a geometric SD of 1.19, and a 95% credibility interval of 1.0–1.97 (see Supplementary Material, section posterior distribution summary for parameter *δ*_2_). Figure 5 shows the empirical posterior distribution (well approximated by a normal distribution) together with its prior. The posterior mean of $\delta_{2}$ is higher than 1.05 used for simulating the clinical data because we used a random abbreviated trial for recalibration, in which the test subjects behaved quite differently from the reference subjects. Only a much larger trial would be likely to yield more accurate estimates of the differences between the test and reference. Note that this is conservative from a consumer safety point of view but not from a production point of view.

**FIGURE 6:**
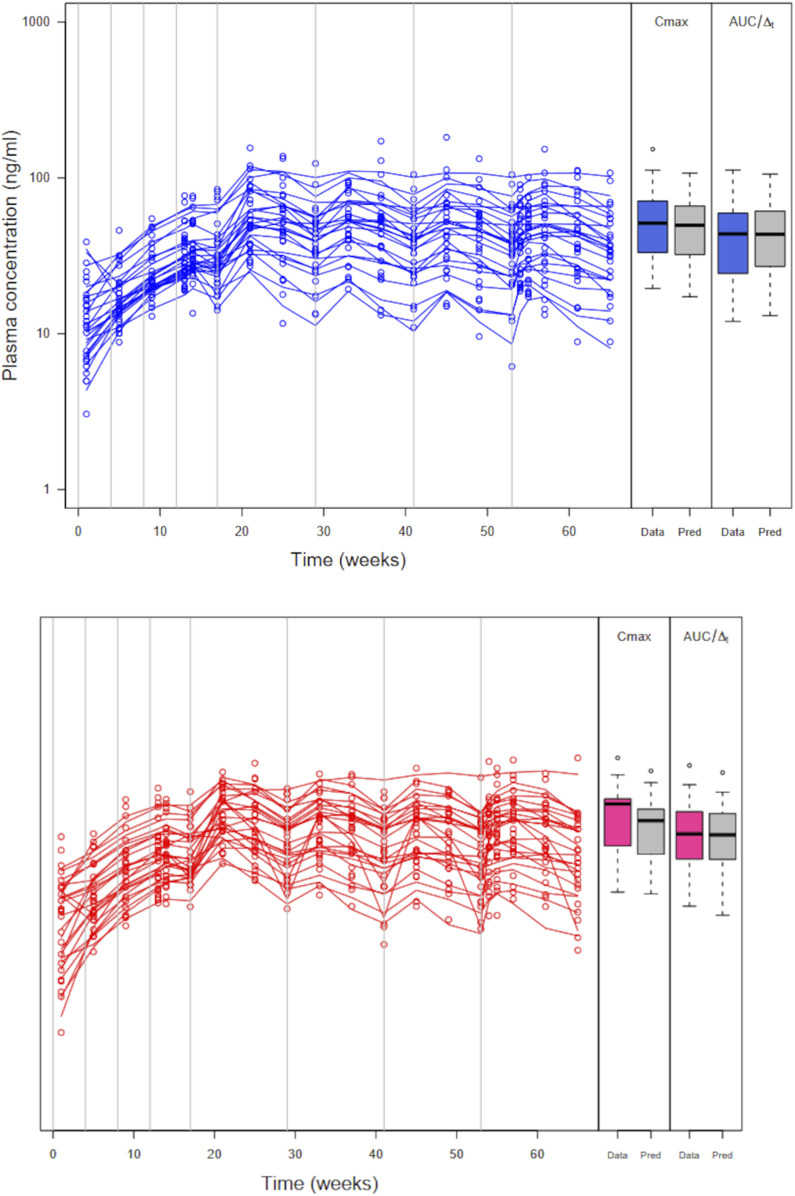
Abbreviated trial data (circles) and posterior model predicted profiles (lines) obtained with the maximum posterior population PK parameter values for the reference formulation (left panel) and the test formulation (right panel). Boxplots of *C*_*max*_ and *AUC*/ $\Delta_{t}$ are shown for the data (blue or red) and the model predictions (gray). *C*_*max*_ and *AUC*/ $\Delta_{t}$ are noticeably higher for the test formulation.

The posterior fit of the recalibrated model to the abbreviated trial data is very good, as shown in Figure 6 (see also in Supplementary Material section observations vs. predictions plot for the recalibration step). We will, therefore, assume in the following that the model is validated for both the reference and test formulations.

### 3.4 Large virtual trial simulation, BE assessment, and type I and II error analyses

#### 3.4.1 Partly Bayesian data-based workflow A

**FIGURE 7:**
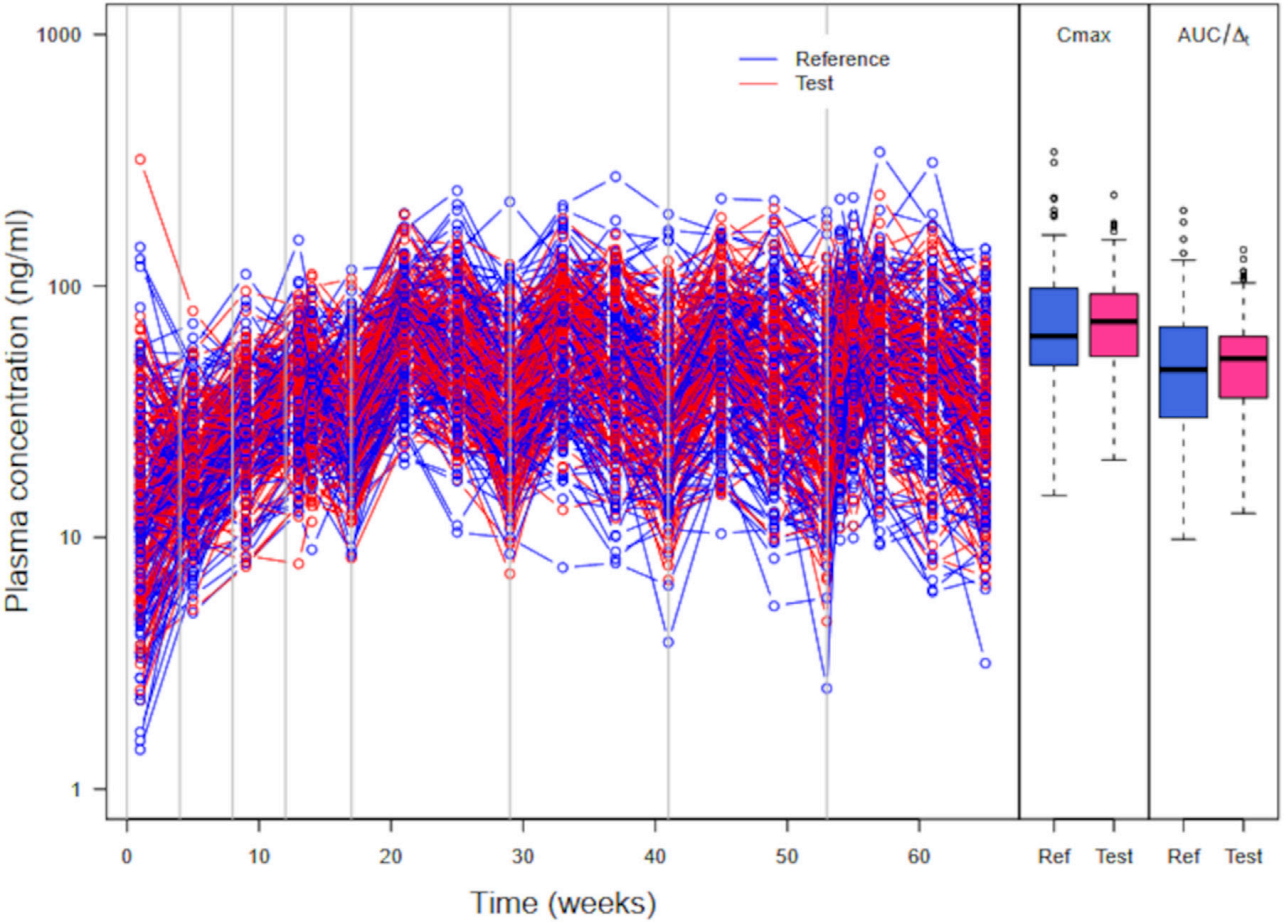
Simulated plasma PP concentrations, *C*_*max*_, and *AUC*/ $\Delta_{t}$ for 130 subjects per arm in a parallel virtual trial. The subjects received four injections of PP1M (150 mg eq.) prior to four injections (525 mg eq.) of PP3Mr (blue) or PP3Mt (red).

A plot of simulated large parallel trial plasma concentration data with *C*_*max*_ and *AUC*/ $\Delta_{t}$ is shown in Figure 7 (see also in Supplementary Material, section large virtual trial simulation summary plots). In this trial, *C*_*max*_ and *AUC* are increased on average in the test formulation (geometric mean ratio test/reference at 1.08 and 1.06, respectively). The difference between $k_{as3,\max}$ population means in the test and reference formulations, $\delta_{2}$ , was set to 1.42. A standard TOST test on either *C*_*max*_ or *AUC* concludes bioequivalence despite the large inter-individual variability in *C*_*max*_ and *AUC* (57% and 54%, respectively). This is due to the expected randomness of the virtual trial. That randomness impacts type I and type II errors (and, therefore, power) of the analysis (see Supplementary Material, sections power calculations (workflow A) and type I error analysis (workflow A)). Power can be good for a data-based approach in the case of perfect BE (also with good prior information and study designs more sophisticated than just parallel), but it degrades rapidly if sizeable, unanticipated differences exist between formulations (even though they are bioequivalent). Type I error was very low and below the expected 2.5% at each side of the BE interval. This is a side-effect of the low power of parallel BE trials; close to the BE boundaries, no trial will conclude BE due to inter-subject variability.

#### 3.4.2 Fully Bayesian model-integrated workflow B

**FIGURE 8:**
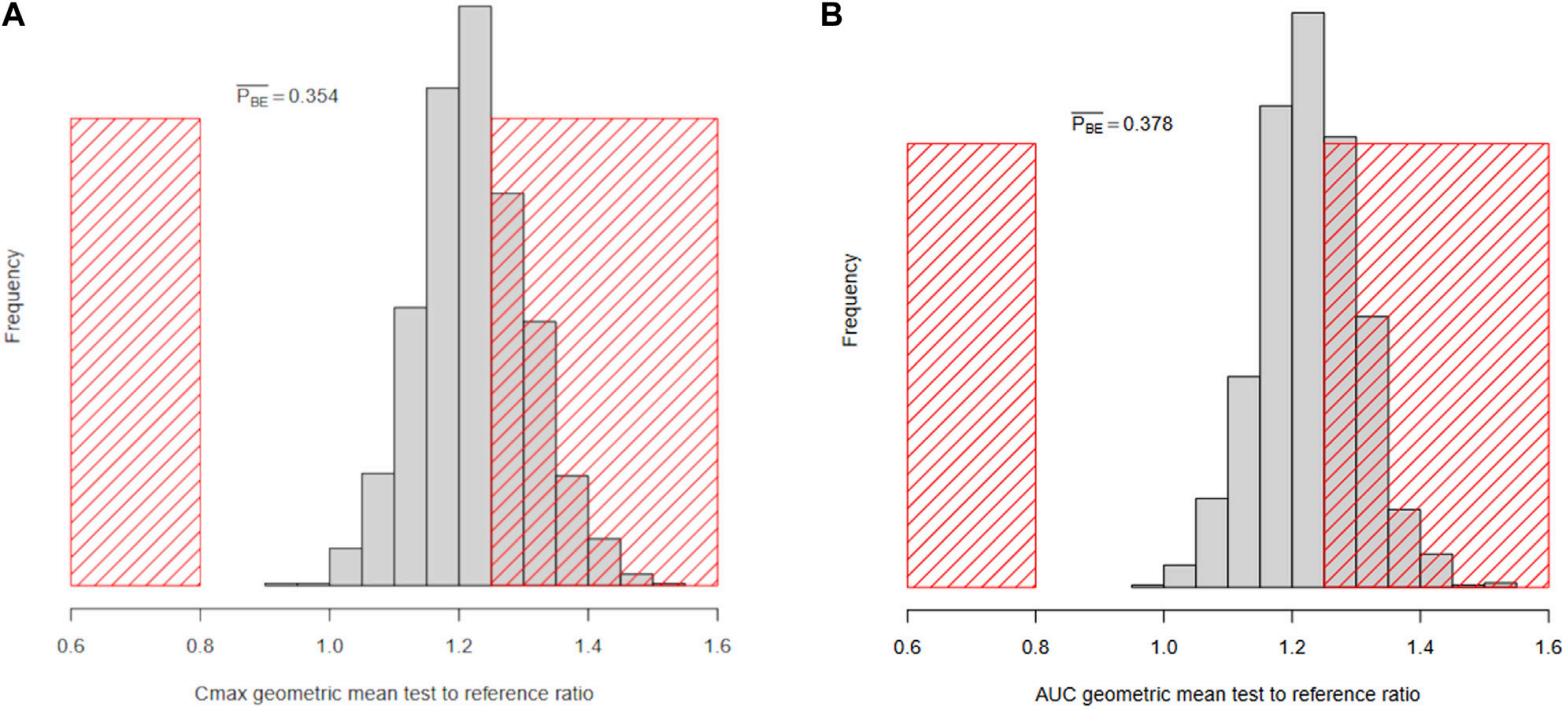
Histograms of the Bayesian marginal posterior predictive distributions of $\delta_{Cmax}$ **(A)** and $\delta_{AUC}$ **(B)** ratios. $\bar{P_{BE}}$ is the probability of non-bioequivalence. The red-shaded areas mark the standard non-bioequivalence regions (outside 0.8–1.25).

Workflow B uses Monte Carlo simulations to approximate $\delta_{Cmax}$ and $\delta_{AUC}$ ratio posterior predictive distributions (see Figure 8). The decision about BE is immediate: the *C*_*max*_ ratio exceeds 1.25 with a probability of 0.354 and the *AUC* ratio exceeds it with a probability of 0.378, so BE should not be declared. The decision about BE differs from the one reached in the data-based workflow because the latter relied on only one (albeit large) VBE trial, while in this study, we “average” the decision over 1,000 trials.

### 3.5 Safe-space analysis

**FIGURE 9:**
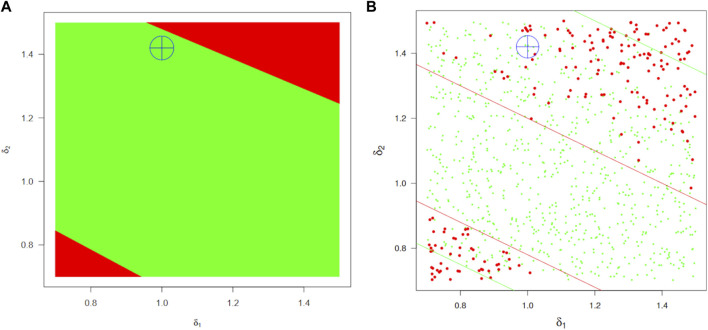
BE safe-space regions for the absorption parameters $f_{3}$ and $k_{as3,\max}$ of the PP population PK model. **(A)** Data-based estimate; the green dots indicate parallel PP trials (1,000 trials, 500 subjects per arm), for which BE was declared using the TOST test; the red dots indicate failing trials; the red lines mark “sure” safe-space; the green lines mark the limits of surely non-BE space. The blue cross marks the location of the simulated full parallel trial we simulated for BE assessment. The intermediate areas stems from imperfect statistical power. **(B)** Fully Bayesian model-based regions are much crisper.

For workflow A, Figure 9A shows the safe-space of model parameters $f_{3}$ and $k_{as3,\max}$ (proxies for CQAs), as assessed by large virtual parallel trials. Parameters $f_{3}$ and $k_{as3,\max}$ were the most influential parameters on safe-space definition, and they interact, which is why we show the results in two dimensions (results for all six drug-release model parameters are given in Supplementary Material, section full safe-space calculations for the data-based parallel trial workflow). Given the low power of the TOST test near the BE limits, the safe-space region limits are fuzzy, and the “safest” space is quite reduced. The safe region is banded due to the structure of the PP kinetic model. The location of the full parallel trial we simulated for BE assessment is given by a blue cross. It falls in that region where decisions can be inconsistent.

The safe-space identified by workflow B is shown in Figure 9B. Since power is not a problem in that framework, the region is much better defined and approximately twice as large but still coherent with the previous estimate (actually intermediate between the optimistic and pessimistic estimates marked by green and red lines, respectively, in the left panel). A ( $\delta_{1}$ , $\delta_{2}$ ) pair with a value of (1, 1.42) is a pass in this framework because, on average, it will not lead to an exceedance of the BE limits for *C*_*max*_ nor *AUC*.

In either case, those are predictive simulations that ignore uncertainty about $\delta_{1}$ and $\delta_{2}$ . It would be prudent to account for the potential uncertainty. If we have a measure of that uncertainty, then a ball of the size of the 95% posterior probability interval on δ would surround every point of the BE frontier, creating a zone of uncertainty on each side of it.

## 4 Discussion

We have presented two Bayesian virtual comparative clinical trial workflows. We demonstrated them with a realistic case study using an empirical population pharmacokinetic model of paliperidone palmitate LAI formulations. This work is not intended to be a VBE assessment for a particular product but rather a discussion of overarching issues in VBE.

PP long-acting injectable formulations are difficult to compare because inter-subject variability is high, and actual comparative trials seem unfeasible at reasonable costs and in a reasonable time. However, the pharmacokinetics of the innovator formulation are well documented, and a population PK model validated with clinical data on that formulation is available; the model describes the data well and was accepted by the US FDA (2014). Our implementation made a few necessary approximations that should not affect our conclusions: we were unable to account for unreported covariate measurements, lacked information on subject-level parameters’ covariance, and faced uncertainty regarding the exact PP dose per subject and previous treatments at the start of the validation trials. This explains why our predicted population variability is slightly higher than the published variability. Inter-occasion variability and modeling error are folded into residual error, but that should have minimal impact on our results because we simulated parallel clinical trials. Overall, the predictions were within factor 2 of the summary observations, and the median estimates were within 25% of their observed counterparts, as reported by Magnusson et al. (2017). A refined model could assume that different formulations have different variabilities in release and absorption. They might be estimable from prior clinical data and abbreviated trials with a Bayesian population PBPK approach. An alternative would be to assume the possibility of different variances and assess their impact through sensitivity analysis.

We did not use *in vitro* evidence about test and reference differences because this has already been demonstrated (Hsieh et al., 2021), and the model we used has no parameter measurable *in vitro*. Mechanistic PBPK models can better integrate prior information and data from *in vitro* experiments. However, we do not have such a model for PP LAI suspensions, and a simpler model allows us to focus on the actual differences between workflows A and B. We definitely used informative priors, but they simply summarize the data that were obtained and published for the innovator drug; in a way, they are interim estimates in a two-stage estimation process. Using weaker priors is always possible, but in that case, the model might not be validated (because it would run the risk of over-estimating uncertainties and variances) and would not be usable.

Both workflows start with a definition of prior distributions and their Bayesian recalibration with available data from an abbreviated trial. Without an actual abbreviated trial, we simulated data with a published population model. It is important to note that in a real application, these data would be observed and not simulated. Therefore, the validation step would be much more difficult than in our example, where we know the actual difference between the test and reference and can focus on the “right” model. However, that would be true for any VBE assessment and applies to both workflows A and B. Note also that the recalibration step is not needed if the prior data already inform the model sufficiently to make it valid for prediction purposes. The data-based workflow A then assesses a simulated VBE trial with a frequentist test as if it were real. The particular abbreviated trial used impacts both workflows; the particular large trial simulated impacts only workflow A. Despite using an abbreviated trial, which, by chance, over-estimates formulation differences, workflow A declares BE, but again, it is by chance. The TOST test ignores most of the prior information gathered *in vitro* and *in vivo* and judges BE on only a noisy sample of simulated concentrations. Safe-space analysis shows that the simulated large trial falls in the area where BE decisions are random because of low power. This poses the problem of how to define “large” (and still “realistic”) for a virtual trial. Performing a standard statistical analysis of only one large trial is also problematic because the decision hinges entirely on one trial realization. Averaging over many very large trials could be done, but overall, assessing VBE on the basis of a large simulated trial blurs the information already obtained up to the abbreviated trial stage. That is because a large simulated trial does not bring new information, and the subsequent statistical test adds unjustified randomness to the decision process.

The model-integrated workflow B is more coherent and bases decisions on the expected future rather than on a particular virtual trial simulation. The posterior distribution of formulation differences is used to calculate posterior predictive distributions of PK measures of drug absorption. Those directly give us the probability that formulation differences will lead to unacceptable differences in drug absorption (see Figure 8). The VBE assessment then simply estimates the probability that *C*_*max*_ or *AUC* differences exceed predefined limits. This is essentially equivalent to the current decision rule, with a probability estimated more accurately. The decision depends on the uncertainty regarding the size of formulation differences, which is affected by the inter-subject variability in measures of rate and extent of drug absorption (in particular in a parallel trial design). We used model-based estimates of *C*_*max*_ and *AUC* because it would not make sense to re-introduce measurement error in the process when it has already been accounted for during model calibration. Overall, workflow B controls consumer risk strictly while minimizing producer risk. The Bayesian decision rule also rewards data gathering in the first steps of the workflow. Note that the abbreviated trial could have had any design (the more informative, the better, so a cross-over design could be used). The design of the abbreviated trial should be closely examined, and the use of other metrics of rate and extent (e.g., *C*_*min*_ and various forms of partial AUC) could be investigated (Lionberger et al., 2012; Gajjar et al., 2022).

Concerns about making the right decision with an acceptable error rate do not disappear in workflow B. However, standard statistical test performances (e.g., type I and II error assessments) do not apply anymore because there is no need for a large trial and the associated statistical testing. In our case study, if we declare bioequivalence and release the drug into the market, there is a 35% chance that we will release a non-bioequivalent product; if we do not declare bioequivalence and block the product, there is a 65% chance that the product is bioequivalent in the population. So, it is a judgment call. However, if we adhere to the strict practice of controlling direct consumer risk at 5%, we would reject bioequivalence with a relatively high direct producer risk. A deeper problem is that a VBE framework, either data-based or model-integrated, has very little specific clinical evidence (only an abbreviated trial) available. However, it benefits from using a validated (i.e., as good as possible) structural model, *in vitro* data, and published prior information (which can be massive in the case of PBPK models). Therefore, model structure and correct parameterization are very important for both workflows, and model verification is of paramount importance in VBE. Modeling errors can be introduced and could have more impact than in a BE assessment (Tsakalozou et al., 2021). Standard BE trial analyses also make assumptions (like when using drug plasma concentrations for assessing the local bioequivalence of a drug targeting the gastro-intestinal tract), but the issue is more glaring in VBE assessment. A further complication is that we simulate the abbreviated trial, and the “ground truth” of our case study is laid bare for everyone to compare the results of workflows A and B. Readers can immediately see the incoherence between “truth” and “decision”: the data-based workflow leads to a correct decision if we know the truth, but it leads to an incorrect decision given the information from the abbreviated trial. On the contrary, the model-integrated workflow decision is correct, given the abbreviated trial, but it is incorrect given the ground truth. In a “real-life VBE assessment,” we would only have a model, its prior parameter distributions, *in vitro* data, and data from one abbreviated clinical trial. The ground truth would not be accessible to us, and workflow A would always be at the mercy of incoherent abbreviated trial and large trial simulations. However, we show that workflow B is more coherent and safer for everyone (producers and consumers). In a way, in a data-based VBE framework, type I and type II calculations on the virtual large trial can be a smoke screen, giving a false sense of security as if they were dispelling the only source of potential error while masking the real crux of VBE: having a correct model. So, we should not conduct VBE assessments in the same way as BE assessments and should not judge a VBE assessment in the same way as a BE assessment.

Safe-space analyses average over many simulations and are not affected by a specific trial simulation. However, they still differ between the two workflows, and this may be viewed as our most important contribution. Safe-space calculations are more precise with workflow B because the producer risk is minimized. Those calculations for workflow B took longer (12 h on an 8-core laptop machine) than for workflow A. Preliminary calculations with workflow A could orient the search for precise safe-space boundaries in a fully Bayesian framework, as shown in this study.

Overall, we have shown that a Bayesian framework is well-suited for VBE assessment. We believe that virtual comparative trials would generally benefit from the transparency and improved accuracy they provide. We need to gain more experience with it, in particular in real-life case studies with mechanistic models such as PBPK models.

## Data Availability

The simulated data we generated can be reproduced using the computer scripts given in Supplementary Material. Further requests can be directed to the corresponding author.
