# Supplementary material for: A Bayesian framework for virtual comparative trials and bioequivalence assessments
Source: Front Pharmacol. 2024 Jul 30;15:1404619. doi: 10.3389/fphar.2024.1404619 (PMC11319711; doi:10.3389/fphar.2024.1404619)
Supplement: Supplementary file 1 [file DataSheet1.PDF]

Supplementary Material
to
A Bayesian Framework for Virtual Comparative Trials and Bioequivalence Assessment

Frederic Y. Bois, Celine Brochot

Certara UK Limited, Simcyp Division, Sheffield, UK.
[celine.brochot, frederic.bois]@certara.com

Contents

Paliperidone palmitate long-acting injectable PK models 2
Hierarchical population model 3
Prior model checking 4
Sensitivity analysis of impact of drug-release parameters on C_{max} and AUC 7
Power Calculations (Data-based Workflow A) 7
  Methods 7
  Results 8
Type I Error Analysis (Data-based Workflow A) 9
  Methods 9
  Results 10
Abbreviated clinical trial simulation summary plot 11
Convergence of the model recalibration by MCMC sampling 12
Posterior distribution summary for parameter δ_2 13
Observations *vs*. predictions plot for the recalibration step 13
Large virtual trial trial simulation summary plot 14
Full safe-space calculations for the data-based workflow 15
Computer codes 15
  Structural model C code (v4) 15
  Population PK model implementation in Nimble R (v8_pop) 16
  Partly Bayesian workflow implementation in R (v4) 23
  Statistical model in R (v13) 35
  TOST code in R 39
  C_{max} and AUC data-based calculations code in R 39
  Structural model C code (v5) 40
  Statistical model in R (v16) 41
  Fully Bayesian workflow in R (v16) 45
  Fully Bayesian safe-space calculations in R 48
References 51

1 Paliperidone palmitate long-acting injectable PK models

The PP1M model published by Samtani *et al.* [1], also used by Magnusson *et al.* [2], is a two-compartment model with a depot and a central compartment. The structure of the PP3Mr model [2] is similar, but with two saturable release processes (described by Hills equations) from the depot compartment.

The two models were jointly used to model trials in which the starting dose is PP1M (for equilibration of the patients) followed by PP3Mr injections [2]. The equations are solved concurrently because PP1M depot may still release drug after the first PP3Mr injection. This is the approach taken by Magnusson *et al.* [2]. The model considers the fact that some subjects had already been treated with PP before entering the trial and had an unknown quantity, $Q_{central}(0)$, of PP in the central compartment. This quantity is therefore an additional model parameter. Note that this model assumes that all injections go to the same injection site, replenishing the previous depot.

For the PP1M model, after an intra-muscular injection of a $Dose_1$ of paliperidone palmitate in the depot compartment at the *j*-th injection time, t_{ij}, a fraction f_1 of $Dose_1$ is available for release from the depot through a zero-order process up to time t_{l1}, at which $f_1 \times Dose_1$ has been released. After t_{l1}, the remaining of $Dose_1$ is released through a first order process with rate constant $k_{a,1}$. The corresponding ordinary differential equations are:

$$\frac{\partial Q_{depot,1}}{\partial t} = -\frac{f_1 \times Dose_1}{t_{l1}}, \text{ with } Q_{depot,1}(t_{ij}) \mathrel{+}= f_1 \times Dose_1, \text{if } t < t_{ij} + t_{l1} \quad (1)$$

$$\frac{\partial Q_{depot,1}}{\partial t} = -(k_{a,1} \times Q_{depot,1}), \text{ with } Q_{depot,1}(t_{ij} + t_{l1}) \mathrel{+}= (1 - f_1) \times Dose_1, \text{if } t \geq t_{ij} + t_{l1} \quad (2)$$

$$\frac{\partial Q_{central}}{\partial t} = -\frac{\partial Q_{depot,1}}{\partial t} - CL \times \frac{Q_{central}}{V} \quad (3)$$

where $Q_{depot,1}$ and $Q_{central}$ are the amounts of drug in the depot and central compartments, *CL* is the drug clearance from the central compartment and *V* is the volume of that compartment.

The structure of the PP3Mr model [2] is similar, but with two saturable release processes (rapid and slow, described by Hills equations) from the depot compartment.

$$\frac{\partial Q_{depot,r,3}}{\partial t} = -\frac{k_{ar3,max} \times Q_{depot,r,3}}{k_{ar3,50} + Q_{depot,r,3}}, \text{ with } Q_{depot,r,3}(t_{ij}) \mathrel{+}= f_3 \times Dose_3 \quad (4)$$

$$\frac{\partial Q_{depot,s,3}}{\partial t} = -\frac{k_{as\ ,max} \times Q_{depot,s,3}{}^{\gamma}}{k_{as3,50}{}^{\gamma} + Q_{depot,s,3}{}^{\gamma}}, \text{ with } Q_{depot,s,3}(t_{ij}) \mathrel{+}= (1 - f_3) \times Dose_3 \quad (5)$$

$$\frac{\partial Q_{central}}{\partial t} = -\frac{\partial Q_{depot,r,3}}{\partial t} - \frac{\partial Q_{depot,s,3}}{\partial t} - CL \times \frac{Q_{central}}{V} \quad (6)$$

where $Q_{depot,r,3}$, $Q_{depot,s,3}$ and $Q_{central}$ are the respective amounts of drug in the rapid-release depot, slow-release depot, and central compartments; $k_{ar3,max}$, $k_{ar\ ,50}$, $k_{as3,max}$, $k_{as\ ,50}$, and γ are Hills drug-release and absorption parameters. $Dose_3$ is the dose of paliperidone palmitate at the *j*-th injection time; f_3 is the fraction of $Dose_3$ going to the fast release depot.

2 Hierarchical population model

The above structural model was developed, calibrated and checked in a population framework with large clinical datasets of the innovator's drug [2]. We use the same framework.

At the subject level, plasma concentration measurements were assumed to be lognormally distributed with a geometric mean given by the model-predicted subject-specific central compartment concentration profile and a variance σ^2 in log-space. Predicted plasma concentration values at times $t_{i,j}$ were obtained using the structural model, *f*, described above:

$$C_{i,j} \sim LN\left(f\left(\theta_i, t_{i,j}\right), \sigma^2\right) \tag{7}$$

For parameters $k_{a,1}$, $k_{as3,max}$, $k_{as3,50}$, $k_{ar3,50}$, CL, and V, subject-specific parameter values $\boldsymbol{\theta}_i$ were assumed to be lognormally distributed around population geometric means $\boldsymbol{\mu}$ with variances Σ^2 in log-space:

$$\theta_i \sim LN(\mu, \Sigma^2) \tag{8}$$

Parameters $k_{ar3,max}$ and γ were assumed to be the same for all subjects. In the analyses of Samtani *et al.* and Magnusson *et al.*, a multivariate normal distribution was used, but they did not report the covariances' values. We assumed that they were negligible and use only the variances they provided. This does not seem to affect the ability of the model to reproduce the results of Magnusson *et al.* (see main text).

For parameters f_1 and f_3, a logit transformation was used and the corresponding logit, κ, was assumed to be lognormally distributed:

$$\kappa_i \sim LN\left(\frac{\mu}{1-\mu}, \Sigma^2\right) \tag{9}$$

$$\theta_i = \frac{1}{1+\exp\left(-\kappa\right)} \tag{10}$$

The initial quantity of PP in the central compartment, $Q_{central}(0)$, was not reported for the subjects of the Magnusson *et al.* trials. We assumed that subject-level values $Q_{central,i}(0)$ were lognormally distributed around a population geometric mean equal to 30 mg eq. of PP, with geometric SD 1.5. Those values were adjusted manually by us to match the starting PP plasma concentration levels shown in Figure 3 of the main text. They have a very small impact on the concentrations during the last dosing period, about one year later. We also have uncertainty on the exact dose of PP1M for each subject (some unreported dose adjustment was applied to the last three doses of PP1M to reach the therapeutic window for each subject), but we left that to be part of residual error (and it is unclear whether this reduced subject variability or not).

To model differences between the reference (PP3Mr) and test (PP3Mt) formulations, we introduced a vector of relative changes, δ, affecting the geometric means of the six drug-release and absorption parameters of the model, f_3 (the fraction of PP rapidly released), $k_{as3,max}$ (maximum release rate from the slow depot), $k_{ar3,max}$ (maximum release rate from the rapid depot), $k_{as\ ,50}$ (Hills coefficient for the slow-release depot), $k_{ar3,50}$ (Hills coefficient for the rapid depot), and γ (Hills power), in that order. Those parameters should be related to product formulation CQA's such as drug

dissolution, injection medium composition, *etc*. Each mean (termed $\mu_{i,test}$ in the following equation) for the test formulation, given the reference formulation value $\mu_{i,ref}$ and the relative change δ_i, was computed as:

$$\mu_{i,test} = \delta_i \times \mu_{i,ref}, \text{with } i \in \{1, \dots, 6\} \quad (11)$$

Magnusson *et al*. [2] gave estimates for all parameters' population geometric means and geometric variances (the latter transformed to coefficients of variation, CV, in natural space), together with precisions (as CVs) of those estimates. We used those numbers, appropriately transformed, to define prior distributions for the model's parameters (for details, see Structural model C code (v4)). Magnusson *et al*. also introduced covariate measurements made on their subjects, but individual covariates values were not reported in the original model [2]. Therefore, their covariate model was not implemented here.

3 Prior model checking

Model checking consisted in comparing the simulations obtained with our model to the measured paliperidone concentrations of reported in the Magnusson *et al*. [2]. In the latter, individual data were not reported, but the median, 5th and 95th percentiles of the observations are given in their Figure 6. We digitized those plots with the Engauge Digitizer®. The model was run to ensure that the implementation of the code and of the dosing schedule were adequate.

The dose regimen simulated was as follows: all subjects received an injection of PP1M (dose range 50–150 mg eq.) every 4 weeks for 4 months; they were then switched to PP3M (dose range 175–525 mg eq.) with an injection every 12 weeks for one year. Because the subjects were exposed to paliperidone before the trial, we estimated visually the quantity in plasma at the start of the trial. For each subject, this quantity (in mg) was sampled from a lognormal distribution with geometric mean 30 mg and variance 1.5 in log scale.

The model predicts "true" or rather average plasma concentrations of paliperidone in virtual subjects. It can also simulate measured concentrations (simulated data) by adding a random error to the predicted average plasma concentration (Figure 1). The distribution of this random error is given by the published residual error model [2]; it accounts for measurement error, inter-occasion variability and modeling error. Figure 2 shows simulation results for a small cohort of 20 subjects. The code used for those plots is below (Population PK model implementation in Nimble R (v8_pop)).

Plots of the predicted *vs*. observed (by Magnusson *et al.* [2]) median, fifth percentile and 95th percentile PP3Mr plasma concentrations at the various measurement times are shown in Figure 3. Practically all predictions are within a factor 2 away from the observation. Ratios averaged over time are given in Table 1. The average ratios for the median concentrations do not exceed 1.25.

Table 1: Average predicted-over-observed median, fifth percentile and 95th percentile PP3Mr plasma concentration ratios for the different trial doses presented in Magnusson *et al.* [2].

Dose	Median ratio	5 %tile ratio	95 %tile roadio
525	1.05	0.97	1.26
350	1.08	0.99	1.22
263	1.12	1.00	1.16
175	1.24	1.18	1.54

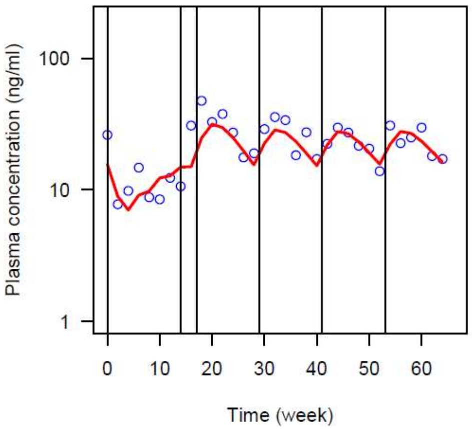

Figure 1: Simulation of plasma PP concentrations for a virtual subject with the joint PP1M (INVEGA) and PP3M (TRINZA) models[2]. Four injections of PP1M (75 mg eq.) are followed by four injections of PP3M (263 mg eq.). Parameter values were set to the best estimates given in the original publication[2]. Red line: average (predicted) plasma concentration of paliperidone; Blue points: simulated concentration measurements, with random error.

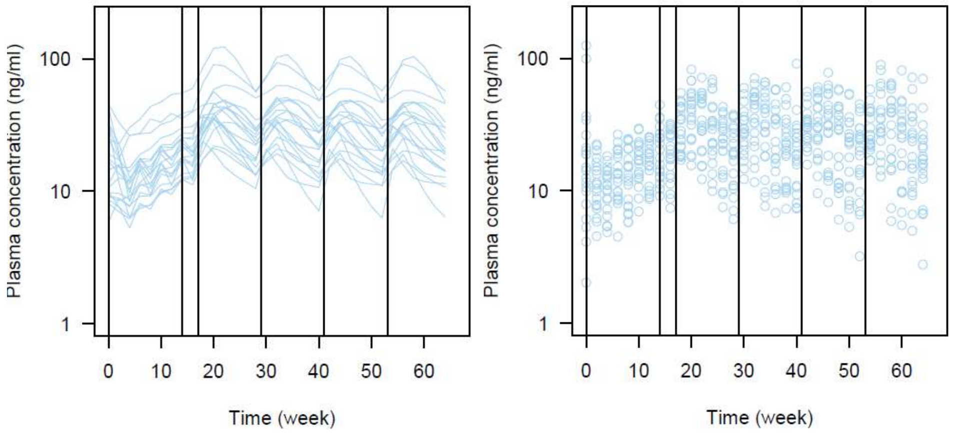

Figure 2: Simulation plasma PP concentrations for 20 virtual subjects with the PP1M (INVEGA) and PP3M (TRINZA) models[2]. Four injections of PP1M (75 mg eq.) are followed by four injections of PP3M (263 mg eq.). Parameter values were set to the best estimates given in the original publication[2]. Left panel: average (predicted) plasma concentrations of paliperidone; Right panel: simulated concentration measurements, with random error.

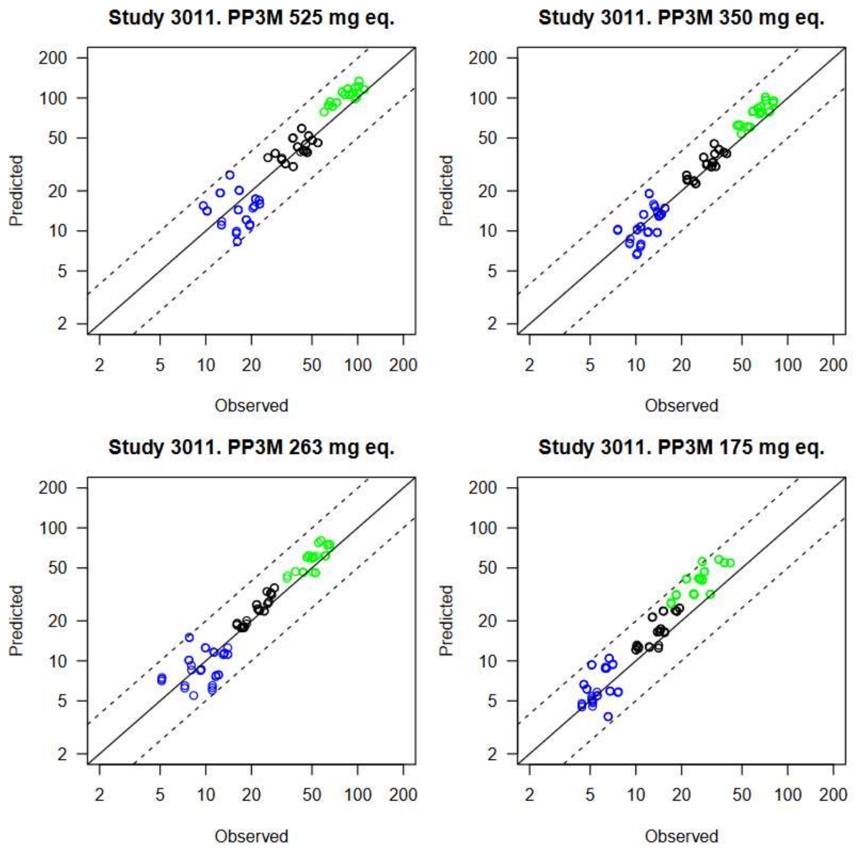

Figure 3: Predicted *vs*. observed (by Magnusson *et al.* [2]) medians (black), fifth percentiles (blue) and 95th percentiles (green) PP plasma concentrations at the various measurement times over the four PP3Mr (TRINZA) dosing periods. Parameter values were set to the best estimates given in the original publication.

4 Sensitivity analysis of impact of drug-release parameters on C_{max} and AUC

We investigated the effect of changing the drug-release and absorption parameters of the PP3Mt model on the maximum PP plasma concentration (C_{max}) and the area under the curve (AUC) in the last PP3Mt dosing period. Those drug-release parameters of the model are $k_{ar3,max}$, $k_{ar3,50}$, $k_{as3,max}$, $k_{as3,50}$, γ, and f_3. The population mean of each drug-release parameter was changed one-at-a-time by ±5%, *i.e.*, by setting the relative change δ_i to 0.95 and 1.05. The same dosing regimen and sampling scheme as in the Magnusson et al.'s trial were applied. Only the highest doses for PP1M (150 mg eq.) and PP3Mt (525 mg eq.) were tested.

An analysis was run for one subject to quantify the impact on C_{max} and AUC of modifying by 5% the drug-release parameters. Figure 4 (left two panels) shows that in that case f_3, $k_{as3,max}$ and $k_{ar3,max}$ can impact C_{max} up to 2.2%, and that the AUC can be modified up to 3% by f_3 and $k_{as3,max}$. These influential parameters are positively correlated with the two PK parameters. C_{max} and AUC are most sensitive to $k_{as3,max}$ which controls PP release from the slow-release depot. For LAI products, absorption conditions the concentration decay phase (flip-flop) and we are using partial AUC at steady-state. So, the release rates logically condition trough concentrations, and therefore C_{max} and AUC. Figure 4, right panel, also shows how these small changes in the parameters' values affect PP plasma kinetic profile during the last dosing period (between 53 and 65 weeks, with blood sampling at weeks 54, 55, 57, 61 and 65).

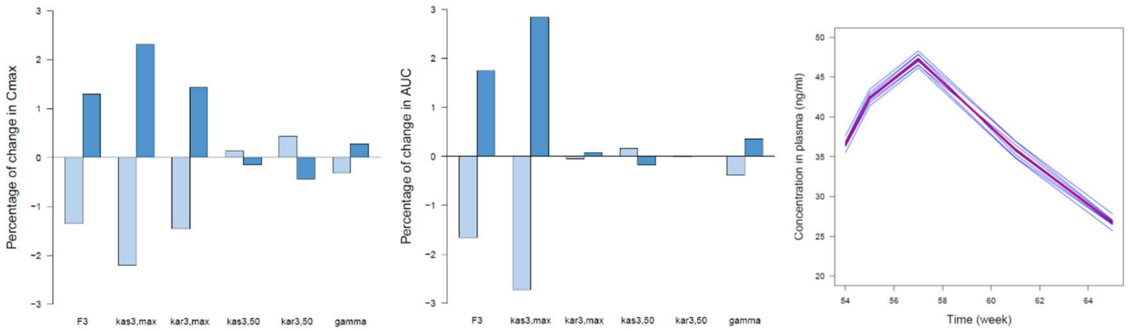

Figure 4: Percentages of change in C_{max} (left panel) and AUC (middle panel) when the model drug-release parameters are decreased (light blue) or increased (dark blue) by 5%. Right panel: predicted PP plasma concentration when the values of the drug-release parameters are set to the reference values (red curve) or modified by 5% (blue curves). Four injections of PP1M (150 mg eq.) were followed by four injections of either PP3Mr or PP3Mt (525 mg eq.). The plot is for the last dosing period.

5 Power Calculations (Workflow A)

5.1 Methods

Power is the probability P of declaring BE when the test formulation is actually bioequivalent to the reference formulation. It is equal to 1 minus type II error, which measures producer risk (the probability of declaring non-BE when the products are in fact bioequivalent. In a simulation context, if all model parameters distributions for the test formulation model are exactly the same as for the reference formulation model (in the case of the population PK model we use, all components of the

vector δ at value 1) then we are certain that test and reference are bioequivalent, and we can compute power.

If the test and reference formulations are not strictly identical (some components of $\delta \neq 1$), we can still estimate the probability P of declaring BE, but we should not call it "power", even though the formulations might still be bioequivalent in terms of C_{max} and AUC (if the structural parameters' relative differences δ are small enough).

In any case, the probability of declaring BE for a given value of δ can be calculated with the following pseudo-algorithm:

1. Simulate 1000 virtual trials of arm size N_{max} with δ set at the chosen value (we took N_{max} = 500 subjects per arm). Only N virtual subjects among N_{max} will be used to compute power at arm size N (lower or equal to N_{max}); For that:
2. Set arm size N to 2.
3. For each one of the 1000 virtual trials, draw randomly (without replacement) N subjects, assess and record BE for C_{max} and AUC using a TOST test; global BE is declared if BE is declared for both C_{max} and AUC.
4. Compute probability P at arm size N as the number of trials that declared BE over the total number of simulated trials (1000).
5. Increment arm size by 1.
6. Go to step 3 if arm size ≤ N_{max}, otherwise go to step 7.
7. End; report probability P for each arm size.

See workflow code v4 below.

5.2 Results

The results of the above calculations are shown on Figure 5 for several values of δ_2 (the test over reference ratio of the drug-release parameter $k_{as3,max}$). All the other components of δ were set to 1. In the case of perfect bioequivalence (δ_2 = 1), 80% power or better is expected with 120 virtual subjects per arm or more. Power decreases when difference δ_2 increases; for example, with δ_2 = 1.2, more than 200 virtual subjects would probably be needed to reach 80% power.

Table 2 gives a numerical summary of Figure 5. With 130 subjects per arm, power (the probability of declaring bioequivalence when δ_2 = 1.0) is just slightly above 80%.

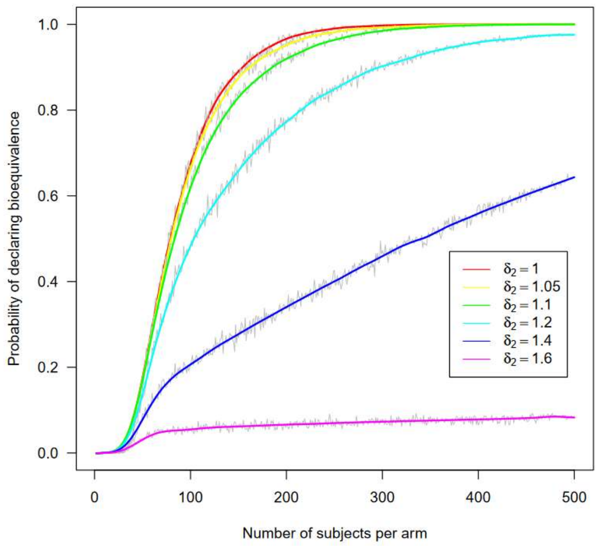

Figure 5: Estimated probability P of declaring bioequivalence as a function of the number of subjects per arm in a PP virtual clinical BE trial. The PP population PK model was used to simulate 1000 virtual trials with different values of δ_2 (the test over reference ratio of the drug-release parameter $k_{as3,max}$). BE was assessed for C_{max} and AUC with a TOST test.

Table 2: Probability of declaring bioequivalence as a function of the number of subjects per arm in a PP virtual clinical BE trial and the underlying true value of δ_2. BE was assessed for C_{max} and AUC with a TOST test.

δ_2	Number of subjects per arm					
	100	130	200	300	400	500
1.00	0.68	**0.83**	0.97	1.00	1.00	1.00
1.05	0.66	0.81	0.95	0.99	1.00	1.00
1.10	0.62	0.77	0.92	0.99	1.00	1.00
1.20	0.48	0.60	0.77	0.90	0.96	0.98
1.40	0.20	0.25	0.34	0.46	0.56	0.64
1.60	0.06	0.06	0.06	0.07	0.08	0.08

6 Type I Error Analysis (Workflow A)

6.1 Methods

Type I error is the probability of declaring BE when the test formulation is actually *not* bioequivalent to the reference formulation. It measures a consumer risk. In a simulation context, it is necessary to generate trials with differences between test and reference. This can be done by sampling vector δ

values in a large range. The trials for which the simulated data-based ratio of test over reference for C_{max} or AUC is outside the range [0.8, 1.25] can be considered as truly non-bioequivalent, if the trial size is large. We can then use the TOST to get its opinion on BE and check the fraction of trials for which TOST declares BE when the trial was in fact non-BE. This fraction is an estimate of type 1 error.

Type I error was therefore calculated with the following pseudo algorithm:

1. Simulate 1000 virtual trials of arm size N_{max} (we took N_{max} = 500 subjects per arm).
2. For each one of the 1000 virtual trials, sample each element of δ from a uniform [0.5,2] distribution. Compute geometric mean ratios test over reference for C_{max} and AUC for the simulated data. Assess and record BE for C_{max} and AUC using a TOST test; global BE is declared if BE is declared for both C_{max} and AUC.
3. Probability of declaring BE is the number of trials that declared BE over the total number of simulated trials (1000). Type I error is the probability of declaring BE when the C_{max} and AUC ratios are outside interval [0.8, 1.25].

6.2 Results

Figure 6 shows the probability of declaring BE with TOST as a function of the actual differences in C_{max} and AUC ratios for 1000 virtual trials. We can see that in no case BE was declared when the raw data showed non-compliant differences in C_{max} or AUC. Actually, outside the [0.9, 1.1] interval, approximately, BE was never declared. This is because TOST judges BE on the basis of confidence limits, and because there is also a producer risk (power is not always 100% with a size 1000 per arm trial). More precise type I error calculations would require computing the fraction of false positive TOST BE in different bins of difference values, but in any case, near the borders 0.8 and 1.25 those fractions would clearly be null. Overall, we can conclude that consumer risk is very small.

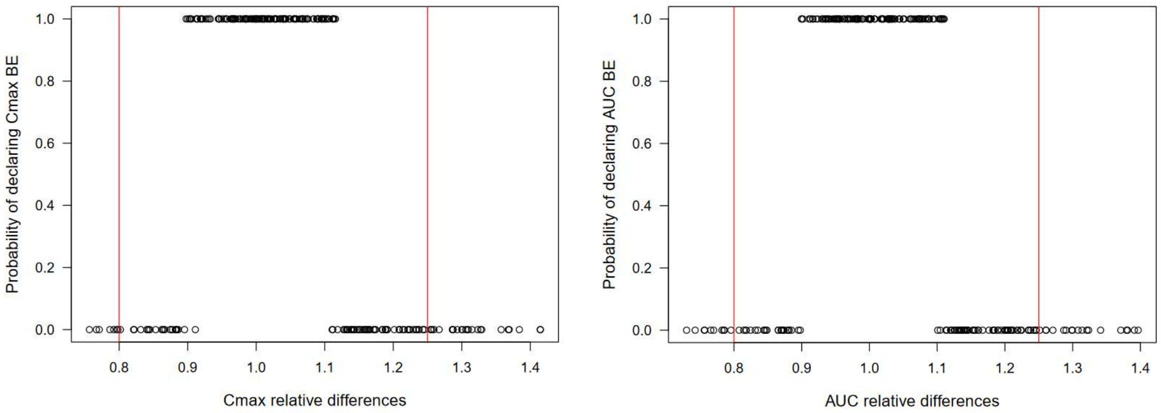

Figure 6: Estimated probability P of declaring bioequivalence as a function geometric of the data-based mean ratio test over reference for C_{max} (left panel) or AUC (right panel) in 1000 PP virtual clinical BE trials with 500 subjects per arm. The PP population PK model was used to simulate each trial with different values of vector δ (the test over reference ratios for the population PK model absorption parameters). BE was assessed for C_{max} and AUC with a TOST test.

7 Abbreviated clinical trial simulation summary plot

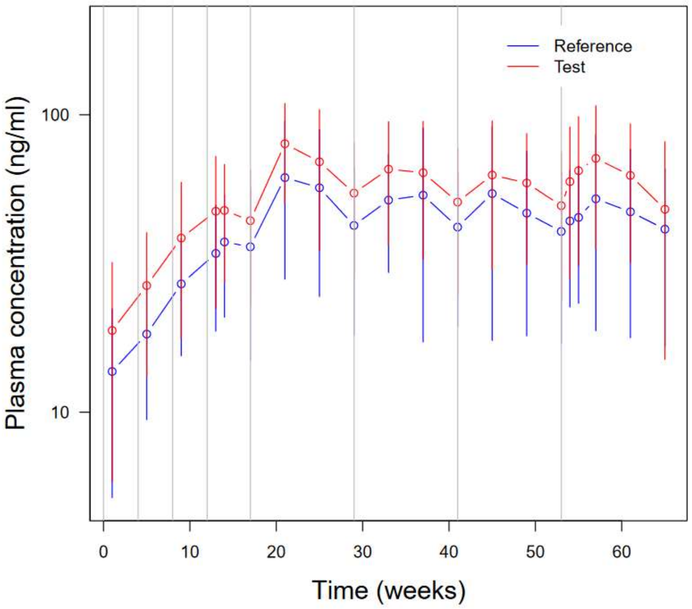

Figure 7: Simulated plasma PP concentrations averages of subjects (25 per arm) for the two arms of the simulated parallel abbreviated virtual trial when parameter $k_{as3,max}$ was increased by 5% from the value of the reference formulation. The subjects received four injections of PP1M (150 mg eq.) prior to four injections (525 mg eq.) of PP3Mr (blue) or PP3Mt (red). The vertical bars span ± 1 SD around the averages. The grey lines mark injection times.

8 Convergence of the model recalibration by MCMC sampling

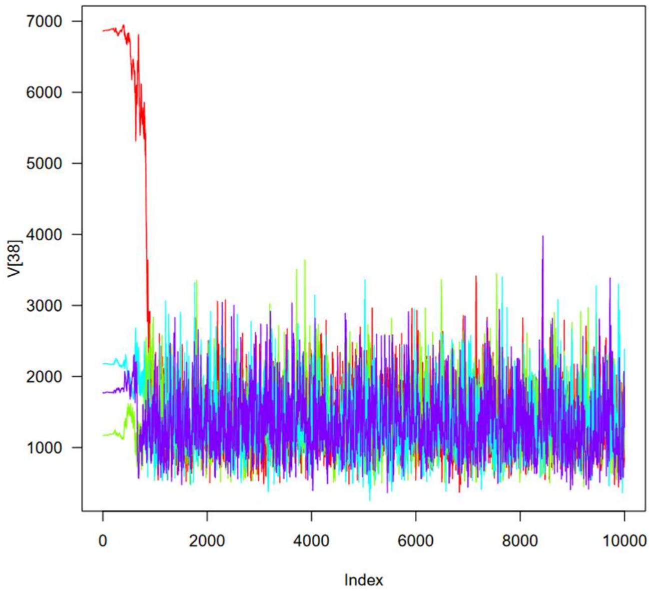

Figure 8: Illustration of MCMC sampling convergence. The values of parameter V (for virtual subject 38) sampled by four simulated Markov chains (different colors) are plotted against the the number of iterations. The chains started from different random values and converged in probability to the target distribution only after about 1000 iterations. For all parameters, the first 2500 iterations were discarded to make sure that only values at convergence were kept.

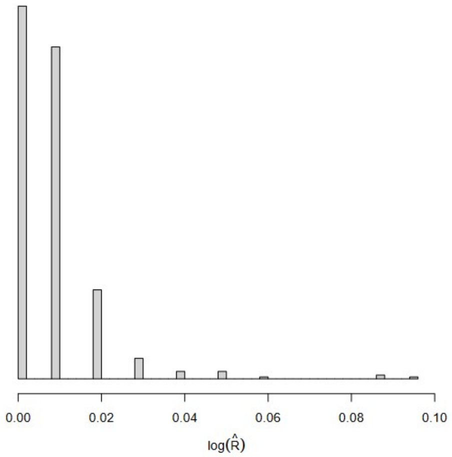

Figure 9: Histogram (over 451 model parameters) of the logarithms of the MCMC convergence diagnostic $\hat{R}$.

9 Posterior distribution summary for parameter δ_2

Table 3: Summary statistics and convergence diagnostic $\hat{R}$ for the marginal posteriors of log (δ_2), actually sampled, and δ_2.

Parameter	Geometric Mean	Geometric SD	2.5 %tile	Median	97.5 %tile	$\hat{R}$
log (δ_2)	0.349	0.175	0.00	0.352	0.680	1.01
δ_2	1.42	1.19	1.00	1.42	1.97	1.01

10 Observations *vs*. predictions plot for the recalibration step

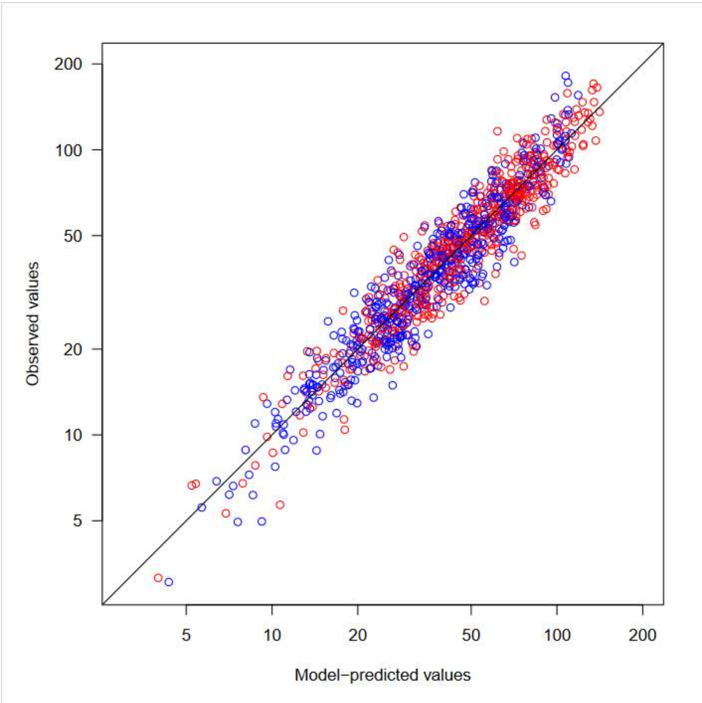

Figure 10: Observed PP plama concentations *vs*. corresponding posterior predictions with the maximum posterior (most likely) population PK parameter values. In blue: reference formulation group; in red: test group.

11 Large virtual trial trial simulation summary plot

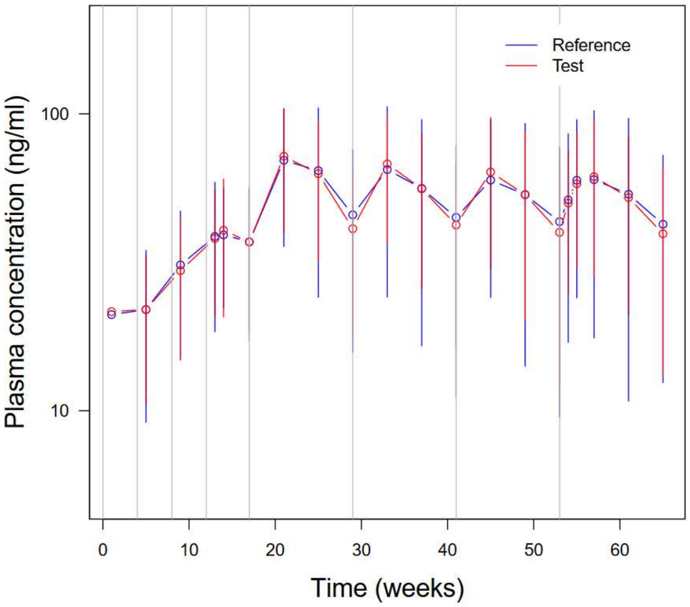

Figure 11: Simulated plasma PP concentrations averages of subjects (130 per arm) for the two arms of the simulated parallel virtual trial. The difference between $k_{as3,max}$ population means in test and reference formulations, δ_2, was set to 1.42 (geometric mean of its posterior distribution). By chance, this difference does *not* translate into large differences between test and reference PP plasma concentrations. The subjects received four injections of PP1M (150 mg eq.) prior to four injections (525 mg eq.) of PP3Mr (blue) or PP3Mt (red). The vertical bars span ± 1 SD around the averages. The grey lines mark injection times.

12 Full safe-space calculations for the data-based workflow

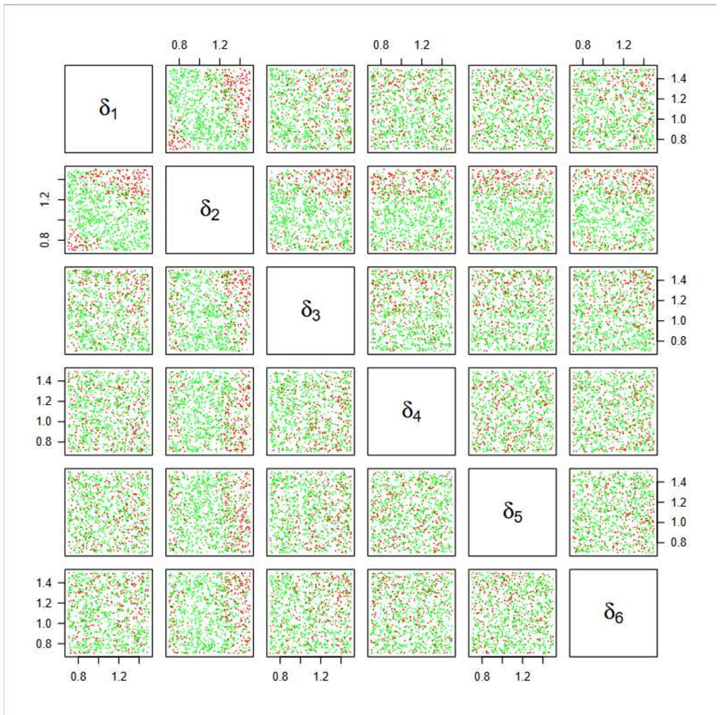

Figure 12: Data-based BE safe-space regions for the six drug-release parameters of the PP population PK model. The green dots marks the PP trials (1000 trials, 500 subjects per arm) for which BE was declared using the TOST test; the red dots indicate failing trials.

13 Computer codes

13.1 Structural model C code (v4)

```
/* compile within R with system("R CMD SHLIB PP3M_model.c")
   V04
*/
#include <R.h>

#define Nparms 12

static double parms[Nparms];

/* A trick to keep up with the parameters */
#define Dose_PP1M    parms[0]
#define F2           parms[1]
#define Duration_2   parms[2]
#define ka_PP1M      parms[3]
#define ka1_max      parms[4]
#define ka3_max      parms[5]
#define kamt1_50     parms[6]
#define kamt3_50     parms[7]
#define gamma        parms[8]
#define CL           parms[9]
#define V            parms[10]
#define PP3M_start   parms[11]

/* initializer: same name as the dll (without extension) */
```

```
void PP13M_model_v04(void (* odeparms)(int *, double *))
{
    int N = Nparms;
    odeparms(&N, parms);
}

/* Derivatives */
void derivs(int *neq, double *t, double *y, double *ydot, double *yout, int*ip)
{
  double Ke;

  // State variables
  // Q_depot_s1 = y[0] # quantity (mg) in PP1M slow absorption depot
  // Q_depot_s3 = y[1] # quantity (mg) in PP3M slow absorption depot
  // Q_depot_r3 = y[2] # quantity (mg) in PP3M fast absorption depot
  // Q_central  = y[3] # quantity (mg) in central compartment

  // ODEs
  // Quantity in PP1M depot slow absorption
  ydot[0] = -ka_PP1M * y[0];

  // Quantities in PP3M depots
  if (*t < PP3M_start) { // use PP1M model, PP3M model differentials are null
    // Quantity in PP3M depot slow absorption
    ydot[1] = 0;
    // Quantity in PP3M depot rapid absorption
    ydot[2] = 0;
  }
  else { // use PP1M and PP3M models concurrently
    // Quantity in PP3M depot slow absorption
    ydot[1] = -ka1_max * pow(y[1], gamma) /
               (pow(kamt1_50, gamma) + pow(y[1], gamma));
    // Quantity in PP3M depot rapid absorption
    ydot[2] = -ka3_max * y[2] / (kamt3_50 + y[2]);
  }

  // Quantity in central compartment
  // clearance from central
  Ke = CL / V;
  // hard-code the zero-order inputs after PP1M injections
  if (((0 <= *t)    && (*t < 319))  || ((672 <= *t)  && (*t < 991)) ||
      ((1344 <= *t) && (*t < 1663)) || ((2016 <= *t) && (*t < 2335))) {
    ydot[3] = F2 * Dose_PP1M / Duration_2
              -(ydot[0] + ydot[1] + ydot[2]) - Ke * y[3];
  }
  else {
    ydot[3] = -(ydot[0] + ydot[1] + ydot[2]) - Ke * y[3];
  }
}
/* End */
```

13.2 Population PK model implementation in Nimble R (v8_pop)

```
## v8_pop: Parameters of the population distributions are distributed.
##         Modify the PK model (v04).

library(nimble)
## compile C ODE model for deSolve
Cmodel.name = "PP13M_model_v04"
dyn.load(paste(Cmodel.name, .Platform$dynlib.ext, sep = ""))

## ---------------------------------------------------------------------
## ODE solver: performs a simulation, given initial state values,
```

```
## output times and time-constant, scaled, parameters
R_ode = function(y, times, parms) {
  ## params = c( 1:Dose_PP1M, 2:F2,      3:Duration_2,  4:ka_PP1M,
  ##             5:ka1_max,   6:ka3_max, 7:kamt1_50,    8:kamt3_50, 9:gamma,
  ##            10:CL,       11:V,      12:PP3M_start, 13:F3,      14:Dose_PP3M,
  ##            15:Q_cen_0)
  ## The last two parameters should not be passed to the ODE solver, they are
  ## used here only.
  ## State variables (y) initial conditions
  y = c("Q_depot_s1" = 0,
        "Q_depot_s3" = 0,
        "Q_depot_r3" = 0,
        "Q_central"  = parms[15])
  ## The doses are specified as "events" affecting the state variables
  ## dosing times (hours)
  dose_times = c(c(0, 4, 8, 12) + parms[3] / (24 * 7),
                 17, 29, 41, 53) * 24 * 7
  N_doses = length(dose_times)
  ## changing state variables at dosing times
  vars = c("Q_depot_s1", "Q_depot_s3", "Q_depot_r3")
  v1 = (1 -  parms[2])  * parms[1]     # value applied to Q_depot_s1
  v2 = (1 -  parms[13]) * parms[14]    # value applied to Q_depot_s3
  v3 =       parms[13]  * parms[14]    # value applied to Q_depot_r3
  ## Form the events table
  eventdat = data.frame(var = c(rep("Q_depot_s1", 4), rep(vars[2:3], 4)),
                        time = c(dose_times[1:4], rep(dose_times[5:8], each=2)),
                        value = c(rep(v1, 4),
                                  rep(c(v2, v3), N_doses - 4)),
                        method = "add")
  ## Integrate numerically, with outputs at specified times
  result = deSolve::ode(y, times, func="derivs", parms=parms[1:12],
                        rtol=1e-6, atol=1e-6, dllname="PP13M_model_v04",
                        initfunc = "PP13M_model_v04",
                        events=list(data=eventdat))
  result = result[which(result[,1] %in% times),] # weed out extra times
  if (dim(result)[[1]] < length(times)) { ## integration failed
    return(rep(1E-30, length(times)))
  } else {
    ## compute central concentration, convert from mg/L to ng/ml, return
    return(result[,5] * 1E3 / parms[11])
  }
} # end of R_ode model solver

## ---------------------------------------------------------------------------
## Nimble function with nimbleRcall. This is just a wrapper
nimble_ode = nimbleRcall(
  prototype = function(
    y     = double(1), # vector
    times = double(1), # vector
    parms = double(1)  # vector
  ) {},
  returnType = double(1), # outcome is a vector
  Rfun = 'R_ode')

## ---------------------------------------------------------------------------
## Hierarchical core Nimble (BUGS) code
myNimbleCode = nimbleCode({ ## BUGS (extended) code
  ## population mean (with prior if not fixed)
  F2t_m       <- logit(0.168) # From Samtani paper Table III.
  # from online resource 3 Magnusson, F2 = 0.153
  F3t_m       <- logit(0.209) # transformed F3
  ka1_pp1m_mv <- log(1 + 0.02^2)
  ka1_pp1m_m  ~  dlnorm(meanlog=log(4.88E-4), varlog=ka1_pp1m_mv) # (1/h)
  ka1_max_m   ~  dnorm(mean = 0.0904,  sd = 0.0696 * 0.0904)  # (mg/h)
  ##ka1_max_m   <-  0.0904  # (mg/h)
```

```
ka3_max_m   ~  dnorm(mean = 0.164,   sd = 0.0465 * 0.164)   # (mg/h)
kamt1_50_m  ~  dnorm(mean = 120,     sd = 0.0383 * 120)     # (mg)
kamt3_50_m  ~  dnorm(mean = 21.4,    sd = 0.0952 * 21.4)    # (mg)
gamma_m     ~  dnorm(mean = 1.44,    sd = 0.0165 * 1.44)    # unitless
CL_m        ~  dnorm(mean = 3.84,    sd = 0.0216 * 3.84)    # (1/hr)
V_m         <- 1960                                         # (L)
##
## pop (inter-individual) SDs (with prior if not fixed)
if (SamEq3) { # SD or CV applies to F2, so: detransform
  ## F2_sd
  omega2    <- 0.064 / (0.168 * (1 - 0.168)) # Samtani eq. 3
  F2_sd     ~  dnorm(mean = omega2, sd = 0.02*omega2)
  F2_v      <- F2_sd^2
  ## F3_v
  F3_v      <- (0.854 * 0.209 / (0.209 * (1 - 0.209)))^2 # Samtani eq. 3
} else { # SD or CV applies to F2 transformed
  F2_sd     ~  dnorm(mean = 0.064, sd = 0.02*0.064)
  F3_v      <- (abs(F3t_m) * 0.854)^2
}
##
ka1_pp1m_cv ~  dnorm(mean = 0.590, sd = 0.03*0.59)
ka1_pp1m_v  <- log(1 + ka1_pp1m_cv^2)
##
ka1_max_cv  ~  dnorm(mean = 0.827, sd = 0.0501 * 0.827)
ka1_max_v   <- log(1 + ka1_max_cv^2)
##
ka3_max_v   <- 0
##
kamt1_50_cv ~  dnorm(mean = 0.500, sd = 0.101  * 0.500)
kamt1_50_v  <- log(1 + kamt1_50_cv^2)
##
kamt3_50_cv ~  dnorm(mean = 0.867, sd = 0.142  * 0.867)
kamt3_50_v  <- log(1 + kamt3_50_cv^2)
##
gamma_v     <- 0
##
CL_cv_v     <- log(1 + 0.0317^2)
CL_cv       ~  dlnorm(meanlog=log(0.357), varlog=CL_cv_v)
CL_v        <- log(1 + CL_cv^2)
##
V_v         <- log(1 + 0.628^2)
##
## measurement error variance in log for plasma concentration, ng/ml
## if we want uncertainty on the residual error we should use:
res_cv_v    <- log(1 + 0.321^2)
res_cv      ~  dlnorm(meanlog=log(0.306), varlog=res_cv_v)
sigma2      <- log(1 + res_cv^2)
##
## for each subject
for (i in 1:nsubjects) {
  tmp2[i]     ~  dnorm(mean = F2t_m, var = F2_v)
  F2[i]       <- ilogit(tmp2[i])
  tmp3[i]     ~  dnorm(mean = F3t_m, var = F3_v)
  F3[i]       <- ilogit(tmp3[i])
  ka_PP1M[i]  ~  dlnorm(meanlog=log(ka1_pp1m_m), varlog=ka1_pp1m_v) # (1/hr)
  ka1_max[i]  ~  dlnorm(meanlog=log(ka1_max_m),  varlog=ka1_max_v)  # (mg/h)
  ka3_max[i]  ~  dlnorm(meanlog=log(ka3_max_m),  varlog=ka3_max_v)  # (mg/h)
  kamt1_50[i] ~  dlnorm(meanlog=log(kamt1_50_m), varlog=kamt1_50_v) # (mg)
  kamt3_50[i] ~  dlnorm(meanlog=log(kamt3_50_m), varlog=kamt3_50_v) # (mg)
  gamma[i]    ~  dlnorm(meanlog=log(gamma_m),    varlog=gamma_v)    # no unit
  CL[i]       ~  dlnorm(meanlog=log(CL_m),       varlog=CL_v)       # (1/hr)
  V[i]        ~  dlnorm(meanlog=log(V_m),        varlog=V_v)        # (L)
  ##
  Q_cen_0[i]  ~  dlnorm(meanlog=log(Q_cen_0_mean), sdlog = log(Q_cen_0_sd))
```

```
    ##
    ## likelihood for concentration measurements
    ## Call the ODE solver to get predictions for each subject
    ## predictions are plasma concentrations, in ng/ml
    Conc[i, 1:ntimes] <- nimble_ode(y[1:nstates], times[1:ntimes],
                                    c(Dose_PP1M, F2[i], Duration_2,
                                      ka_PP1M[i], ka1_max[i], ka3_max[i],
                                      kamt1_50[i], kamt3_50[i], gamma[i],
                                      CL[i], V[i], PP3M_start, F3[i],
                                      Dose_PP3M, Q_cen_0[i]))
    ## data likelihood
    for (j in 1:ntimes) {
      C_plasma_obs[i,j] ~ dlnorm(meanlog=log(Conc[i,j]), varlog=sigma2)
    }
  }
}) # End myNimbleCode

## ---------------------------------------------------------------------------
## Build and compile the model for predictions with various PP1M doses
## show the difference between data and mean
N = 1 # number of trials
nsubjects = 1 # number of subjects

times = seq(0, 65, 2) * 7 * 24 # up to 65 weeks, in hours

dose_pp1m = c(150, 100, 75,  50)
dose_pp3m = c(525, 350, 263, 175)
Q_cen_0_mean = 30 # geo mean
Q_cen_0_sd   = 1.5  # geo SD
data  = list()
inits = list(Q_cen_0_mean = Q_cen_0_mean, # (mg)
             Q_cen_0_sd   = Q_cen_0_sd,   # (mg)
             Dose_PP1M    = dose_pp1m[1], # (mg)
             Dose_PP3M    = dose_pp3m[1]) # (mg)

constants = list(nsubjects  = nsubjects,
                 SamEq3     = 1,             # Boolean
                 Duration_2 = 319,           # (h)
                 PP3M_start = 17*7*24,       # (h)
                 ntimes     = length(times),
                 times      = times,
                 nstates    = 4)

Rmodel = nimbleModel(myNimbleCode, constants, data, inits)
Cmodel = compileNimble(Rmodel, showCompilerOutput=F)

Node.names = Cmodel$getNodeNames(includeData=T)
Cmodel$simulate(nodes = Node.names)
res = values(Cmodel, Node.names)

par(mfrow=c(2,2), mar=c(4,4,3,1))
plot.times = times/(24*7)
for (j in 1:length(dose_pp3m)) { # for each dose of PP3M
  Cmodel$Q_cen_0_mean = Q_cen_0_mean
  Cmodel$Q_cen_0_sd   = Q_cen_0_sd
  Cmodel$Dose_PP1M = dose_pp1m[j]
  Cmodel$Dose_PP3M = dose_pp3m[j]

  Cmodel$simulate(nodes=Node.names)
  sub.mean_vector = values(Cmodel, "Conc")
  sub.data_vector = values(Cmodel, "C_plasma_obs")
  sub.mean = matrix(sub.mean_vector, nrow=nsubjects, byrow = F)
  sub.data = matrix(sub.data_vector, nrow=nsubjects, byrow = F)

  ## plot individual means
```

```
  plot (plot.times, times, type="n", col="red", lwd=2, log="y",
        las=1, xlab="Time (week)", ylab="Plasma concentration (ng/ml)",
        main=paste("Study 3011. PP3M", dose_pp3m[j] ,"mg eq."),
        xlim=c(0, 66), ylim=c(1, 200),
        yaxp=c(1, 100, 1)) # ylim should be adapted!!!!

  for (i in 1:nsubjects) {
    lines(plot.times, sub.data[i,], type ="p", col="blue",lwd=1)
    lines(plot.times, sub.mean[i,], type ="l", col="red", lwd=2)
  }
  abline(v=c(0, 14, 17, 29, 41, 53))
}

## ----------------------------------------------------------------------------
## Build and compile the model for predictions with various PP1M doses
## small clinical study
N = 1 # number of trials
nsubjects = 20 # number of subjects

times = seq(0, 65, 2) * 7 * 24 # up to 65 weeks, in hours

dose_pp1m = c(150, 100, 75,  50)
dose_pp3m = c(525, 350, 263, 175)
Q_cen_0_mean = 30 # geo mean
Q_cen_0_sd   = 1.5  # geo SD
data  = list()
inits = list(Q_cen_0_mean = Q_cen_0_mean, # (mg)
             Q_cen_0_sd   = Q_cen_0_sd,   # (mg)
             Dose_PP1M    = dose_pp1m[1], # (mg)
             Dose_PP3M    = dose_pp3m[1]) # (mg)

constants = list(nsubjects  = nsubjects,
                 SamEq3     = 1,            # Boolean
                 Duration_2 = 319,          # (h)
                 PP3M_start = 17*7*24,      # (h)
                 ntimes     = length(times),
                 times      = times,
                 nstates    = 4)

Rmodel = nimbleModel(myNimbleCode, constants, data, inits)
Cmodel = compileNimble(Rmodel, showCompilerOutput=F)

Node.names = Cmodel$getNodeNames(includeData=T)
Cmodel$simulate(nodes = Node.names)
res = values(Cmodel, Node.names)

par(mfrow=c(2,2), mar=c(4,4,3,1))
plot.times = times/(24*7)
for (j in 1:length(dose_pp3m)) { # for each dose of PP3M
  Cmodel$Q_cen_0_mean = Q_cen_0_mean
  Cmodel$Q_cen_0_sd   = Q_cen_0_sd
  Cmodel$Dose_PP1M = dose_pp1m[j]
  Cmodel$Dose_PP3M = dose_pp3m[j]

  Cmodel$simulate(nodes=Node.names)
  sub.mean_vector = values(Cmodel, "Conc")
  sub.data_vector = values(Cmodel, "C_plasma_obs")
  sub.mean = matrix(sub.mean_vector, nrow=nsubjects, byrow = F)
  sub.data = matrix(sub.data_vector, nrow=nsubjects, byrow = F)

  plot (plot.times, times, type="n", col="red", lwd=2, log="y",
        las=1, xlab="Time (week)", ylab="Plasma concentration (ng/ml)",
        main=paste("Study 3011. PP3M", dose_pp3m[j] ,"mg eq."),
        xlim=c(0, 66), ylim=c(1, 200),
```

```
        yaxp=c(1, 100, 1)) # ylim should be adapted!!!!

  for (i in 1:nsubjects) {
    # plot individual mean
    #lines(plot.times, sub.mean[i,], type ="l", col="lightskyblue2",lwd=0.5)
    ## plot individual data
    lines(plot.times, sub.data[i,], type ="p", col="lightskyblue2",lwd=0.5)
  }
  abline(v=c(0, 14, 17, 29, 41, 53))
}

## ---------------------------------------------------------------------------
## Build and compile the model for predictions with various PP1M doses
## N =100 and nsbjects =130

nsubjects = 130 # number of subjects

## times = seq(0, 65, 1) * 7 * 24 # up to 65 weeks, in hours
times = c(0, seq(0, 65*7*24, 7*6)+12) ## (hours)
dose_pp1m = c(150, 100, 75,  50)
dose_pp3m = c(525, 350, 263, 175)
Q_cen_0_mean = 30 # geo mean
Q_cen_0_sd   = 1.5  # geo SD
data  = list()
inits = list(Q_cen_0_mean = Q_cen_0_mean, # (mg)
             Q_cen_0_sd   = Q_cen_0_sd,   # (mg)
             Dose_PP1M    = dose_pp1m[1], # (mg)
             Dose_PP3M    = dose_pp3m[1]) # (mg)

constants = list(nsubjects  = nsubjects,
                 SamEq3     = 1,            # Boolean
                 Duration_2 = 319,          # (h)
                 PP3M_start = 17*7*24,      # (h)
                 ntimes     = length(times),
                 times      = times,
                 nstates    = 4)

Rmodel = nimbleModel(myNimbleCode, constants, data, inits)
Cmodel = compileNimble(Rmodel, showCompilerOutput=F)

Node.names = Cmodel$getNodeNames(includeData=T)
Cmodel$simulate(nodes = Node.names)
res = values(Cmodel, Node.names)

Magnus1 = read.csv("Magnusson_Figure6_Panel1.csv")
Magnus2 = read.csv("Magnusson_Figure6_Panel2.csv")
Magnus3 = read.csv("Magnusson_Figure6_Panel3.csv")
Magnus4 = read.csv("Magnusson_Figure6_Panel4.csv")

N = 100 # number of trials

par(mfrow=c(2,2), mar=c(4,4,3,1))
plot.times = times/(24*7)
for (j in 1:length(dose_pp3m)) { # for each dose of PP3M
  Cmodel$Q_cen_0_mean = Q_cen_0_mean
  Cmodel$Q_cen_0_sd   = Q_cen_0_sd
  Cmodel$Dose_PP1M = dose_pp1m[j]
  Cmodel$Dose_PP3M = dose_pp3m[j]
  ## Monte Carlo simulations for mean and data predictions for random subjects
  quant.mean.p5.all  = matrix(0, nrow=N,
                              ncol=length(values(Cmodel, "Conc"))/nsubjects)
  quant.mean.p50.all = matrix(0, nrow=N,
                              ncol=length(values(Cmodel, "Conc"))/nsubjects)
  quant.mean.p95.all = matrix(0, nrow=N,
                              ncol=length(values(Cmodel, "Conc"))/nsubjects)
```

```
quant.data.p5.all  = matrix(0, nrow=N,
                            ncol=length(values(Cmodel,
                                              "C_plasma_obs"))/nsubjects)
quant.data.p50.all = matrix(0, nrow=N,
                            ncol=length(values(Cmodel,
                                              "C_plasma_obs"))/nsubjects)
quant.data.p95.all = matrix(0, nrow=N,
                            ncol=length(values(Cmodel,
                                              "C_plasma_obs"))/nsubjects)

for (i in 1:N) { # for each trial

  Cmodel$simulate(nodes=Node.names)
  sub.mean_vector = values(Cmodel, "Conc")
  sub.data_vector = values(Cmodel, "C_plasma_obs")

  sub.mean = matrix(sub.mean_vector, nrow=nsubjects, byrow = F)
  sub.data = matrix(sub.data_vector, nrow=nsubjects, byrow = F)

  quant.mean = apply(sub.mean, MAR=2, FUN=quantile, probs=c(0.05, 0.5, 0.95))
  quant.data = apply(sub.data, MAR=2, FUN=quantile, probs=c(0.05, 0.5, 0.95))

  quant.mean.p5.all[i,]  = quant.mean[1,]
  quant.mean.p50.all[i,] = quant.mean[2,]
  quant.mean.p95.all[i,] = quant.mean[3,]

  quant.data.p5.all[i,]  = quant.data[1,]
  quant.data.p50.all[i,] = quant.data[2,]
  quant.data.p95.all[i,] = quant.data[3,]
}

## plot individual means
plot (plot.times, times, type="n", col="red", lwd=2, log="y",
      las=1, xlab="Time (week)", ylab="Plasma concentration (ng/ml)",
      main=paste("Study 3011. PP3M", dose_pp3m[j] ,"mg eq."),
      xlim=c(0, 66), ylim=c(1, 200),
      yaxp=c(1, 100, 1)) # ylim should be adapted!!!!

quant.p5 = apply(quant.data.p5.all, MAR=2, FUN=quantile, probs=c(0.05, 0.95))
lines(plot.times,  quant.p5[1,],  col="lightskyblue2")
lines(plot.times,  quant.p5[2,],  col="lightskyblue2")
polygon(c(plot.times,rev(plot.times)),
        c(quant.p5[2,],rev(quant.p5[1,])),
        col="lightskyblue2", border = "lightskyblue2")

quant.p50 = apply(quant.data.p50.all, MAR=2, FUN=quantile,
                  probs=c(0.05, 0.95))
lines(plot.times,  quant.p50[1,],  col="lightskyblue2")
lines(plot.times,  quant.p50[2,],  col="lightskyblue2")
polygon(c(plot.times,rev(plot.times)),
        c(quant.p50[2,],rev(quant.p50[1,])),
        col="lightskyblue2", border = "lightskyblue2")

quant.p95 = apply(quant.data.p95.all, MAR=2, FUN=quantile,
                  probs=c(0.05, 0.95))
lines(plot.times,  quant.p95[1,],  col="lightskyblue2")
lines(plot.times,  quant.p95[2,],  col="lightskyblue2")
polygon(c(plot.times,rev(plot.times)),
        c(quant.p95[2,],rev(quant.p95[1,])),
        col="lightskyblue2", border = "lightskyblue2")

if (j == 1) {
  lines(Magnus1$Time, Magnus1$P50, col="red",  lwd=2)
  lines(Magnus1$Time, Magnus1$P5,  col="red",  lty=2)
```

```
    lines(Magnus1$Time, Magnus1$P95, col="red",  lty=2)
  }
  if (j == 2) {
    lines(Magnus2$Time, Magnus2$P50, col="red",  lwd=2)
    lines(Magnus2$Time, Magnus2$P5,  col="red",  lty=2)
    lines(Magnus2$Time, Magnus2$P95, col="red",  lty=2)
  }
  if (j == 3) {
    lines(Magnus3$Time, Magnus3$P50, col="red",  lwd=2)
    lines(Magnus3$Time, Magnus3$P5,  col="red",  lty=2)
    lines(Magnus3$Time, Magnus3$P95, col="red",  lty=2)
  }
  if (j == 4) {
    lines(Magnus4$Time, Magnus4$P50, col="red",  lwd=2)
    lines(Magnus4$Time, Magnus4$P5,  col="red",  lty=2)
    lines(Magnus4$Time, Magnus4$P95, col="red",  lty=2)
  }
  abline(v=c(0, 14, 17, 29, 41, 53))
}

## End.
```

13.3 Partly Bayesian workflow implementation in R (v4)

```
## Partly Bayesian paliperidone palmitate VBE workflow
## With R:Nimble package
## v4

IDtag = "_4" # version number

## ==========================================================================
## 0. Read the statistical model and TOST test
source("Statistical model v13.R")
source("Our_TOST.R")
source("Cmax_AUC.R")

## ==========================================================================
## 1. Compile the model for prediction of an abbreviated BE trial

N.subjects.a = 25 # parallel trial, reference / test, 20 subjects per arm

## Magnusson dosings were at PP1M: 0, 4, 8, 12, PP3M: 17, 29, 41, 53 weeks.
## Magnusson sampling times were at:
##   1, 5, 9, 13, 14, 17(-),
##   21, 25, 29(-), 33, 37, 41(-), 45, 49, 53(-), 54, 55, 57, 61, 65
## (-) indicate "just before, say 1 hour before.
## We use the same times, but in hours.
Hr1 = 1 / (24 * 7) # one hour in weeks
times = c(1, 5, 9, 13, 14, 17-Hr1,
          21, 25, 29-Hr1, 33, 37, 41-Hr1, 45, 49, 53-Hr1, 54, 55, 57, 61, 65)
times = times * 24 * 7
plot.times = times / (24 * 7) # in weeks
N.times = length(plot.times)

dose_pp1m = 150
dose_pp3m = 525

Q_cen_0_mean = 30 # geo mean
Q_cen_0_sd   = 1.5  # geo SD

# order in Delta: f_3, k_as3,max, k_ar3,max, k_as3,50, k_ar3,50, Gamma
Delta = rep(1,6)

data  = list()
```

```
inits = list(Q_cen_0_mean = Q_cen_0_mean, # (mg)
             Q_cen_0_sd   = Q_cen_0_sd,   # (mg)
             Dose_PP1M    = dose_pp1m[1], # (mg)
             Dose_PP3M    = dose_pp3m[1], # (mg)
             Delta        = Delta)

constants = list(nsubjects_per_arm = N.subjects.a,
                 Do_fit            = 0,            # 0: no fit, > 0: index
                 SamEq3            = 1,            # Boolean
                 Duration_2        = 319,          # (h)
                 PP3M_start        = 17*7*24,      # (h)
                 ntimes            = N.times,
                 times             = times,
                 nstates           = 4)

Rmodel = nimbleModel(myNimbleCode, constants, data, inits, calculate=F)
Cmodel = compileNimble(Rmodel, showCompilerOutput=F)

## =========================================================================
## 2a. Simulate an abbreviated BE trial, assuming bioequivalence

## Simulate the whole trial at once
Node.names = Cmodel$getNodeNames(includeData=T)
Cmodel$Delta[2] = 1.05 # ka slow max
Cmodel$simulate(nodes = Node.names)
all.pred = values(Cmodel, "Conc")          # individual profiles no noise
all.data = values(Cmodel, "C_plasma_obs") # for just the simulated data

all.pred = matrix(all.pred, ncol=N.times, byrow = F) # subjects by row
all.data = matrix(all.data, ncol=N.times, byrow = F) # subjects by row

## Reference arm of the trial
ref.pred.mat = all.pred[1:N.subjects.a,]
ref.data.mat = all.data[1:N.subjects.a,]

## Test arm of the trial
test.pred.mat = all.pred[(N.subjects.a + 1):(2 * N.subjects.a),]
test.data.mat = all.data[(N.subjects.a + 1):(2 * N.subjects.a),]

# parameters
param.pop = values(Cmodel,
                   c("ka1_pp1m_m", "ka1_max_m", "ka3_max_m",
                     "kamt1_50_m", "kamt3_50_m", "gamma_m", "CL_m",
                     "F2_v", "ka1_pp1m_v", "ka1_max_v", "kamt1_50_v",
                     "kamt3_50_v", "CL_v", "sigma2"))
param.ind =  values(Cmodel,
                    c("F2", "F3", "ka_PP1M","ka1_max", "ka3_max",
                      "kamt1_50", "kamt3_50", "gamma", "CL", "V"))

param.ind.mat = matrix(param.ind, nrow=N.subjects.a, byrow = F)

colnames(param.ind.mat) = c("F2_Ref", "F2_Test", "F3_Ref", "F3_Test",
                            "ka_PP1M_Ref","ka_PP1M_Test",
                            "ka1_max_Ref", "ka1_max_Test",
                            "ka3_max_Ref","ka3_max_Test",
                            "kamt1_50_Ref", "kamt1_50_Test",
                            "kamt3_50_Ref", "kamt3_50_Test",
                            "gamma_Ref", "gamma_Test", "CL_Ref",
                            "CL_Test", "V_Ref", "V_Test")

rownames(param.ind.mat) = c(paste0("subject_", 1:N.subjects.a))

param.pop.mat = matrix(param.pop, nrow=1, byrow = F)
```

```
colnames(param.pop.mat) = c("ka1_pp1m_m", "ka1_max_m", "ka3_max_m",
                            "kamt1_50_m", "kamt3_50_m", "gamma_m", "CL_m",
                            "F2_v", "ka1_pp1m_v", "ka1_max_v",
                            "kamt1_50_v", "kamt3_50_v", "CL_v", "sigma2")

## Check
plot(as.numeric(ref.pred.mat), as.numeric(ref.data.mat),
     type ="p", col="blue",lwd=0.5, log="xy")
plot(plot.times, ref.pred.mat[1,],  type ="l", col="blue",lwd=0.5)
points(plot.times, ref.data.mat[1,], col="blue",lwd=0.5)

## Plot
plot(plot.times, plot.times, type="n", xlab="Time (weeks)",
     ylab="Plasma concentration (ng/ml)", yaxt ="n",
     ylim=c(1,1000), log="y",cex.lab=1.3)
axis(2, at = c(0.001,0.01, 0.1, 1, 10, 100, 1000),
     labels=c("0.001","0.01", "0.1","1", "10", "100", "1000"), las=1)
##
for (i in 1:N.subjects.a) {
  lines(plot.times, ref.data.mat[i,],  type="b", col="blue")
  lines(plot.times, test.data.mat[i,], type="b", col="red")
}
abline(v=c(0, 4, 8, 12, 17, 29, 41, 53))
legend(x=45, y=1000, leg=c("Reference", "Test"), lty=1,
       col=c("blue", "red"), bty="o", bg="white", box.lty=0)

bSave = FALSE
if (bSave) {
  ## Save simulated data
  names.col = c("time in h", paste0("subject_Ref_", 1:N.subjects.a),
                paste0("subject_Test_", 1:N.subjects.a))
  write.table(cbind(t(t(times)),t(ref.data.mat),t(test.data.mat)),
              file = paste0("AbbreviatedTrial_25subjectsPerArm_data", IDtag,
                            ".csv"),
              sep = ",", dec = ".", row.names = FALSE, col.names = names.col)

  ## Save mean individual profiles
  write.table(cbind(t(t(times)),t(ref.pred.mat),t(test.pred.mat)),
              file = paste0("AbbreviatedTrial_25subjectsPerArm_pred", IDtag,
                            ".csv"),
              sep = ",", dec = ".", row.names = FALSE, col.names = names.col)

  ## Save parameters
  write.table(param.ind.mat,
              file = paste0("AbbreviatedTrial_25subjectsPerArm_param_ind",
                            IDtag, ".csv"),
              sep=",", dec=".",
              row.names=c(paste0("subject_", 1:N.subjects.a)))
  write.table(param.pop.mat,
              file = paste0("AbbreviatedTrial_25subjectsPerArm_param_pop",
                            IDtag, ".csv"),
              sep=",", dec=".", row.names=FALSE)
}

## ==========================================================================
## 2b. Alternative: read the abbreviated trial data

ab.data = read.csv("AbbreviatedTrial_25subjectsPerArm_data_4.csv")
dim(ab.data)

N.times      =  dim(ab.data)[1]
N.subjects.a = (dim(ab.data)[2] - 1) / 2 # parallel trial subjects per arm

plot.times = ab.data$time.in.h / (24 * 7) # in weeks
```

```
## Compute Cmax
istart = 16
iend   = N.times
itime  = istart:iend
iref   = 1:N.subjects.a + 1
itest  = (N.subjects.a + 1):(2 * N.subjects.a) + 1
Cmax.ref  = get.Cmax(ab.data[itime,iref])
Cmax.test = get.Cmax(ab.data[itime,itest])

## Compute AUC for each subject in the last dosing period (using the last 5
## time points for each subject)
AUC.ref  = get.AUC(plot.times[itime], ab.data[itime,iref])
AUC.test = get.AUC(plot.times[itime], ab.data[itime,itest])

## Plot concentrations, Cmax, AUC
pdf("Abbreviated trial plot.pdf")
layout(matrix(1:4,1,4), widths=c(0.8,0.1,0.1,0.01))
par(mar=c(5,5,15,0))
plot(plot.times, plot.times, type="n", xlab="Time (weeks)",
     ylab="Plasma concentration (ng/ml)", yaxt ="n",
     ylim=c(1,1000), log="y", cex.lab=1.5)
axis(2, at = c(0.001,0.01, 0.1, 1, 10, 100, 1000),
     labels=c("0.001","0.01", "0.1","1", "10", "100", "1000"), las=1)
##
for (i in 1:N.subjects.a) {
  lines(plot.times, ab.data[,i+1], type="b", col="blue") # ref
}
for (i in (N.subjects.a + 1):(2 * N.subjects.a)) {
  lines(plot.times, ab.data[,i+1], type="b", col="red")  # test
}
abline(v=c(0, 4, 8, 12, 17, 29, 41, 53), col="gray")
legend(x=45, y=1000, leg=c("Reference", "Test"), lty=1,
       col=c("blue", "red"), bty="o", bg="white", box.lty=0)
##
## Plot Cmax
par(mar=c(5,0,15,0))
boxplot(Cmax.ref, Cmax.test, xlim=c(0.5,2.5), ylim=c(1,1000), log="y",
        col=c("royalblue", "violetred1"),
        xaxt="s", yaxt="n", xlab="", ylab="", names=c("Ref", "Test"))
text(1.5, y=1000, lab="Cmax")
##
## Plot AUC divided by time difference (to be on a concentration scale)
diffT = plot.times[iend] - plot.times[istart]
boxplot(AUC.ref/diffT, AUC.test/diffT, xlim=c(0.5,2.5),
        ylim=c(1,1000), log="y", col=c("royalblue", "violetred1"),
        yaxt="n", xlab="", ylab="", names=c("Ref", "Test"))
text(1.5, y=1000, lab=expression(AUC / Delta[t]))
dev.off()

## Perform a standard BE test (two one-sided t-test) on trial results:
Cmax.yes = myTOST(Cmax.ref, Cmax.test)
AUC.yes  = myTOST(AUC.ref, AUC.test)
BE.yes   = Cmax.yes && AUC.yes
## all FALSE

## Cmax geometric means ratio
exp(mean(log(Cmax.test)) - mean(log(Cmax.ref)))

## Cmax CV
mean(c(sd(Cmax.ref) / mean(Cmax.ref), sd(Cmax.test) / mean(Cmax.test)))

## AUC geometric means ratio
exp(mean(log(AUC.test)) - mean(log(AUC.ref)))
```

```
## AUC CV
mean(c(sd(AUC.ref) / mean(AUC.ref), sd(AUC.test) / mean(AUC.test)))

## Remove columns names
colnames(ab.data) = NULL


## ==========================================================================
## 3a. Bayesian calibration of the PP3M model given abbreviated trial data.

## Parallelize
library(parallel)
N.cores = detectCores() / 2
this_cluster <- makeCluster(N.cores)

## Create a function with all the needed code
run_MCMC_allcode <- function(seed) {
  ##
  library(nimble)
  source("Statistical model v13.R")
  ##
  ab.data = read.csv("AbbreviatedTrial_25subjectsPerArm_data_4.csv")
  N.subjects.a = (dim(ab.data)[2] - 1) / 2 # parallel trial subjects per arm
  ## Remove columns names
  colnames(ab.data) = NULL
  ##
  Hr1 = 1 / (24 * 7) # one hour in weeks
  times = c(1, 5, 9, 13, 14, 17-Hr1,
            21, 25, 29-Hr1, 33, 37, 41-Hr1, 45, 49, 53-Hr1, 54, 55, 57, 61, 65)
  times = times * 24 * 7
  plot.times = times / (24 * 7) # in weeks
  N.times = length(plot.times)
  ##
  dose_pp1m = 150
  dose_pp3m = 525
  ##
  Q_cen_0_mean = 30 # geo mean
  Q_cen_0_sd   = 1.5  # geo SD
  ##
  Delta = rep(1,6)
  ##
  data  = list(C_plasma_obs=t(ab.data[,-1]))
  ##
  inits = list(Q_cen_0_mean = Q_cen_0_mean, # (mg)
               Q_cen_0_sd   = Q_cen_0_sd,   # (mg)
               Dose_PP1M    = dose_pp1m[1], # (mg)
               Dose_PP3M    = dose_pp3m[1], # (mg)
               Delta        = Delta)
  ##
  constants = list(nsubjects_per_arm = N.subjects.a,
                   Do_fit = 1,    ## 0: no Delta fit, > 0: fit
                   logmeanD2  = 0,
                   logvarD2   = log(2)^2,
                   SamEq3     = 1,            # Boolean, leave at 1
                   Duration_2 = 319,          # (h)
                   PP3M_start = 17*7*24,      # (h)
                   ntimes     = length(times),
                   times      = times,
                   nstates    = 4)
  ##
  Rmodel = nimbleModel(myNimbleCode, constants, data, inits, calculate=F)
  ##
  conf = configureMCMC(Rmodel, thin=1, # useConjugacy = FALSE,
                       nodes=c("logDelta2",
                               "tmp2", "tmp3", "ka_PP1M", "ka1_max",
```

```
                              "kamt1_50", "kamt3_50", "CL", "V", "Q_cen_0"),
                       monitors=c("logDelta2",
                                  "F2", "F3", "ka_PP1M", "ka1_max",
                                  "kamt1_50", "kamt3_50", "CL", "V", "Q_cen_0",
                                  "Conc",
                                  "logProb_logDelta2",
                                  "logProb_tmp2", "logProb_tmp3",
                                  "logProb_ka_PP1M", "logProb_ka1_max",
                                  "logProb_kamt1_50", "logProb_kamt3_50",
                                  "logProb_CL", "logProb_V", "logProb_Q_cen_0",
                                  "logProb_C_plasma_obs"))
  ##
  Rmcmc  = buildMCMC(conf)
  Cmodel = compileNimble(Rmodel, showCompilerOutput=F)
  Cmcmc  = compileNimble(Rmcmc, project=Rmodel)
  ##
  Cmodel$ka1_pp1m_m  = 4.88E-4
  Cmodel$ka1_max_m   = 0.0904
  Cmodel$ka3_max_m   = 0.164
  Cmodel$kamt1_50_m  = 120
  Cmodel$kamt3_50_m  = 21.4
  Cmodel$gamma_m     = 1.44
  Cmodel$CL_m        = 3.84
  Cmodel$F2_v        = (0.064 / (0.168 * (1 - 0.168)))^2
  Cmodel$F3_v        = (0.854 / (1 - 0.209))^2
  Cmodel$ka1_pp1m_v  = log(1 + 0.590^2)
  Cmodel$ka1_max_v   = log(1 + 0.827^2)
  Cmodel$kamt1_50_v  = log(1 + 0.5^2)
  Cmodel$kamt3_50_v  = log(1 + 0.867^2)
  Cmodel$CL_v        = log(1 + 0.357^2)
  Cmodel$res_cv      = 0.306
  ##
  mysamples = runMCMC(Cmcmc, niter=10000, nburnin=2500, setSeed=seed)
  return(mysamples)
  ##
} ## End run_MCMC_allcode

chain_output <- parLapply(cl=this_cluster, X=1:N.cores, fun=run_MCMC_allcode)

save(chain_output,
     file=paste0("Parallel chains output.Delta2.fix pop",IDtag,".Rsave"))
## load(file=paste0("Parallel chains output.Delta2.fix pop",IDtag,".Rsave"))

pdf("Parallel chains trajectories.IWish.fix pop.pdf")
par(mfrow = c(1,1))
mycolors = rainbow(N.cores)
for (j in 1:dim(chain_output[[1]])[2]) {
  for (i in 1:N.cores) {
    this_output <- chain_output[[i]]
    if (i == 1) {
      plot(this_output[,j], type = "l", ylab = colnames(this_output)[j],
           col=mycolors[i], las=1)
    } else {
      lines(this_output[,j], col=mycolors[i])
    }
  }
}
dev.off()

## Close the cluster when you're done with it.
stopCluster(this_cluster)

## ====================================================================
## 3b. Check the model using the abbreviated BE trial
```

```
## There is no point of doing that with simulations of the prior model,
## unless we want to exercise the checking tools.
## For now, we just show fit plots.

## ========================================================================
## 4. Simulate a virtual parallel bioequivalence trials with many subjects

N.subjects.v = 130 # number of virtual subjects per arm

Hr1 = 1 / (24 * 7) # one hour in weeks
times = c(1, 5, 9, 13, 14, 17-Hr1,
          21, 25, 29-Hr1, 33, 37, 41-Hr1, 45, 49, 53-Hr1, 54, 55, 57, 61, 65)
times = times * 24 * 7
plot.times = times / (24 * 7) # in weeks
N.times = length(plot.times)

dose_pp1m = 150
dose_pp3m = 525

Q_cen_0_mean = 30 # geo mean
Q_cen_0_sd   = 1.5  # geo SD

Delta = rep(1,6)

data  = list()

inits = list(Q_cen_0_mean = Q_cen_0_mean, # (mg)
             Q_cen_0_sd   = Q_cen_0_sd,   # (mg)
             Dose_PP1M    = dose_pp1m[1], # (mg)
             Dose_PP3M    = dose_pp3m[1], # (mg)
             Delta        = Delta)

constants = list(nsubjects_per_arm = N.subjects.v,
                 Do_fit            = 0,            # 0: no fit, > 0: index
                 SamEq3            = 1,            # Boolean
                 Duration_2        = 319,          # (h)
                 PP3M_start        = 17*7*24,      # (h)
                 ntimes            = N.times,
                 times             = times,
                 nstates           = 4)

Rmodel = nimbleModel(myNimbleCode, constants, data, inits, calculate=F)
Cmodel = Rmodel
Cmodel = compileNimble(Rmodel, showCompilerOutput=F)

Cmodel$Delta[2]    = 1.42 # posterior mean
Cmodel$ka1_pp1m_m  = 4.88E-4
Cmodel$ka1_max_m   = 0.0904
Cmodel$ka3_max_m   = 0.164
Cmodel$kamt1_50_m  = 120
Cmodel$kamt3_50_m  = 21.4
Cmodel$gamma_m     = 1.44
Cmodel$CL_m        = 3.84
Cmodel$F2_v        = (0.064 / (0.168 * (1 - 0.168)))^2
Cmodel$F3_v        = (0.854 / (1 - 0.209))^2
Cmodel$ka1_pp1m_v  = log(1 + 0.590^2)
Cmodel$ka1_max_v   = log(1 + 0.827^2)
Cmodel$kamt1_50_v  = log(1 + 0.5^2)
Cmodel$kamt3_50_v  = log(1 + 0.867^2)
Cmodel$CL_v        = log(1 + 0.357^2)
Cmodel$res_cv      = 0.306

## Run a BE trial
Node.names = Cmodel$getNodeNames(includeData=T)
Cmodel$simulate(nodes = Node.names)
```

```
all.res  = values(Cmodel, Node.names)
all.data = values(Cmodel, "C_plasma_obs") # for just the simulated data
all.pred = values(Cmodel, "Conc") # for just the individual profile no noise

all.data = matrix(all.data, ncol=N.times, byrow = F) # subjects by row
all.pred = matrix(all.pred, ncol=N.times, byrow = F) # subjects by row

## Reference arm of the trial
ref.data.mat = all.data[1:N.subjects.v,]
ref.pred.mat = all.pred[1:N.subjects.v,]

## Test arm of the trial
test.data.mat = all.data[(N.subjects.v + 1):(2 * N.subjects.v),]
test.pred.mat = all.pred[(N.subjects.v + 1):(2 * N.subjects.v),]

## Compute Cmax
istart = 16
iend   = N.times
itime  = istart:iend
isub   = 1:N.subjects.v
Cmax.ref  = get.Cmax(t(ref.data.mat[ ,itime]))
Cmax.test = get.Cmax(t(test.data.mat[,itime]))

## Compute AUC for each subject in the last dosing period
AUC.ref  = get.AUC(plot.times[itime], t(ref.data.mat[ ,itime]))
AUC.test = get.AUC(plot.times[itime], t(test.data.mat[,itime]))

## Plot concentrations, Cmax, AUC
pdf("Virtual trial N 130 simulated profiles.pdf")
layout(matrix(1:4,1,4), widths=c(0.8,0.1,0.1,0.01))
par(mar=c(5,5,15,0))
plot(plot.times, plot.times, type="n", xlab="Time (weeks)",
     ylab="Plasma concentration (ng/ml)", yaxt ="n",
     ylim=c(1,1000), log="y", cex.lab=1.5)
axis(2, at = c(0.001,0.01, 0.1, 1, 10, 100, 1000),
     labels=c("0.001","0.01", "0.1","1", "10", "100", "1000"), las=1)
##
for (i in 1:N.subjects.v) {
  lines(plot.times, ref.data.mat[ i,], type="b", col="blue") # ref
  lines(plot.times, test.data.mat[i,], type="b", col="red")  # test
}
abline(v=c(0, 4, 8, 12, 17, 29, 41, 53), col="gray")
legend(x=45, y=1000, leg=c("Reference", "Test"), lty=1,
       col=c("blue", "red"), bty="o", bg="white", box.lty=0)
##
## Plot Cmax
par(mar=c(5,0,15,0))
boxplot(Cmax.ref, Cmax.test, xlim=c(0.5,2.5), ylim=c(1,1000), log="y",
        col=c("royalblue", "violetred1"),
        xaxt="s", yaxt="n", xlab="", ylab="", names=c("Ref", "Test"))
text(1.5, y=1000, lab="Cmax")
##
## Plot AUC divided by time difference (to be on a concentration scale)
diffT = plot.times[iend] - plot.times[istart]
boxplot(AUC.ref/diffT, AUC.test/diffT, xlim=c(0.5,2.5),
        ylim=c(1,1000), log="y", col=c("royalblue", "violetred1"),
        yaxt="n", xlab="", ylab="", names=c("Ref", "Test"))
text(1.5, y=1000, lab=expression(AUC / Delta[t]))
dev.off()

## ======================================================================
## 5. Perform a standard BE test (two one-sided t-test) on trial results,

Cmax.yes = myTOST(Cmax.ref, Cmax.test)
```

```
AUC.yes  = myTOST(AUC.ref, AUC.test)
BE.yes   = Cmax.yes && AUC.yes

## Cmax geometric means ratio
exp(mean(log(Cmax.test)) - mean(log(Cmax.ref)))

## Cmax CV
mean(c(sd(Cmax.ref) / mean(Cmax.ref), sd(Cmax.test) / mean(Cmax.test)))

## AUC geometric means ratio
exp(mean(log(AUC.test)) - mean(log(AUC.ref)))

## AUC CV
mean(c(sd(AUC.ref) / mean(AUC.ref), sd(AUC.test) / mean(AUC.test)))

## ===========================================================================
## 6. Power: probability of declaring BE when it is true.
## This is equal to (1 - type II error), where type II error is the
## probability of rejecting BE when it is true
## Compute once for many subjects and then use only part of the data
## for smaller trial sizes.

N.subjects.v = 500 # number of virtual subjects per arm

Hr1 = 1 / (24 * 7) # one hour in weeks
times = c(1, 5, 9, 13, 14, 17-Hr1,
          21, 25, 29-Hr1, 33, 37, 41-Hr1, 45, 49, 53-Hr1, 54, 55, 57, 61, 65)
times = times * 24 * 7
plot.times = times / (24 * 7) # in weeks
N.times = length(plot.times)

dose_pp1m = 150
dose_pp3m = 525

Q_cen_0_mean = 30   # geo mean
Q_cen_0_sd   = 1.5  # geo SD

## We need to recompile, because nsubjects must be a constant
constants = list(nsubjects_per_arm = N.subjects.v,
                 Do_fit            = 0,            # 0: no fit, > 0: index
                 SamEq3            = 1,            # Boolean
                 Duration_2        = 319,          # (h)
                 PP3M_start        = 17*7*24,      # (h)
                 ntimes            = N.times,
                 times             = times,
                 nstates           = 4)

data  = list()

inits = list(Q_cen_0_mean = Q_cen_0_mean, # (mg)
             Q_cen_0_sd   = Q_cen_0_sd,   # (mg)
             Dose_PP1M    = dose_pp1m[1], # (mg)
             Dose_PP3M    = dose_pp3m[1], # (mg)
             Delta        = rep(1, 6))

Rmodel = nimbleModel(myNimbleCode, constants, data, inits, calculate=F)
Cmodel = Rmodel
Cmodel = compileNimble(Rmodel, showCompilerOutput=F)

Node.names = Cmodel$getNodeNames(includeData=T)
Cmodel$simulate(nodes = Node.names)
res = values(Cmodel, Node.names)

## Main simulation: bioequivalent case
Cmodel$Delta = c(1, 1, 1, 1, 1, 1) # true BE
```

```
N.mtc.v = 1000 # number of simulated virtual trials
BE.yes = rep(-1, N.mtc.v)
Cmax.ref = Cmax.test = matrix(0, nrow=N.mtc.v, ncol=N.subjects.v)
AUC.ref  = AUC.test  = matrix(0, nrow=N.mtc.v, ncol=N.subjects.v)
istart   = 16
iend     = N.times
itime    = istart:iend
for (i in 1:N.mtc.v) {
  ## Simulate the trial
  Cmodel$simulate(nodes = Node.names)
  all.data = values(Cmodel, "C_plasma_obs")
  all.data = matrix(all.data, ncol=N.times, byrow = F) # subjects by row
  ##
  ## Reference arm of the trial
  ref.data.mat = all.data[1:N.subjects.v,]
  ##
  ## Test arm of the trial
  test.data.mat = all.data[(N.subjects.v + 1):(2 * N.subjects.v),]
  ##
  ## find Cmax for each subject, record it
  Cmax.ref[i,]  = get.Cmax(t(ref.data.mat[ ,itime]))
  Cmax.test[i,] = get.Cmax(t(test.data.mat[,itime]))
  ##
  ## find AUC for each subject, record it
  AUC.ref[i,]  = get.AUC(plot.times[itime], t(ref.data.mat[, itime]))
  AUC.test[i,] = get.AUC(plot.times[itime], t(test.data.mat[,itime]))
  ##
  print(paste("Trial", i))
}

save(list=c("N.mtc.v", "N.subjects.v", "Cmax.ref", "Cmax.test", "AUC.ref",
            "AUC.test"), file=paste0("Cmax & AUC for power",IDtag,".Rsave"))
## load(file=paste0("Cmax & AUC for power",IDtag,".Rsave"))

## Now process increasing chunks of the results
power = rep(0, N.subjects.v)
BE.yes = rep(0, N.mtc.v)
for (k in 2:N.subjects.v) {
  N.subj.current = k
  my.index = sample.int(N.subjects.v, N.subj.current)
  for (i in 1:N.mtc.v) {
    Cmax.yes = myTOST(Cmax.ref[i,my.index], Cmax.test[i,my.index])
    AUC.yes  = myTOST(AUC.ref[i,my.index], AUC.test[i,my.index])
    #
    BE.yes[i] = Cmax.yes && AUC.yes
  }
  ## power:
  power[k] = sum(BE.yes) / N.mtc.v # probability of declaring BE
}

## Plot
pdf("Power plot after calibration.pdf")
plot(2:N.subjects.v, power[-1], type="l", xlab="Number of subjects per arm",
     ylab="Probability of declaring bioequivalence", xlim=c(1,N.subjects.v),
     ylim=c(0, 1), col="grey", lwd=0.5, las=1)
lines(2:N.subjects.v, supsmu(2:N.subjects.v, power[-1])$y, lwd=2, col="red")
dev.off()

## =========================================================================
## 7. Type I error: probability of declaring BE when it is not true.

## Compute once for many subjects and then use only part of the data
## for smaller trial sizes.
```

```
N.subjects.v = 500 # number of virtual subjects per arm

Hr1 = 1 / (24 * 7) # one hour in weeks
times = c(1, 5, 9, 13, 14, 17-Hr1,
          21, 25, 29-Hr1, 33, 37, 41-Hr1, 45, 49, 53-Hr1, 54, 55, 57, 61, 65)
times = times * 24 * 7
plot.times = times / (24 * 7) # in weeks
N.times = length(plot.times)

dose_pp1m = 150
dose_pp3m = 525

Q_cen_0_mean = 30   # geo mean
Q_cen_0_sd   = 1.5  # geo SD

## We need to recompile, because nsubjects must be a constant
constants = list(nsubjects_per_arm = N.subjects.v,
                 Do_fit            = 0,            # 0: no fit, > 0: index
                 SamEq3            = 1,            # Boolean
                 Duration_2        = 319,          # (h)
                 PP3M_start        = 17*7*24,      # (h)
                 ntimes            = N.times,
                 times             = times,
                 nstates           = 4)

data  = list()

inits = list(Q_cen_0_mean = Q_cen_0_mean, # (mg)
             Q_cen_0_sd   = Q_cen_0_sd,   # (mg)
             Dose_PP1M    = dose_pp1m[1], # (mg)
             Dose_PP3M    = dose_pp3m[1], # (mg)
             Delta        = rep(1, 6))

Rmodel = nimbleModel(myNimbleCode, constants, data, inits, calculate=F)
Cmodel = compileNimble(Rmodel, showCompilerOutput=F)

Node.names = Cmodel$getNodeNames(includeData=T)
Cmodel$simulate(nodes = Node.names)
res = values(Cmodel, Node.names)

## Main simulation
N.mtc.v = 1000 # number of simulated virtual trials
BE.yes = rep(-1, N.mtc.v)
Delta    = matrix(0, nrow=N.mtc.v, ncol=6)
Cmax.ref = Cmax.test = matrix(0, nrow=N.mtc.v, ncol=N.subjects.v)
AUC.ref  = AUC.test  = matrix(0, nrow=N.mtc.v, ncol=N.subjects.v)
istart   = 16
iend     = N.times
itime    = istart:iend
t.start = Sys.time()
for (i in 1:N.mtc.v) {
  ## Simulate the trial
  Delta[i,]    = runif(6, 0.7, 1.5)
  Cmodel$Delta = Delta[i,]
  Cmodel$simulate(nodes = Node.names)
  all.data = values(Cmodel, "C_plasma_obs")
  all.data = matrix(all.data, ncol=N.times, byrow = F) # subjects by row
  ##
  ## Reference arm of the trial
  ref.data.mat = all.data[1:N.subjects.v,]
  ##
  ## Test arm of the trial
  test.data.mat = all.data[(N.subjects.v + 1):(2 * N.subjects.v),]
  ##
```

```
  ## find Cmax for each subject, record it
  Cmax.ref[i,]  = get.Cmax(t(ref.data.mat[ ,16:N.times]))
  Cmax.test[i,] = get.Cmax(t(test.data.mat[,16:N.times]))
  ##
  ## find AUC for each subject, record it
  AUC.ref[i,]  = get.AUC(plot.times[itime], t(ref.data.mat[, itime]))
  AUC.test[i,] = get.AUC(plot.times[itime], t(test.data.mat[,itime]))
  ##
  print(paste("Trial", i))
}
t.end = Sys.time()
t.end - t.start
## 98% of the time is spend simulating trials

## Save
save(list=c("N.mtc.v", "N.subjects.v", "Delta",
            "Cmax.ref", "Cmax.test", "AUC.ref", "AUC.test"),
     file=paste0("Cmax & AUC for type 1 error", IDtag,".Rsave"))
## load(file=paste0("Cmax & AUC for type 1 error",IDtag,".Rsave"))

## Set number of subjects to use; we can use at most N.subjects.v subjects
N.subject.used = N.subjects.v
j = 1:N.subject.used

## Cmax difference per trial
Cmax.ref.means  = apply(log(Cmax.ref[,j]),  MARGIN=1, FUN=mean)
Cmax.test.means = apply(log(Cmax.test[,j]), MARGIN=1, FUN=mean)
## Test/Ref relative differences
Cmax.rel.diffs = exp(Cmax.test.means - Cmax.ref.means)
mean(Cmax.rel.diffs)

## AUC difference per trial
AUC.ref.means  = apply(log(AUC.ref[,j]),  MARGIN=1, FUN=mean)
AUC.test.means = apply(log(AUC.test[,j]), MARGIN=1, FUN=mean)
## Test/Ref relative differences
AUC.rel.diffs = exp(AUC.test.means - AUC.ref.means)
mean(AUC.rel.diffs)

## Compute BE for the various trials
Cmax.yes = AUC.yes = BE.yes = rep(0, N.mtc.v)
for (i in 1:N.mtc.v) {
  Cmax.yes[i] = myTOST(Cmax.ref[i,], Cmax.test[i,])
  AUC.yes[i]  = myTOST(AUC.ref[i,], AUC.test[i,])
  BE.yes[i]   = Cmax.yes[i] && AUC.yes[i]
}

## Plot passes
pdf("Type 1 error plot.pdf")
par(mar=c(8,5,7,1), las=1, cex.lab=1.2)
plot(Cmax.rel.diffs, Cmax.yes, type="p",
     xlab="Cmax relative differences geometric mean",
     ylab="Probability of declaring Cmax BE")
abline(v=c(0.8, 1.25), col="red")
plot(AUC.rel.diffs, AUC.yes, type="p",
     xlab="AUC relative differences geometric mean",
     ylab="Probability of declaring AUC BE")
abline(v=c(0.8, 1.25), col="red")
## lines(AUC.centers, AUC.error1, col="blue")
dev.off()

## ====================================================================
## 8. Safe space: probability of declaring BE when it is not true.
##    We have saved the Delta and BE decisions in the type 1 error
##    calculations; we can just reuse them.
```

```
## The saved simulation are the same as type 1 error but a copy has been made.
## load(file=paste0("Cmax & AUC for type 1 error",IDtag,".Rsave"))
load(file=paste0("Cmax & AUC for safe space",IDtag,".Rsave"))

## Set number of subjects to use; we can use at most N.subjects.v subjects
N.subject.used = N.subjects.v
j = 1:N.subject.used

## Cmax difference per trial
Cmax.ref.means  = apply(log(Cmax.ref[,j]),  MARGIN=1, FUN=mean)
Cmax.test.means = apply(log(Cmax.test[,j]), MARGIN=1, FUN=mean)
## Test/Ref relative differences
Cmax.rel.diffs = exp(Cmax.test.means - Cmax.ref.means)
mean(Cmax.rel.diffs)

## AUC difference per trial
AUC.ref.means  = apply(log(AUC.ref[,j]),  MARGIN=1, FUN=mean)
AUC.test.means = apply(log(AUC.test[,j]), MARGIN=1, FUN=mean)
## Test/Ref relative differences
AUC.rel.diffs = exp(AUC.test.means - AUC.ref.means)
mean(AUC.rel.diffs)

## Compute BE for the various trials
Cmax.yes = AUC.yes = BE.yes = rep(0, N.mtc.v)
for (i in 1:N.mtc.v) {
  Cmax.yes[i] = myTOST(Cmax.ref[i,], Cmax.test[i,])
  AUC.yes[i]  = myTOST(AUC.ref[i,], AUC.test[i,])
  BE.yes[i]   = Cmax.yes[i] && AUC.yes[i]
}

## Plot safe space
pdf("Safe space plot.pdf")
is.BE = which(BE.yes == 1) # we could also use Cmax.yes or AUC.yes
my.panel = function (x,y) {
  par(las=1, cex.lab=1.2)
  points(x[ is.BE], y[ is.BE], pch=16, cex=0.3, col="green")
  points(x[-is.BE], y[-is.BE], pch=16, cex=0.3, col="red")
}
my.labels = c(expression(delta[1]), expression(delta[2]), expression(delta[3]),
              expression(delta[4]), expression(delta[5]), expression(delta[6]))
pairs(Delta, panel=my.panel, labels=my.labels)
dev.off()

## End.
```

13.4 Statistical model in R (v13)

```
## R/Nimble code for PP1M/PP3M population model, from Magnusson
## v13

library(nimble)

## compile C ODE model for deSolve
Cmodel.name = "PP13M_model_v04"
## system(paste0("R CMD SHLIB ", Cmodel.name, ".c"))
dyn.load(paste(Cmodel.name, .Platform$dynlib.ext, sep = ""))

## ==========================================================================
## Define the model

## --------------------------------------------------------------------------
## ODE solver: performs a simulation, given initial state values,
## output times and time-constant, scaled, parameters
## This is an R function
```

```
R_ode = function(y, times, parms) {
  ## parms: 1:Dose_PP1M, 2:F2,         3:Duration_2,  4:ka_PP1M,
  ##        5:ka1_max,   6:ka3_max,    7:kamt1_50,    8:kamt3_50,
  ##        9:gamma,    10:CL,        11:V,          12:PP3M_start,
  ##       13:F3,       14:Dose_PP3M, 15:Q_cen_0
  ## The last two parameters should not be passed to the ODE solver, they are
  ## used here only.
  ## State variables (y) initial conditions
  y = c("Q_depot_s1" = 0,
        "Q_depot_s3" = 0,
        "Q_depot_r3" = 0,
        "Q_central"  = parms[15])
  ## The doses are specified as "events" affecting the state variables
  ## dosing times (hours)
  dose_times = c(c(0, 4, 8, 12) + parms[3] / (24 * 7),
               17, 29, 41, 53) * 24 * 7
  N_doses = length(dose_times)
  ## changing state variables at dosing times
  vars = c("Q_depot_s1", "Q_depot_s3", "Q_depot_r3")
  v1 = (1 -  parms[2])  * parms[1]     # value applied to Q_depot_s1
  v2 = (1 -  parms[13]) * parms[14]    # value applied to Q_depot_s3
  v3 =       parms[13]  * parms[14]    # value applied to Q_depot_r3
  ## Form the events table
  eventdat = data.frame(var = c(rep("Q_depot_s1", 4), rep(vars[2:3], 4)),
                      time = c(dose_times[1:4], rep(dose_times[5:8], each=2)),
                      value = c(rep(v1, 4),
                                rep(c(v2, v3), N_doses - 4)),
                      method = "add")
  ## Integrate numerically, with outputs at specified times
  result = deSolve::lsode(y, c(0,times), func="derivs", parms=parms[1:12],
                        rtol=1e-6, atol=1e-6, dllname="PP13M_model_v04",
                        initfunc = "PP13M_model_v04",
                        events=list(data=eventdat))
  result = result[which(result[,1] %in% times),] # weed out extra times
  if (dim(result)[[1]] < length(times)) { ## integration failed
    return(rep(1E-30, length(times)))
  } else {
    ## compute central concentration, convert from mg/L to ng/ml, return
    return(result[,5] * 1E3 / parms[11])
  }
} # end of R_ode model solver

## ---------------------------------------------------------------------------
## Nimble function with nimbleRcall. This is just a wrapper.
nimble_ode = nimbleRcall(
  prototype = function(
    y     = double(1), # vector
    times = double(1), # vector
    parms = double(1)  # vector
  ) {},
  returnType = double(1), # outcome is a vector
  Rfun = 'R_ode'
)

## ---------------------------------------------------------------------------
## Hierarchical core Nimble (BUGS) code
myNimbleCode = nimbleCode({ ## BUGS (extended) code
  ##
  ## REFERENCE group population mean (with prior if not fixed)
  F2t_m       <- logit(0.168) # From Samtani paper Table III.
  F3t_m       <- logit(0.209) # transformed F3
  ka1_pp1m_mv <- log(1 + 0.02^2)
  ka1_pp1m_m  ~  dlnorm(meanlog=log(4.88E-4), varlog=ka1_pp1m_mv) # (1/h)
  ka1_max_mv  <- log(1 + 0.0696^2)
```

```
ka1_max_m   ~  dlnorm(meanlog=log(0.0904),  varlog=ka1_max_mv)  # (mg/h)
ka3_max_mv  <- log(1 + 0.0465^2)
ka3_max_m   ~  dlnorm(meanlog=log(0.164),   varlog=ka3_max_mv)  # (mg/h)
kamt1_50_mv <- log(1 + 0.0383^2)
kamt1_50_m  ~  dlnorm(meanlog=log(120),     varlog=kamt1_50_mv) # (mg)
kamt3_50_mv <- log(1 + 0.0952^2)
kamt3_50_m  ~  dlnorm(meanlog=log(21.4),    varlog=kamt3_50_mv) # (mg)
gamma_mv    <- log(1 + 0.0165^2)
gamma_m     ~  dlnorm(meanlog=log(1.44),    varlog=gamma_mv)    # unitless
CL_mv       <- log(1 + 0.0216^2)
CL_m        ~  dlnorm(meanlog=log(3.84),    varlog=CL_mv)       # (L/hr)
V_m         <- 1960   # (L)
##
## REFERENCE pop (inter-individual) SDs (with prior if not fixed)
##
## F2_sd
omega2    <- 0.064 / (0.168 * (1 - 0.168)) # Samtani eq. 4 inverted
F2_sd     ~  dnorm(mean = omega2, sd = 0.02*omega2)
F2_v      <- F2_sd^2
## F3_v, Samtani eq. 4 inverted
F3_v      <- (0.854 / (1 - 0.209))^2
##
ka1_pp1m_cv_v <- log(1 + 0.03^2)
ka1_pp1m_cv   ~  dlnorm(meanlog=log(0.590), varlog=ka1_pp1m_cv_v)
ka1_pp1m_v    <- log(1 + ka1_pp1m_cv^2)
##
ka1_max_cv_v  <- log(1 + 0.0501^2)
ka1_max_cv    ~  dlnorm(meanlog=log(0.827), varlog=ka1_max_cv_v)
ka1_max_v     <- log(1 + ka1_max_cv^2)
##
ka3_max_v     <- 0
##
kamt1_50_cv_v <- log(1 + 0.101^2)
kamt1_50_cv   ~  dlnorm(meanlog=log(0.500), varlog=kamt1_50_cv_v)
kamt1_50_v    <- log(1 + kamt1_50_cv^2)
##
kamt3_50_cv_v <- log(1 + 0.142^2)
kamt3_50_cv   ~  dlnorm(meanlog=log(0.867), varlog=kamt3_50_cv_v)
kamt3_50_v    <- log(1 + kamt3_50_cv^2)
##
gamma_v       <- 0
##
CL_cv_v       <- log(1 + 0.0317^2)
CL_cv         ~  dlnorm(meanlog=log(0.357), varlog=CL_cv_v)
CL_v          <- log(1 + CL_cv^2)
##
V_v           <- log(1 + 0.628^2)
##
## TEST group population mean (with prior if not fixed)
## Delta is a vector containing the factor of difference between the
## ref and test compounds for the 6 drug-release  parameters
## 1 = no difference.
## Order in Delta: f_3, k_as3,max, k_ar3,max, k_as3,50, k_ar3,50, Gamma.
## If Do_fit > 0.5, sample the first 3 Delta.
if (Do_fit > 0.5) {
  logDelta2 ~  dnorm(mean=logmeanD2, var=logvarD2)
  Delta[2]  <- exp(logDelta2)
}
F3t_m_T      <- logit(0.209 * Delta[1]) # transformed F3 with delta
ka1_max_m_T  <- ka1_max_m   * Delta[2]  # (mg/h)
ka3_max_m_T  <- ka3_max_m   * Delta[3]  # (mg/h)
kamt1_50_m_T <- kamt1_50_m  * Delta[4]  # (mg)
kamt3_50_m_T <- kamt3_50_m  * Delta[5]  # (mg)
gamma_m_T    <- gamma_m     * Delta[6]  # unitless
##
```

```
## if F3_m is modified then F3_v is modified:
if (SamEq3) { # see above
   F3_v_T    <- (0.854 / (1 - 0.209 * Delta[1]))^2
} else {
   F3_v_T    <- (abs(F3t_m_T) * 0.854)^2
}
##
## measurement error variance in log for plasma concentration, ng/ml
## if we want uncertainty on the residual error we should use:
res_cv_v    <- log(1 + 0.321^2)
res_cv      ~  dlnorm(meanlog=log(0.306), varlog=res_cv_v)
sigma2      <- log(1 + res_cv^2)
## simpler is:
## sigma2 <- log(1 + 0.306^2)

## for each REFERENCE subject
for (i in 1:nsubjects_per_arm) {
  tmp2[i]     ~  dnorm(mean = F2t_m, var = F2_v)
  F2[i]       <- ilogit(tmp2[i])
  tmp3[i]     ~  dnorm(mean = F3t_m, var = F3_v)
  F3[i]       <- ilogit(tmp3[i])
  ka_PP1M[i]  ~  dlnorm(meanlog=log(ka1_pp1m_m), varlog=ka1_pp1m_v)  # (1/hr)
  ka1_max[i]  ~  dlnorm(meanlog=log(ka1_max_m),  varlog=ka1_max_v)   # (mg/h)
  ka3_max[i]  <- ka3_max_m                                           # (mg/h)
  kamt1_50[i] ~  dlnorm(meanlog=log(kamt1_50_m), varlog=kamt1_50_v)  # (mg)
  kamt3_50[i] ~  dlnorm(meanlog=log(kamt3_50_m), varlog=kamt3_50_v)  # (mg)
  gamma[i]    <- gamma_m
  CL[i]       ~  dlnorm(meanlog=log(CL_m),       varlog=CL_v)        # (L/hr)
  V[i]        ~  dlnorm(meanlog=log(V_m),        varlog=V_v)         # (L)
  ##
  ## quantity at t 0
  Q_cen_0[i]  ~  dlnorm(meanlog=log(Q_cen_0_mean), sdlog = log(Q_cen_0_sd))
  ##
  ## likelihood for concentration measurements
  ## Call the ODE solver to get predictions for each subject
  ## predictions are plasma concentrations, in ng/ml
  Conc[i, 1:ntimes] <- nimble_ode(y[1:nstates], times[1:ntimes],
                                  c(Dose_PP1M, F2[i], Duration_2,
                                    ka_PP1M[i], ka1_max[i], ka3_max[i],
                                    kamt1_50[i], kamt3_50[i], gamma[i],
                                    CL[i], V[i], PP3M_start, F3[i],
                                    Dose_PP3M, Q_cen_0[i]))
  ## data likelihood
  for (j in 1:ntimes) {
    C_plasma_obs[i,j] ~ dlnorm(meanlog=log(Conc[i,j]), varlog=sigma2)
  }
}
## for each TEST subject
for (i in (1+nsubjects_per_arm):(2*nsubjects_per_arm)) {
  tmp2[i]     ~  dnorm(mean = F2t_m, var = F2_v)
  F2[i]       <- ilogit(tmp2[i])
  tmp3[i]     ~  dnorm(mean = F3t_m_T, var = F3_v_T)
  F3[i]       <- ilogit(tmp3[i])
  ka_PP1M[i]  ~  dlnorm(meanlog=log(ka1_pp1m_m),   varlog=ka1_pp1m_v) # (1/hr)
  ka1_max[i]  ~  dlnorm(meanlog=log(ka1_max_m_T),  varlog=ka1_max_v)  # (mg/h)
  ka3_max[i]  <- ka3_max_m_T                                          # (mg/h)
  kamt1_50[i] ~  dlnorm(meanlog=log(kamt1_50_m_T), varlog=kamt1_50_v) # (mg)
  kamt3_50[i] ~  dlnorm(meanlog=log(kamt3_50_m_T), varlog=kamt3_50_v) # (mg)
  gamma[i]    <- gamma_m_T
  CL[i]       ~  dlnorm(meanlog=log(CL_m),         varlog=CL_v)       # (L/hr)
  V[i]        ~  dlnorm(meanlog=log(V_m),          varlog=V_v)        # (L)
  ##
  ## quantity at t 0
  Q_cen_0[i]  ~  dlnorm(meanlog=log(Q_cen_0_mean), sdlog = log(Q_cen_0_sd))
```

```
    ##
    ## likelihood for concentration measurements
    ## Call the ODE solver to get predictions for each subject
    ## predictions are plasma concentrations, in ng/ml
    Conc[i, 1:ntimes] <- nimble_ode(y[1:nstates], times[1:ntimes],
                                    c(Dose_PP1M, F2[i], Duration_2,
                                      ka_PP1M[i], ka1_max[i], ka3_max[i],
                                      kamt1_50[i], kamt3_50[i], gamma[i],
                                      CL[i], V[i], PP3M_start, F3[i],
                                      Dose_PP3M, Q_cen_0[i]))
    ## data likelihood
    for (j in 1:ntimes) {
      C_plasma_obs[i,j] ~ dlnorm(meanlog=log(Conc[i,j]),varlog=sigma2)
    }
  }
}) # End myNimbleCode
## End.
```

13.5 TOST code in R

```
## TOST test function: takes ref and test PK parameters (Cmax...) and
## returns TRUE if they are bioequivalent

myTOST = function(X.ref, X.test) {
  N = length(X.ref)
  ##
  ## means in log space
  mu.ref  = mean(log(X.ref))
  mu.test = mean(log(X.test))
  ##
  ## variances in log space
  var.ref  = var(log(X.ref))
  var.test = var(log(X.test))
  ##
  logmeans.diff = mu.test - mu.ref
  logvars.mean  = (var.ref + var.test) / 2
  ##
  ## delta = t.variate * SE
  delta = qt(0.95, df = 2*N - 2) * sqrt(logvars.mean * 2 / N)
  CL_lo = logmeans.diff - delta
  CL_up = logmeans.diff + delta
  ##
  X.yes = (exp(CL_lo) > 0.8) && (exp(CL_up) < 1.25) # true or false...
  return(X.yes)
}
## End.
```

13.6 C_{max} and AUC data-based calculations code in R

```
## Compute Cmax and AUC given a vector of times and a matrix of concentration
## measurements at some times for different subjects (times in row, subjects
## in columns)

## Compute Cmax for different subjects
get.Cmax = function(conc) {
  return(apply(conc, MARGIN=2, FUN=max))
}

## Compute AUC for each subject
get.AUC = function(times, conc) {
  Ns  = dim(conc)[2]
  AUC = rep(0, Ns)
  Nt  = length(times)
  dx  = diff(times)
```

```
  for (i in 1:Ns) {
    AUC[i] = sum((conc[-Nt,i] + conc[-1,i]) * dx) / 2
  }
  return(AUC)
}
## End.
```

13.7 Structural model C code (v5)

```
/* compile within R with system("R CMD SHLIB PP3M_model.c")
   V05: Compute central concentration AUC by integration
*/
#include <R.h>

#define Nparms 12

static double parms[Nparms];

/* A trick to keep up with the parameters */
#define Dose_PP1M    parms[0]
#define F2           parms[1]
#define Duration_2   parms[2]
#define ka_PP1M      parms[3]
#define ka1_max      parms[4]
#define ka3_max      parms[5]
#define kamt1_50     parms[6]
#define kamt3_50     parms[7]
#define gamma        parms[8]
#define CL           parms[9]
#define V            parms[10]
#define PP3M_start   parms[11]

/* initializer: same name as the dll (without extension) */
void PP13M_model_v05(void (* odeparms)(int *, double *))
{
  int N = Nparms;
  odeparms(&N, parms);
}

/* Derivatives */
void derivs(int *neq, double *t, double *y, double *ydot, double *yout, int*ip)
{
  double Ke;

  // State variables
  // Q_depot_s1  = y[0] # quantity (mg) in PP1M slow absorption depot
  // Q_depot_s3  = y[1] # quantity (mg) in PP3M slow absorption depot
  // Q_depot_r3  = y[2] # quantity (mg) in PP3M fast absorption depot
  // Q_central   = y[3] # quantity (mg) in central compartment
  // AUC_central = y[4] # integral of y[3]

  // ODEs
  // Quantity in PP1M depot slow absorption
  ydot[0] = -ka_PP1M * y[0];

  // Quantities in PP3M depots
  if (*t < PP3M_start) { // use PP1M model, PP3M model differentials are null
    // Quantity in PP3M depot slow absorption
    ydot[1] = 0;
    // Quantity in PP3M depot rapid absorption
    ydot[2] = 0;
  }
```

```
  else { // use PP1M and PP3M models concurrently
    // Quantity in PP3M depot slow absorption
    ydot[1] = -ka1_max * pow(y[1], gamma) /
               (pow(kamt1_50, gamma) + pow(y[1], gamma));
    // Quantity in PP3M depot rapid absorption
    ydot[2] = -ka3_max * y[2] / (kamt3_50 + y[2]);
  }

  // Quantity in central compartment
  // clearance from central
  Ke = CL / V;
  // hard-code the zero-order inputs after PP1M injections
  if (((0 <= *t)    && (*t < 319))  || ((672 <= *t)  && (*t < 991)) ||
      ((1344 <= *t) && (*t < 1663)) || ((2016 <= *t) && (*t < 2335))) {
    ydot[3] = F2 * Dose_PP1M / Duration_2
            -(ydot[0] + ydot[1] + ydot[2]) - Ke * y[3];
  }
  else {
    ydot[3] = -(ydot[0] + ydot[1] + ydot[2]) - Ke * y[3];
  }

  // Q central AUC
  ydot[4] = y[3];
}

/* End */
```

13.8 Statistical model in R (v16)

```
## R/Nimble code for PP1M/PP3M population model, from Magnusson
## v14: Sample Delta from its posterior to abbreviated trial distribution.
##      Posterior of log(Delta[2]) is well approximated by a normal.
## v15: Uses C model v5 which calculates AUC by integration.
## v16: Reset AUC_central at last dosing time.

library(nimble)

## compile C ODE model for deSolve
Cmodel.name = "PP13M_model_v05"
## system(paste0("R CMD SHLIB ", Cmodel.name, ".c"))
dyn.load(paste(Cmodel.name, .Platform$dynlib.ext, sep = ""))
## dyn.unload(paste(Cmodel.name, .Platform$dynlib.ext, sep = ""))


## ===========================================================================
## Define the model

## ---------------------------------------------------------------------------
## ODE solver: performs a simulation, given initial state values,
## output times and time-constant, scaled, parameters
## This is an R function
R_ode = function(y, times, parms) {
  ## parms: 1:Dose_PP1M, 2:F2,        3:Duration_2,  4:ka_PP1M,
  ##        5:ka1_max,   6:ka3_max,   7:kamt1_50,    8:kamt3_50,
  ##        9:gamma,    10:CL,       11:V,          12:PP3M_start,
  ##       13:F3,       14:Dose_PP3M, 15:Q_cen_0
  ## The last two parameters should not be passed to the ODE solver, they are
  ## used here only.
  ## State variables (y) initial conditions
  y = c("Q_depot_s1"  = 0,
        "Q_depot_s3"  = 0,
        "Q_depot_r3"  = 0,
        "Q_central"   = parms[15],
        "AUC_central" = 0)
  ## The doses are specified as "events" affecting the state variables
```

```
  ## dosing times (hours)
  dose_times = c(c(0, 4, 8, 12) + parms[3] / (24 * 7),
                 17, 29, 41, 53) * 24 * 7
  N_doses = length(dose_times)
  ## changing state variables at dosing times
  vars = c("Q_depot_s1", "Q_depot_s3", "Q_depot_r3")
  v1 = (1 -  parms[2])  * parms[1]     # value applied to Q_depot_s1
  v2 = (1 -  parms[13]) * parms[14]    # value applied to Q_depot_s3
  v3 =       parms[13]  * parms[14]    # value applied to Q_depot_r3
  ## Form the events table
  eventdat = data.frame(var = c(rep("Q_depot_s1", 4), rep(vars[2:3], 4),
                                "AUC_central"),
                        time = c(dose_times[1:4], rep(dose_times[5:8], each=2),
                                 dose_times[8]),
                        value = c(rep(v1, 4),
                                  rep(c(v2, v3), N_doses - 4), 0),
                        method = c(rep("add", 12), "replace"))
  ## Integrate numerically, with outputs at specified times
  results = deSolve::lsode(y, times, func="derivs", parms=parms[1:12],
                           rtol=1e-6, atol=1e-6, dllname="PP13M_model_v05",
                           initfunc = "PP13M_model_v05",
                           events=list(data=eventdat))
  ## results = result[which(results[,1] %in% times),] # weed out extra times
  nrow.res = dim(results)[[1]]
  if (results[nrow.res,1] < times[length(times)]) { ## integration failed
    return(rep(1E-30, 2))
  } else {
    ## central concentration AUC is computed over last dosing period,
    ## convert from hours * mg/L to week * ng/ml, return
    from = max(which(results[,1] == dose_times[N_doses]))
    Cmax = max(results[from:nrow.res,5])
    AUC = results[nrow.res,6] * 1E3 / parms[11] / 168
    return(as.numeric(c(Cmax, AUC)))
  }
} # end of R_ode model solver


## ----------------------------------------------------------------------------
## Nimble function with nimbleRcall. This is just a wrapper
## to call the R-coded "R_ode" model solver from inside the "myNimbleCode" core
## function (defined next). It has to match the format of the "R_ode" function.
nimble_ode = nimbleRcall(
  prototype = function(
    y     = double(1), # vector
    times = double(1), # vector
    parms = double(1)  # vector
  ) {},
  returnType = double(1), # outcome is a vector
  Rfun = 'R_ode'
)

## ----------------------------------------------------------------------------
## Hierarchical core Nimble (BUGS) code
myNimbleCode = nimbleCode({ ## BUGS (extended) code
  ##
  ## REFERENCE group population mean (with prior if not fixed)
  F2t_m       <- logit(0.168) # From Samtani paper Table III.
  # from online resource 3 Magnusson, F2 = 0.153
  F3t_m       <- logit(0.209) # transformed F3
  ka1_pp1m_mv <- log(1 + 0.02^2)
  ka1_pp1m_m  ~  dlnorm(meanlog=log(4.88E-4), varlog=ka1_pp1m_mv) # (1/h)
  ka1_max_mv  <- log(1 + 0.0696^2)
  ka1_max_m   ~  dlnorm(meanlog=log(0.0904),  varlog=ka1_max_mv)  # (mg/h)
  ka3_max_mv  <- log(1 + 0.0465^2)
```

```
ka3_max_m   ~  dlnorm(meanlog=log(0.164),   varlog=ka3_max_mv)  # (mg/h)
kamt1_50_mv <- log(1 + 0.0383^2)
kamt1_50_m  ~  dlnorm(meanlog=log(120),     varlog=kamt1_50_mv) # (mg)
kamt3_50_mv <- log(1 + 0.0952^2)
kamt3_50_m  ~  dlnorm(meanlog=log(21.4),    varlog=kamt3_50_mv) # (mg)
gamma_mv    <- log(1 + 0.0165^2)
gamma_m     ~  dlnorm(meanlog=log(1.44),    varlog=gamma_mv)    # unitless
CL_mv       <- log(1 + 0.0216^2)
CL_m        ~  dlnorm(meanlog=log(3.84),    varlog=CL_mv)       # (L/hr)
V_m         <- 1960   # (L)
##
## REFERENCE pop (inter-individual) SDs (with prior if not fixed)
##
## F2_sd
omega2    <- 0.064 / (0.168 * (1 - 0.168)) # Samtani eq. 4 inverted
F2_sd     ~  dnorm(mean = omega2, sd = 0.02*omega2)
F2_v      <- F2_sd^2
## F3_v, Samtani eq. 4 inverted
F3_v      <- (0.854 / (1 - 0.209))^2
##
ka1_pp1m_cv_v <- log(1 + 0.03^2)
ka1_pp1m_cv   ~  dlnorm(meanlog=log(0.590), varlog=ka1_pp1m_cv_v)
ka1_pp1m_v    <- log(1 + ka1_pp1m_cv^2)
##
ka1_max_cv_v  <- log(1 + 0.0501^2)
ka1_max_cv    ~  dlnorm(meanlog=log(0.827), varlog=ka1_max_cv_v)
ka1_max_v     <- log(1 + ka1_max_cv^2)
##
ka3_max_v     <- 0
##
kamt1_50_cv_v <- log(1 + 0.101^2)
kamt1_50_cv   ~  dlnorm(meanlog=log(0.500), varlog=kamt1_50_cv_v)
kamt1_50_v    <- log(1 + kamt1_50_cv^2)
##
kamt3_50_cv_v <- log(1 + 0.142^2)
kamt3_50_cv   ~  dlnorm(meanlog=log(0.867), varlog=kamt3_50_cv_v)
kamt3_50_v    <- log(1 + kamt3_50_cv^2)
##
gamma_v       <- 0
##
CL_cv_v       <- log(1 + 0.0317^2)
CL_cv         ~  dlnorm(meanlog=log(0.357), varlog=CL_cv_v)
CL_v          <- log(1 + CL_cv^2)
##
V_v           <- log(1 + 0.628^2)
##
## TEST group population mean (with prior if not fixed)
## Delta is a vector containing the factor of difference between the
## ref and test compounds for the 6 drug-release  parameters
## 1 = no difference.
## Order in Delta: f_3, k_as3,max, k_ar3,max, k_as3,50, k_ar3,50, Gamma.
## If Do_fit > 0.5, sample the first 3 Delta.
if (Do_fit > 0.5) {
  logDelta2 ~  dnorm(mean=logmeanD2, var=logvarD2)
  Delta[2]  <- exp(logDelta2)
}
else {
  logDelta2 ~  dnorm(mean=0.34882, sd=0.17475) # abb. trial posterior
  Delta[2]  <- exp(logDelta2)
}
F3t_m_T      <- logit(0.209 * Delta[1]) # transformed F3 with delta
ka1_max_m_T  <- ka1_max_m   * Delta[2]  # (mg/h)
ka3_max_m_T  <- ka3_max_m   * Delta[3]  # (mg/h)
kamt1_50_m_T <- kamt1_50_m  * Delta[4]  # (mg)
kamt3_50_m_T <- kamt3_50_m  * Delta[5]  # (mg)
```

```
gamma_m_T    <- gamma_m     * Delta[6]  # unitless
##
## if F3_m is modified then F3_v is modified:
if (SamEq3) { # see above
   F3_v_T    <- (0.854 / (1 - 0.209 * Delta[1]))^2
} else {
   F3_v_T    <- (abs(F3t_m_T) * 0.854)^2
}
##
## measurement error variance in log for plasma concentration, ng/ml
## if we want uncertainty on the residual error we should use:
res_cv_v    <- log(1 + 0.321^2)
res_cv      ~  dlnorm(meanlog=log(0.306), varlog=res_cv_v)
sigma2      <- log(1 + res_cv^2)
## simpler is:
## sigma2 <- log(1 + 0.306^2)

## for each REFERENCE subject
for (i in 1:nsubjects_per_arm) {
  tmp2[i]     ~  dnorm(mean = F2t_m, var = F2_v)
  F2[i]       <- ilogit(tmp2[i])
  tmp3[i]     ~  dnorm(mean = F3t_m, var = F3_v)
  F3[i]       <- ilogit(tmp3[i])
  ka_PP1M[i]  ~  dlnorm(meanlog=log(ka1_pp1m_m), varlog=ka1_pp1m_v)  # (1/hr)
  ka1_max[i]  ~  dlnorm(meanlog=log(ka1_max_m),  varlog=ka1_max_v)   # (mg/h)
  ka3_max[i]  <- ka3_max_m                                           # (mg/h)
  kamt1_50[i] ~  dlnorm(meanlog=log(kamt1_50_m), varlog=kamt1_50_v)  # (mg)
  kamt3_50[i] ~  dlnorm(meanlog=log(kamt3_50_m), varlog=kamt3_50_v)  # (mg)
  gamma[i]    <- gamma_m
  CL[i]       ~  dlnorm(meanlog=log(CL_m),       varlog=CL_v)        # (L/hr)
  V[i]        ~  dlnorm(meanlog=log(V_m),        varlog=V_v)         # (L)
  ##
  ## quantity at t 0
  Q_cen_0[i]  ~  dlnorm(meanlog=log(Q_cen_0_mean), sdlog = log(Q_cen_0_sd))
  ##
  ## Call the ODE solver to get AUC_central for each subject
  ## prediction is plasma concentration AUC, in week * ng/ml
  Cmax_AUC[i,1:2] <- nimble_ode(y[1:nstates], times[1:ntimes],
                                c(Dose_PP1M, F2[i], Duration_2,
                                  ka_PP1M[i], ka1_max[i], ka3_max[i],
                                  kamt1_50[i], kamt3_50[i], gamma[i],
                                  CL[i], V[i], PP3M_start, F3[i],
                                  Dose_PP3M, Q_cen_0[i]))
}

## for each TEST subject
for (i in (1+nsubjects_per_arm):(2*nsubjects_per_arm)) {
  tmp2[i]     ~  dnorm(mean = F2t_m, var = F2_v)
  F2[i]       <- ilogit(tmp2[i])
  tmp3[i]     ~  dnorm(mean = F3t_m_T, var = F3_v_T)
  F3[i]       <- ilogit(tmp3[i])
  ka_PP1M[i]  ~  dlnorm(meanlog=log(ka1_pp1m_m),   varlog=ka1_pp1m_v) # (1/hr)
  ka1_max[i]  ~  dlnorm(meanlog=log(ka1_max_m_T),  varlog=ka1_max_v)  # (mg/h)
  ka3_max[i]  <- ka3_max_m_T                                          # (mg/h)
  kamt1_50[i] ~  dlnorm(meanlog=log(kamt1_50_m_T), varlog=kamt1_50_v) # (mg)
  kamt3_50[i] ~  dlnorm(meanlog=log(kamt3_50_m_T), varlog=kamt3_50_v) # (mg)
  gamma[i]    <- gamma_m_T
  CL[i]       ~  dlnorm(meanlog=log(CL_m),         varlog=CL_v)       # (L/hr)
  V[i]        ~  dlnorm(meanlog=log(V_m),          varlog=V_v)        # (L)
  ##
  ## quantity at t=0
  Q_cen_0[i]  ~  dlnorm(meanlog=log(Q_cen_0_mean), sdlog = log(Q_cen_0_sd))
  ##
  ## Call the ODE solver to get AUC_central for each subject
```

```
    ## prediction is plasma concentration AUC, in week * ng/ml
    Cmax_AUC[i,1:2] <- nimble_ode(y[1:nstates], times[1:ntimes],
                                  c(Dose_PP1M, F2[i], Duration_2,
                                    ka_PP1M[i], ka1_max[i], ka3_max[i],
                                    kamt1_50[i], kamt3_50[i], gamma[i],
                                    CL[i], V[i], PP3M_start, F3[i],
                                    Dose_PP3M, Q_cen_0[i]))
  }

}) # End myNimbleCode

## End.
```

13.9 Fully Bayesian workflow in R (v16)

```
## Fully Bayesian model-based VBE
## v1: Based on paliperidone palmitate VBE workflow v4.
##     Uses statistical model v14, Cmax and AUC are data-based.
## v2: Uses statistical model v15, which calculates AUC over the last
##     dosing period by integration.
## v3: Uses statistical model v16, which calculates AUC over the last
##     dosing period by integration an calculates Cmax with many time points.

IDtag = "_3" # version number

## ====================================================================
## Compile and run the model for many trial of many subjects,
## get Cmax and AUC.

## Parallelize
library(parallel)
N.cores = 5 # detectCores()
this_cluster = makeCluster(N.cores)

## Create a function with all the needed code
run_allcode <- function(seed) {
  ##
  library(nimble)
  source("Statistical model v16.R")
  ## source("Cmax_AUC.R")
  ##
  N.subjects.v = 1000 # number of virtual subjects per arm
  ##
  Hr1 = 1 / (24 * 7) # one hour in weeks
  times = c(0, seq(53, 65, (65-53)/100)) * 168 # (hours)
  N.times = length(times)
  ##
  dose_pp1m = 150
  dose_pp3m = 525
  ##
  Q_cen_0_mean = 30  # geo mean
  Q_cen_0_sd   = 1.5 # geo SD
  ##
  data  = list()
  ##
  inits = list(Q_cen_0_mean = Q_cen_0_mean, # (mg)
               Q_cen_0_sd   = Q_cen_0_sd,   # (mg)
               Dose_PP1M    = dose_pp1m[1], # (mg)
               Dose_PP3M    = dose_pp3m[1], # (mg)
               Delta        = rep(1, 6))
  ##
  constants = list(nsubjects_per_arm = N.subjects.v,
                   Do_fit     = 0,            # 0: no Delta fit, > 0: fit
                   SamEq3     = 1,            # Boolean, leave at 1
                   Duration_2 = 319,          # (h)
```

```
                   PP3M_start = 17*7*24,      # (h)
                   ntimes     = length(times),
                   times      = times,
                   nstates    = 5)
  ##
  Rmodel = nimbleModel(myNimbleCode, constants, data, inits, calculate=F)
  ## Cmodel = Rmodel
  Cmodel = compileNimble(Rmodel, showCompilerOutput=F)
  ##
  N.mtc.v  = 200 # number of simulated virtual trials
  Node.names = Cmodel$getNodeNames(includeData=T)
  Delta.Cmax = Delta.AUC = rep(0, N.mtc.v)
  ##
  for (i in 1:N.mtc.v) {
    ## Simulate the trial
    Cmodel$simulate(nodes = Node.names)
    ## all.data = values(Cmodel, "C_plasma_obs")
    ## all.data = matrix(all.data, ncol=N.times, byrow = F) # subjects by row
    all.res = values(Cmodel, "Cmax_AUC")
    ##
    ## Cmax values
    Cmaxs      = all.res[1:(2*N.subjects.v)]
    ref.Cmaxs  = Cmaxs[1:N.subjects.v] # reference arm of the trial
    test.Cmaxs = Cmaxs[(N.subjects.v + 1):(2 * N.subjects.v)] # test arm
    ##
    Cmax.ref.mean  = mean(log(ref.Cmaxs))
    Cmax.ref.sd    = sd  (log(ref.Cmaxs))
    Cmax.test.mean = mean(log(test.Cmaxs))
    Cmax.test.sd   = sd  (log(test.Cmaxs))
    Delta.Cmax[i]  = Cmax.test.mean - Cmax.ref.mean
    ##
    ## AUC values
    AUCs       = all.res[(2*N.subjects.v + 1):(4*N.subjects.v)]
    ref.AUCs   = AUCs[1:N.subjects.v] # reference arm of the trial
    test.AUCs  = AUCs[(N.subjects.v + 1):(2 * N.subjects.v)] # test arm
    ##
    AUC.ref.mean   = mean(log(ref.AUCs))
    AUC.ref.sd     = sd  (log(ref.AUCs))
    AUC.test.mean  = mean(log(test.AUCs))
    AUC.test.sd    = sd  (log(test.AUCs))
    Delta.AUC[i]   = AUC.test.mean - AUC.ref.mean
  }
  ##
  save(list=c("N.mtc.v", "N.subjects.v", "Delta.Cmax", "Delta.AUC",
              "Cmax.ref.mean", "Cmax.test.mean", "Cmax.ref.sd", "Cmax.test.sd",
              "AUC.ref.mean",  "AUC.test.mean",  "AUC.ref.sd",  "AUC.test.sd"),
       file=paste0("Delta Cmax & AUC model based_", seed,".Rsave"))
  return(1)
  ##
} ## End run_allcode

t.start = Sys.time()
chain_output <- parLapply(cl=this_cluster, X=1:N.cores, fun=run_allcode)
t.end = Sys.time()
t.end - t.start

## Close the cluster when you're done with it.
stopCluster(this_cluster)

## Post processing
## Compute probabilities of declaring BE for various trial sizes
for (j in 1:N.cores) { # for each Monte Carlo block
  ##
  load(file=paste0("Delta Cmax & AUC model based_", j,".Rsave"))
```

```
  ##
  if (j == 1) {
    Delta.Cmax.all = Delta.Cmax
    Delta.AUC.all  = Delta.AUC
  } else {
    Delta.Cmax.all  = c(Delta.Cmax.all, Delta.Cmax)
    Delta.AUC.all   = c(Delta.AUC.all,  Delta.AUC)
  }
}
length(Delta.Cmax.all)

Ratio.Cmax = exp(Delta.Cmax.all)
Ratio.AUC  = exp(Delta.AUC.all)

## Plot Delta Cmax distribution
xlims = c(0.6, 1.6)
pdf(paste0("Delta Cmax distribution", IDtag,".pdf"))
hist(Ratio.Cmax, breaks=10, axes=F, xlim=xlims,
     main="", xlab="Cmax geometric mean test to reference ratio")
rect(min(xlims), 0, 0.8,        N.mtc.v, dens=10, col="red")
rect(1.25,       0, max(xlims), N.mtc.v, dens=10, col="red")
axis(1)
P.BE = (length(which((Ratio.Cmax <= 0.8))) +
        length(which((Ratio.Cmax >= 1.25)))) / (N.cores * N.mtc.v)
legend(0.8, N.mtc.v * 1.1,
       substitute(bar(P[BE]) == list(x), list(x = P.BE)), bty="n")
dev.off()

mean(Ratio.Cmax)
sd(Ratio.Cmax)

## Plot Delta AUC distribution
xlims = c(0.6, 1.6)
pdf(paste0("Delta AUC distribution", IDtag,".pdf"))
hist(Ratio.AUC, breaks=10, axes=F, xlim=xlims,
     main="", xlab="AUC geometric mean test to reference ratio")
rect(min(xlims), 0, 0.8,        N.mtc.v, dens=10, col="red")
rect(1.25,       0, max(xlims), N.mtc.v, dens=10, col="red")
axis(1)
P.BE = (length(which((Ratio.AUC <= 0.8))) +
        length(which((Ratio.AUC >= 1.25)))) / (N.cores * N.mtc.v)
legend(0.8, N.mtc.v * 1.1,
       substitute(bar(P[BE]) == list(x), list(x = P.BE)), bty="n")
dev.off()

mean(Ratio.AUC)
sd(Ratio.AUC)

## Correlation plot
pdf("Correlation Ratio Cmax - Ratio AUC.pdf")
par(mar=c(5,5,2,2))
xlims = c(0.7, 1.6)
plot(Ratio.Cmax, Ratio.AUC, las=1, xlim=xlims, ylim=xlims, type="n",
     xlab="", ylab="")
rect(0,    0,    0.8, 2,   lty=0, col="lightpink")
rect(1.25, 0,    2,   2,   lty=0, col="lightpink")
rect(0,    0,    2,   0.8, lty=0, col="lightpink")
rect(0,    1.25, 2,   2,   lty=0, col="lightpink")
## rect(0,    0,    0.8, 2,   dens=10, col="red")
## rect(1.25, 0,    2,   2,   dens=10, col="red")
## rect(0,    0,    2,   0.8, dens=10, col="red")
## rect(0,    1.25, 2,   2,   dens=10, col="red")
par(new=T)
plot(Ratio.Cmax, Ratio.AUC, las=1, xlim=xlims, ylim=xlims,
     xlab=expression(delta[C[max]]), ylab=expression(delta[C[AUC]]))
```

```
dev.off()

## End.
```

13.10 Fully Bayesian safe-space calculations in R

```
## Fully Bayesian model-based VBE, safe-space calculations
## v1: based on workflow v4.
## v2: try to go faster by starting from non-BE

IDtag = "_2" # version number
IDrun = 6    # run number

## =========================================================================
## Compile and run the model for many trial of many subjects,
## get Cmax and AUC.

## Parallelize
library(parallel)
N.cores = 8 # detectCores()
this_cluster = makeCluster(N.cores)

## Create a function with all the needed code
run_allcode <- function(seed, N.cores, IDrun) {
  ##
  Delta1.vals     = seq(1.5, 0.8, -0.2/7)[9:22]
  Delta2.vals     = seq(1.5, 1, -0.01)
  ##
  library(nimble)
  source("Statistical model v16.R")
  ##
  N.subjects.v = 1000 # number of virtual subjects per arm
  ##
  Hr1 = 1 / (24 * 7) # one hour in weeks
  times = c(0, seq(53, 65, (65-53)/100)) * 168 # (hours)
  N.times = length(times)
  ##
  Q_cen_0_mean = 30  # geo mean
  Q_cen_0_sd   = 1.5 # geo SD
  ##
  dose_pp1m = 150
  dose_pp3m = 525
  ##
  data  = list()
  ##
  inits = list(Q_cen_0_mean = Q_cen_0_mean, # (mg)
               Q_cen_0_sd   = Q_cen_0_sd,   # (mg)
               Dose_PP1M    = dose_pp1m[1], # (mg)
               Dose_PP3M    = dose_pp3m[1], # (mg)
               Delta        = rep(1,6))
  ##
  constants = list(nsubjects_per_arm = N.subjects.v,
                   Do_fit     = 0,            # 0: no Delta fit, > 0: fit
                   SamEq3     = 1,            # Boolean, leave at 1
                   Duration_2 = 319,          # (h)
                   PP3M_start = 17*7*24,      # (h)
                   ntimes     = length(times),
                   times      = times,
                   nstates    = 5)
  ##
  Rmodel = nimbleModel(myNimbleCode, constants, data, inits, calculate=F)
  ## Cmodel = Rmodel
  Cmodel = compileNimble(Rmodel, showCompilerOutput=F)
```

```
##
Node.names = Cmodel$getNodeNames(includeData=T)
##
Cmodel$Delta[1] = Delta1.vals[seed]
index.D2        = which(Node.names == "Delta[2]")
N.delta2        = length(Delta2.vals)
Delta           = matrix(0, nrow=N.delta2, ncol=6)
##
BE = rep(NA, N.delta2)
N.mtc.v  = 1000 # number of simulated virtual trials
stop_after_this_trial = FALSE
##
for (j in 1:N.delta2) {
  ##
  Cmodel$Delta[2] = Delta2.vals[j]
  Delta[j,] = Cmodel$Delta
  N.fail = 0
  for (i in 1:N.mtc.v) {
    ## Simulate the trial
    Cmodel$simulate(nodes = Node.names[-index.D2]) # Delta[2] not sampled
    all.res = values(Cmodel, "Cmax_AUC")
    ##
    ## Cmax values
    Cmaxs      = all.res[1:(2*N.subjects.v)]
    ref.Cmaxs  = Cmaxs[1:N.subjects.v] # reference arm of the trial
    test.Cmaxs = Cmaxs[(N.subjects.v + 1):(2 * N.subjects.v)] # test arm
    ##
    Cmax.ref.mean  = mean(log(ref.Cmaxs))
    Cmax.ref.sd    = sd  (log(ref.Cmaxs))
    Cmax.test.mean = mean(log(test.Cmaxs))
    Cmax.test.sd   = sd  (log(test.Cmaxs))
    Delta.Cmax     = Cmax.test.mean - Cmax.ref.mean
    ##
    ## AUC values
    AUCs       = all.res[(2*N.subjects.v + 1):(4*N.subjects.v)]
    ref.AUCs   = AUCs[1:N.subjects.v] # reference arm of the trial
    test.AUCs  = AUCs[(N.subjects.v + 1):(2 * N.subjects.v)] # test arm
    ##
    AUC.ref.mean   = mean(log(ref.AUCs))
    AUC.ref.sd     = sd  (log(ref.AUCs))
    AUC.test.mean  = mean(log(test.AUCs))
    AUC.test.sd    = sd  (log(test.AUCs))
    Delta.AUC      = AUC.test.mean - AUC.ref.mean
    ##
    ## Compute BE for this trial
    Ratio.Cmax = exp(Delta.Cmax)
    Ratio.AUC  = exp(Delta.AUC)
    Cmax.yes   = ((Ratio.Cmax > 0.8) && (Ratio.Cmax < 1.25))
    AUC.yes    = ((Ratio.AUC  > 0.8) && (Ratio.AUC  < 1.25))
    BE.yes     = Cmax.yes && AUC.yes
    ##
    N.fail = N.fail + as.integer(!BE.yes) # cumulated number of failures
    print(paste("trial", i, ", fails:", N.fail))
    if (N.fail > N.mtc.v * 0.05) {
      ## this Delta vector will not pass, stop
      BE[j] = FALSE
      break
    } else {
      if (i == N.mtc.v) {
        ## this Delta vector lead to pass, do not look at further Delta's
        stop_after_this_trial = TRUE
      }
    }
  } # end for ith trial
  ##
```

```
    if (stop_after_this_trial) {
      BE[j] = TRUE
      break
    }
  } # end for jth Delta[2] value
  ##
  IDnum = seed + N.cores * (IDrun - 1)
  save(list=c("N.mtc.v", "N.subjects.v", "Delta", "BE"),
       file=paste0("Safe-space model based_", IDnum,".Rsave"))
  return(1)
  ##
} ## End run_allcode

t.start = Sys.time()
chain_output = parLapply(cl=this_cluster, X=1:N.cores, fun=run_allcode,
                         N.cores=N.cores, IDrun=IDrun)
t.end = Sys.time()
t.end - t.start

## Close the cluster when you're done with it.
stopCluster(this_cluster)

## ===============
## Post processing
n.start = 17 # real useful work starts at 17
n.end   = 52
for (j in n.start:n.end) { # for each Monte Carlo block
  ##
  if (!(j %in% 30:32)) { # weed out unneeded points
    myname = paste0("Safe-space model based_", j,".Rsave")
    load(file=myname)
    ##
    if (j == n.start) {
      Delta.all = Delta
      BE.all    = BE
    } else {
      Delta.all = rbind(Delta.all, Delta)
      BE.all    = c(BE.all, BE)
    }
  }
}

## Plot safe space
pdf("Safe space points plot v2.pdf")
mycols = rep("orange", length(BE.all))
mycols[which(BE.all == F)] = "red"
mycols[which(BE.all == TRUE)] = "green"
mylims = c(0.7,1.5)
plot(Delta.all[,1:2], las=1, xlim=mylims, ylim=mylims,
     xlab=expression(delta[1]), ylab=expression(delta[2]),
     col=mycols, pch=15, cex=0.55)
## lower limit
a = 1.265; b = -0.6 # y = a + b * x
abline(a, b, col="green")
## upper limit
a = 1.950; b = -0.47 # y = a + b * x
abline(a, b, col="green")
dev.off()

pdf("Safe space region plot v2.pdf")
mycols = rep("orange", length(BE.all))
mycols[which(BE.all == F)] = "red"
mycols[which(BE.all == TRUE)] = "green"
mylims = c(0.7,1.5)
```

```
plot(Delta.all[,1:2], las=1, xlim=mylims, ylim=mylims, type="n",
     xlab=expression(delta[1]), ylab=expression(delta[2]),
     col=mycols, pch=15, cex=0.55)
rect(0.7, 0.7, 1.5, 1.5, col="green", bord=NA)
## lower limit
a = 1.265; b = -0.6 # y = a + b * x
## abline(a, b, col="green")
polygon(c(0.7, 0.7, (0.7-a)/b), c(0.7, a+b*0.7, 0.7), col="red", bord="red")
## upper limit
a = 1.950; b = -0.47 # y = a + b * x
## abline(a, b, col="green")
polygon(c(1.5, 1.5, (1.5-a)/b), c(1.5, a+b*1.5, 1.5), col="red", bord="red")
dev.off()

## End.
```

14 References

1. Samtani MN, Vermeulen A, Stuyckens K. Population pharmacokinetics of intramuscular paliperidone palmitate in patients with schizophrenia: a novel once-monthly, long-acting formulation of an atypical antipsychotic. Clinical Pharmacokinetics. 2009;48:585-600 (PMID19725593).

2. Magnusson MO, Samtani MN, Plan EL, Jonsson EN, Rossenu S, Vermeulen A, et al. Population pharmacokinetics of a novel once-every 3 months intramuscular formulation of paliperidone palmitate in patients with schizophrenia. Clinical Pharmacokinetics. 2017;56:421-433 (PMID27743205).
